# Randomized, Placebo-Controlled, Double-Blind and Open-Label Studies in the Treatment and Prevention of Acute Diarrhea With *Enterococcus faecium* SF68

**DOI:** 10.3389/fmed.2020.00276

**Published:** 2020-06-19

**Authors:** Thomas Greuter, Martin C. Michel, Daniela Thomann, Harald Weigmann, Stephan R. Vavricka

**Affiliations:** ^1^Division of Gastroenterology and Hepatology, University Hospital Zurich, Zurich, Switzerland; ^2^Center for Gastroenterology, Zurich, Switzerland; ^3^Department of Pharmacology, Johannes Gutenberg University, Mainz, Germany; ^4^Sanofi-Aventis Schweiz GmbH, Vernier, Switzerland; ^5^Sanofi-Aventis Deutschland GmbH, Frankfurt, Germany; ^6^Department of Internal Medicine, GZO Zurich Regional Health Center, Wetzikon, Switzerland

**Keywords:** acute diarrhea, treatment, prevention, randomized-controlled trial, probiotics, *Enterococcus faecium*, SF68

## Abstract

*Enterococcus faecium* SF68® (SF68) is a licensed pharmaceutical for treatment and prevention of diarrhea in Austria, Italy and Switzerland. However, as for other probiotics, evidence for its efficacy is based on small to medium-sized studies. Four unpublished studies on the treatment of acute diarrhea and the prevention of antibiotic-associated diarrhea were analyzed: one randomized, double blind, placebo-controlled trial (RCT) for treatment (*n* = 1,143), one open-label study for treatment (*n* = 5,093), one RCT for prevention (*n* = 1,397) and one open-label study for prevention (*n* = 4,340). Patients in the treatment-arm and the open-label studies received SF68 (b.i.d. for the prevention studies, t.i.d. for the treatment trials) for 7 days. Primary end points were time to resolution of diarrhea (treatment) and percentage of development of diarrhea (prevention). The primary endpoint of the treatment study was met with a decreased time to resolution of diarrhea in SF68-treated patients compared to controls (median 3 vs. 4 days, *p* < 0.001). Time to resolution of secondary symptoms was also significantly reduced. Preventive treatment with SF68 was more effective than placebo with development of diarrhea in 8.6 vs. 16.2% (*p* < 0.001). Results from the open-label studies were consistent with the RCTs. The incidence of adverse events were low (1.1 and 1.4% in the RCT and 4.7 and 7.4% in the open-label studies). SF68 is effective and safe in the treatment of acute diarrhea and prevention of antibiotic-associated diarrhea.

## Introduction

Acute diarrhea is a frequent problem in an outpatient setting (1). Despite its mostly self-limiting disease course, acute diarrhea considerably affects morbidity and mortality (2). High mortality due to gastrointestinal infections is particularly seen in under-developed countries (3). Acute diarrhea further results in high direct and indirect health-care costs (4, 5). Except for some bacterial causes, no effective treatment exists, and current therapeutic strategies are mainly supportive.

Probiotics are increasingly used for both treatment of acute diarrhea and prevention of antibiotic-associated diarrhea, more so in children than adults. Most of to-date available data have emerged from clinical trials with *Lactobacillus rhamnosus GG* and *Saccharomyces boulardii* (6, 7). Current pediatric guidelines strongly recommend their therapeutic use as add-on to rehydration despite a low quality of evidence (8). So far conducted trials lack consistency, in some cases studies were neither blinded nor placebo controlled. Moreover, almost all of them consisted of small to moderate sample sizes. The evidence supporting the use of other probiotics such as *Lactobacillus reuteri* is even weaker (9, 10). Findings from trials with one probiotic cannot be extrapolated to another probiotic compound since different bacterial strains might have various effects. Therefore, no generalizable class statement can be made.

*Enterococcus faecium* SF68® (SF68) is a licensed pharmaceutical in Austria, Italy and Switzerland. Its indications are (i) treatment of acute infectious enteritis in children and adults; (ii) traveler‘s diarrhea; and (iii) prevention and treatment of antibiotic-associated diarrhea. In a comprehensive Cochrane Review on the treatment of acute infectious diarrhea with probiotics, four randomized controlled trials (RCT, *n* = 333) that evaluated SF68 qualified for inclusion demonstrating reduced risk for diarrhea lasting 4 days or longer (11). However, quality of the trials conducted with SF68 was insufficient with unclear or inadequate allocation concealment, no blinding in some trials and no or unclear intention to treat analyses (9). In addition, several trials published were not included in the analysis (12–16). There are no known side-effects to SF68, but due to the existence of vancomycin resistant Enterococci and the risk of its transmission, the safety of this genus has been questioned for a long time (17). Therefore, its use is currently not recommended by the European Society for Pediatric Gastroenterology, Hepatology and Nutrition (9). However, safety should be assessed at strain level and the safety of SF68 including absence of vancomycin resistance issues has been extensively documented and recently summarized in a comprehensive review (18).

Here, we report on two randomized-controlled, double blind trials and two open-label studies for the treatment of acute diarrhea and prevention of antibiotic-associated diarrhea with *Enteroccus faecium* SF68® in juvenile and adult patients, which have included a total of 11,973 patients.

## Materials and Methods

### Study Design

This was an analysis of two RCTs and two open-label studies for the treatment of acute diarrhea and prevention of antibiotic-associated diarrhea. All four studies were designed and completed prior to the publication of the European Good-Clinical-Practice (GCP) guidelines in 1989 and before an institutional review board was established at the institution of the primary investigator, the late Professor Mauro Moroni (University of Milan, Italy). However, the studies adhered to the rules and regulations applicable in Italy at that time. This manuscript is based on the study report and a detailed statistical analysis prepared by Gipharmex (Milan, Italy), which had been submitted to regulatory authorities in applicable countries and reported as a supplement in an educational magazine owned by Gipharmex (*Kole: Continuing Education in Gastroenterology*, Supplementary Datasheet 1). Sanofi-Aventis holds the marketing authorization for SF68 as well as the rights regarding use, publication of the findings from this study report. Raw data were not accessible since obligation to preserve records has been time-barred in the meantime. A summary of the data was presented at the 1986 World Congress of Gastroenterology (19).

### Patients and Data Collection

Subjects were recruited in the early 1980s at 150 hospitals and 653 outpatient general practitioner offices, all located in Italy. Patients recruited in the hospitals could be inpatients or outpatients.

For the treatment studies, the following inclusion criteria were applied: (i) adult or school-aged patients suffering from acute diarrhea without detectable signs of sepsis and/or involvement of other organs. Patients were excluded for: (i) history of food intolerance; and (ii) infectious diarrheas caused by Salmonella, Entamoeba, Giardia or other parasites. Patients were seen at baseline and had daily follow-up for a total of 7 days. The following items were documented: number of stools, consistency of stools, mucus in stool, abdominal pain, bloating, nausea, vomiting, fever, and adverse events. Further items on the case record forms at baseline included demographic parameters, assumed cause of diarrhea, treatment setting (inpatient vs. outpatient). In case of early discontinuation of treatment, reasons (early cure, adverse events, unrelated to treatment and disease) were documented.

**Figure 1:**
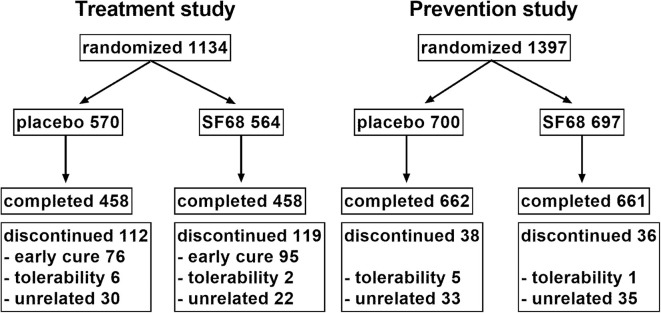
Study flow chart in the two randomized controlled trials.

For the prevention studies, inclusion criteria were: (i) adults or school-age patients receiving oral or intravenous antibiotic treatment. Patients were excluded for treatment with tetracyclines. Patients were seen at baseline and daily for the next 7 days. The following data were collected at baseline: demographic parameters, treatment setting (inpatient vs. outpatient), type of underlying infection (respiratory, urological, other), type of antibiotic. If diarrhea occurred, the following data were collected: (i) first day of diarrhea; (ii) severity graded into mild (up to 2–3 liquid or semiliquid stools without mucus/blood), moderate (4–6 mostly liquid stools, or stools with mucus), and severe (more than six liquid stools, mostly with mucus, sometimes with blood); (iii) duration of diarrhea; and (iv) presence of glossitis, labial and anal fissures. The study flow chart (RCT) is illustrated in Figure 1.

### Randomization and Treatment

Patients in the RCTs were randomized 1:1 into the treatment/prevention group or the placebo group. Subjects randomized into the interventional groups and patients in the open-label study received one capsule of SF68 (containing at least 75 million cells in dehydrated form) three times a day (treatment studies) and twice daily (prevention studies) for an intended duration of seven days.

### Measurements

The following primary endpoints were defined:

- Treatment studies: Time to resolution of diarrhea (defined as ≥3 stools per day or liquid or semiliquid stools)
- Prevention studies: Percent of patients developing antibiotic-associated diarrhea

Secondary endpoints for the treatment studies were duration of presence of (i) ≥3 stools, (ii) liquid or semiliquid stools, (iii) mucus on stool, and (iv) abdominal pain.

Secondary endpoints for the prevention studies were severity and duration of diarrhea, and occurrence of glossitis, labial and anal fissures.

### Study Power and Statistical Analysis

Retrospective power analysis based on an estimated median duration of acute diarrhea of 4 days yielded a sample size of 774 to be sufficient to detect a 20% relative reduction in the rate of patients with ongoing diarrhea at day 4 (from 50 to 40%) with a power of 80% (treatment study). A sample size of 1,228 patients was determined to detect a 30% relative reduction in the rate of new-onset diarrhea (based on an estimated incidence of 20%) with a power of 80% (prevention study). Effect sizes were chosen based on previous publications (20).

Original data had been analyzed by the Biometric and Medical Statistic Unit of Gipharmex using the statistical programs Framework (Ashton-Tate, Torrance, CA, USA) and SL-Micro software (Questionnaire Service Company, East Lansing, MI, USA). Independent statistical re-analyses based on the study report provided by Gipharmex were performed by the authors using Prism version 7.1 (Graphpad Software, La Jolla, CA, USA). Categorical data are summarized as the percentage of the group total. Comparison between categorical data was performed using χ^2^ test. For calculation of percentage of patients with ongoing diarrhea over time, Kaplan Meier curves were computed. Kaplan Meier curves were compared using log-rank test. For the purposes of this study, a *p* < 0.05 was considered statistically significant.

## Results

### Randomized Treatment Study

One thousand one hundred thirty-four patients were randomized 1:1 into the control (570 patients) and SF68 group (564 patients). Baseline demographics and disease characteristics were similar in the two groups (Table 1). All subjects were enrolled by hospital-based physicians, however 19.5% of patients had outpatient follow-up visits. 458 (placebo group) and 458 patients (SF68 arm) completed the seven-day treatment course (Figure 1).

**Table 1:** Baseline demographics and disease characteristics for the treatment studies.

		**Randomized**			**Open-label**
		**Placebo**	**SF68**	**total**	
Gender	Male	296 (51.9)	286 (50.7)	582 (51.3)	25,82 (50.7)
	Female	274 (48.1)	278 (49.3)	552 (48.7)	2,511 (49.3)
Age	<14 years	44 (7.7)	43 (7.6)	87 (7.7)	622 (12.2)
	14–20 years	18 (3.2)	13 (2.3)	31 (2.7)	438 (8.6)
	21–40 years	86 (15.1)	115 (20.4)	201 (17.7)	1,711 (33.6)
	41–60 years	154 (27.0)	141 (25.0)	295 (26.0)	1,441 (28.3)
	61–70 years	101 (17.7)	111 (19.7)	212 (18.7)	474 (9.3)
	>70 years	167 (29.3)	141 (25.0)	308 (27.2)	407 (8.0)
Treatment setting	Hospital	466 (81.8)	447 (79.3)	913 (80.5)	-
	Outpatient	104 (18.2)	117 (20.7)	221 (19.5)	5,093 (100.0)
Cause	Food	105 (18.4)	111 (19.7)	216 (19.0)	1,838 (36.1)
	Infection	103 (18.1)	82 (14.5)	185 (16.3)	922 (18.1)
	Iatrogenic	85 (14.9)	87 (15.4)	172 (15.2)	504 (9.9)
	Toxic	60 (10.5)	51 (9.0)	111 (9.8)	652 (12.8)
	Travel	14 (2.5)	16 (2.8)	30 (2.6)	484 (9.5)
	Unknown	203 (35.6)	217 (38.5)	420 (37.0)	693 (13.6)
Number of stools per day	3	128 (22.5)	130 (23.0)	258 (22.8)	1,181 (23.2)
	4	143 (25.1)	147 (26.1)	290 (25.6)	1,294 (15.4)
	5	90 (15.8)	87 (15.4)	177 (15.6)	1,069 (21.0)
	6	72 (12.6)	73 (12.9)	145 (12.8)	637 (12.5)
	>6	137 (24.0)	127 (22.5)	264 (23.3)	912 (17.9)
Stool consistency	Liquid	394 (69.1)	399 (70.7)	793 (69.9)	3,448 (67.7)
	Semi-liquid	176 (30.9)	165 (29.3)	341 (30.1)	1,645 (32.3)
Other signs/symptoms	Mucus on feces	220 (38.6)	205 (36.3)	425 (37.5)	2,052 (40.3)
	Abdominal pain	387 (67.9)	386 (68.4)	773 (62.2)	4,074 (80.0)
	Meteorism	334 (58.6)	360 (63.8)	694 (61.2)	3,351 (65.8)
	Nausea	241 (42.3)	246 (43.6)	487 (42.9)	2,124 (41.7)
	Vomiting	156 (27.4)	167 (29.6)	323 (28.5)	1,401 (27.5)
	Fever	220 (38.6)	197 (34.9)	417 (36.8)	1,625 (31.9)

*Data are shown as number of patients and % of group in parentheses*.

**Figure 2:**
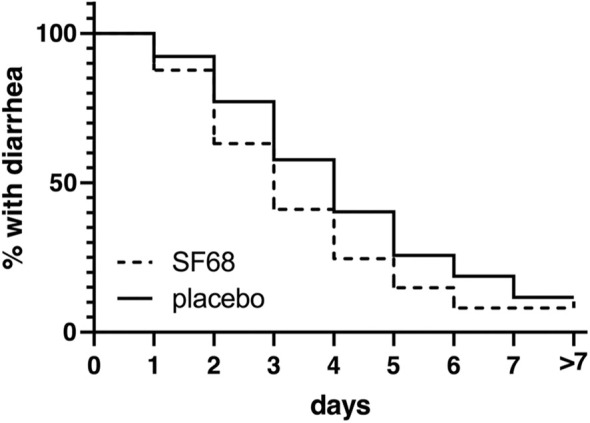
Time to resolution of diarrhea (defined as ≥3 stools per day or liquid or semiliquid stools) in the randomized-controlled treatment trial, shown as Kaplan-Meier curve [log-rank test *p* < 0.0001 for SF68 vs. placebo; hazard ratio 0.7363 (confidence interval 0.6491-0.8353)].

Primary outcome was met with a significantly shorter time until resolution of diarrhea in the treatment compared to the control group (median 3 vs. 4 days, log-rank test *p* < 0.001) with a hazard ratio of 0.7363 (confidence interval 0.6491–0.8353) for resolution of diarrhea (=dependent variable). The corresponding Kaplan Meier curves are shown in Figure 2. At day 3 of treatment, significantly fewer patients in the SF68 group (50.2%) reported ongoing diarrhea compared to controls (59.2%, *p* = 0.002, intention-to-treat analysis).

**Figure 3:**
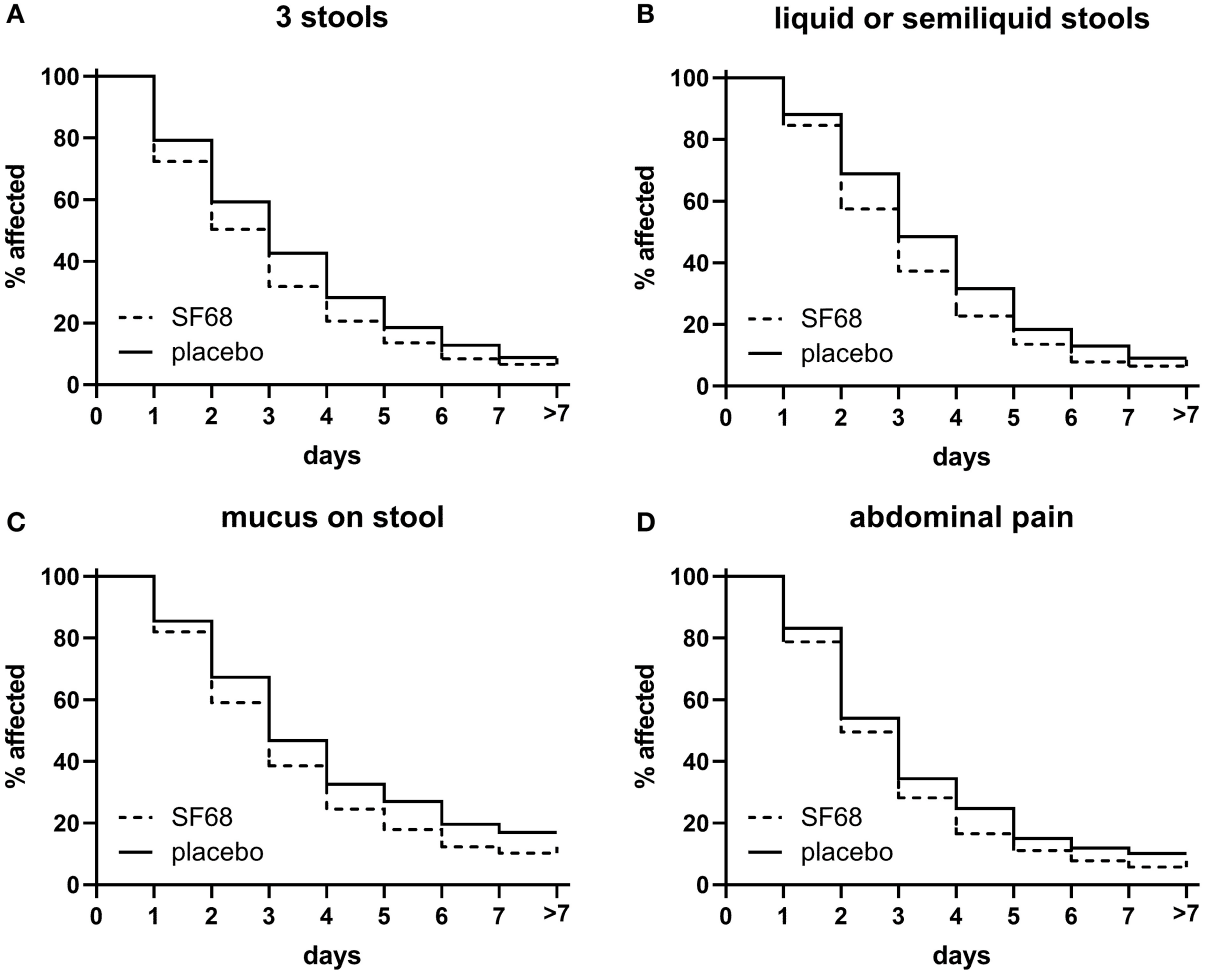
Time to resolution of secondary endpoints in the randomized-controlled treatment trial. **(A)** ≥3 stools [log-rank test *p* < 0.001 for SF68 vs. placebo; hazard ratio 0.8348 (confidence interval 0.7380–0.9443)], **(B)** liquid or semiliquid stools [log-rank test *p* < 0.001 for SF68 vs. placebo, hazard ratio 0.8200 (0.7247–0.9277)], **(C)** mucus on stool [log-rank test *p* = 0.019 for SF68 vs. placebo, hazard ratio 0.8073 (0.6553–0.9946)], and **(D)** abdominal pain [log-rank test *p* = 0.011 for SF68 vs. placebo, hazard ratio 0.8536 (0.7358–0.9904)].

Time to resolution of other symptoms (≥3 stools per day, presence of liquid or semi-liquid stools, mucus on stools, and abdominal pain) was also significantly reduced in the SF68 group compared to controls (Figure 3).

### Open-Label Treatment Study

Five thousand ninety-three outpatient subjects were enrolled in the open-label treatment study. Compared to the RCT, included patients were younger and more frequently reported food-borne illness and traveler‘s diarrhea as possible underlying cause. Disease severity at baseline was comparable to the RCT. However, abdominal pain and bloating were more frequently reported (Table 1). Primary and secondary outcomes were comparable to those seen in the RCT treatment group (Supplementary Figure 2).

### Randomized Prevention Study

One thousand three hundred ninety-seven patients were randomized 1:1 into the control group (700 subjects) and the SF68 arm (697 subjects). Baseline demographics and disease characteristics were comparable between the two groups (Table 2). All patients were enrolled by hospital-based physicians, 9.1% of patients were subsequently followed as outpatients. Patients were mainly treated for respiratory and urogenital infections (61.0 and 17.8%, respectively). The most commonly prescribed antibiotics were: cephalosporins (35.4%), ampicillin/amoxicillin (26.1%), and aminoglycosides (11.3%). In more than two thirds, antibiotics were administered intravenously. 662 (placebo group) and 661 patients (SF68 arm) completed the seven-day treatment course (Figure 1).

**Table 2:** Baseline demographics and disease characteristics for the prevention studies.

		**Randomized**			**Open-label**
		**Placebo**	**SF68**	**total**	
Gender	Male	395 (56.4)	398 (57.1)	793 (56.8)	2,323 (49.8)
	Female	305 (43.6)	299 (42.9)	604 (43.2)	2,342 (50.2)
Age	<14 years	59 (8.4)	61 (8.8)	120 (8.6)	588 (12.6)
	14–20 years	29 (4.1)	19 (2.7)	48 (3.4)	415 (8.9)
	21–40 years	99 (14.1)	104 (14.9)	203 (14.5)	1,437 (30.8)
	41–60 years	142 (20.3)	166 (23.8)	308 (20.2)	1,255 (26.9)
	61–70 years	141 (20.1)	115 (16.5)	256 (18.3)	555 (11.9)
	>70 years	230 (32.9)	232 (33.3)	462 (33.1)	415 (8.9)
Treatment setting	Hospital	636 (90.9)	634 (91.0)	1,270 (90.9)	-
	Outpatient	64 (9.1)	63 (9.0)	127 (9.1)	4,665 (100.0)
Type of infection	Respiratory	431 (61.6)	421 (60.4)	852 (61.0)	2,558 (54.8)
	Urological	122 (17.4)	127 (18.2)	249 (17.8)	1,189 (25.5)
	Other	147 (21.0)	149 (21.4)	296 (21.2)	918 (19.7)
Antibiotics	Cephalosporin	255 (36.4)	239 (34.3)	494 (35.4)	1,045 (22.4)
	Ampi- or amoxicillin	183 (26.1)	182 (26.1)	365 (26.1)	1,227 (26.3)
	Aminoglycoside	76 (10.9)	82 (11.8)	158 (11.3)	359 (7.7)
	Cotrimoxazole	42 (6.0)	53 (7.6)	95 (6.8)	611 (13.1)
	Penicillin	44 (6.3)	31 (4.4)	75 (5.4)	313 (6.7)
	Erythromycin	28 (4.0)	27 (3.9)	55 (3.9)	438 (9.4)
	Clindomyin/Lincomycin	10 (1.4)	5 (0.7)	15 (1.1)	229 (4.9)
	Other	62 (8.9)	78 (11.2)	140 (10.0)	443 (9.5)
Dosing route	Parenteral	480 (68.6)	470 (67.4)	950 (68.0)	1,320 (28.3)
	Oral	220 (31.4)	227 (32.6)	447 (32.0)	3,345 (71.7)
Early discontinuation	Unrelated	33 (4.7)	35 (5.0)	68 (4.9)	279 (6.0)
	Tolerability	5 (0.7)	1 (0.1)	6 (0.4)	126 (2.5)

*Data are shown as number of patients and % of group in parentheses*.

The primary endpoint was met with significantly fewer patients developing diarrhea in the SF68 arm (8.6%) compared to the control group (16.2%, *p* < 0.001, Table 3, per protocol analysis). In the intention-to-treat analysis, these rates were 8.2 vs. 15.3% (*p* < 0.001). In patients developing diarrhea, longer lasting diarrhea (≥2 days) was less frequently observed in the SF68 vs. control group (68.4 vs. 86%, *p* = 0.008). However, there were no significant differences regarding severity and time to onset of diarrhea between the two groups (Table 3).

**Table 3:** Primary and secondary outcomes for the prevention studies in those patients having completed the 7-day treatment course (per-protocol analysis).

		**Randomized**			**Open-label**
		**Placebo**	**SF68**	***P*-value**	
Total *n*		662	661		4,340
Incidence diarrhea		107 (16.2)	57 (8.6)	<0.001	499 (11.5)
Severity grading	Light	66 (61.7)	44 (77.2)	n.s.	410 (82.2)
	Moderate	38 (35.5)	13 (22.8)		79 (15.8)
	Severe	3 (2.8)	0 (0.0)		10 (2.0)
Time to occurrence, days	1	13 (12.1)	7 (12.3)	n.s.	65 (13.0)
	2	43 (39.3)	19 (33.3)		179 (35.9)
	3	26 (24.3)	15 (26.3)		157 (31.5)
	4	16 (15.0)	11 (19.3)		60 (12.0)
	>4	10 (9.3)	5 (8.8)		38 (7.6)
Duration, days	1	15 (14.0)	18 (31.6.)	0.008^*^	109 (21.8)
	2	38 (35.5)	17 (29.8)		224 (44.9)
	>2	54 (50.5)	22 (38.6)		166 (33.3)
Complications	Glossitis	31 (4.7)	16 (2.4)	0.026	182 (3.9)
	Anal fissures	16 (2.4)	8 (1.2)	n.s.	70 (1.5)

*Data are number of patients and % of group in parentheses*.

* *1 day vs. more than 1 day*.

### Open-Label Prevention Study

Four thousand six hundred sixty-four outpatient subjects were enrolled in the open-label prevention study. As compared to the RCT, included patients were younger and more frequently reported urological infections as underlying disease. Cotrimoxazole, erythromycin and clindamycin were more often used, while use of cephalosporins and aminoglycosides was less frequent, mostly due to fewer intravenous regimens (Table 2).

Diarrhea occurred in 11.5% of patients, which was mostly of light severity and short duration. The incidence was higher compared to SF68 arm in the RCT, but lower compared to the placebo group (*p* = 0.029 and *p* = 0.001). Glossitis and anal fissures occurred with a frequency of 3.9 and 1.5%. For details see Table 3.

### Adverse Events and Discontinuations

The incidence of adverse events was low in the two RCTs (1.1% for the treatment study and 1.4% for the prevention study). Reported events were gastric complaints, abdominal pain, vomiting and pruritus for the treatment study, and nausea, abdominal pain, vomiting, pruritis and bloating for the prevention trial. Adverse events were not more frequently observed in the SF68 arm compared to controls (Table 4). The rates of adverse events were higher in the open-label studies (4.7 and 7.4%, respectively). However, only 6.3 and 13.4% of these adverse events were considered to be SF68-related.

**Table 4:** Occurrence of adverse events (AEs) among all four studies.

	**Treatment studies**			**Prevention studies**		
	**Double-blind**		**Open**	**Double-blind**		**Open**
	**placebo**	**SF68**	**SF68**	**placebo**	**SF68**	**SF68**
Overall incidence	8/570 (1.4%)	6/564 (1.1%)	238/5,093 (4.7%)	14/700 (2.0%)	10/697 (1.4%)	344/4,665 (7.4%)
Nausea	-	-	63 (1.2%)	2	2	71 (1.5%)
Gastric complaint	1	3	45 (0.9%)	3	-	75 (1.6%)
Headache	1	-	41 (0.8%)	-	-	43 (0.9%)
Abdominal pain	2	1	38 (0.7%)	2	1	58 (1.2%)
Vomiting	1	1	34 (0.7%)	3	1	33 (0.7%)
Exanthema or pruritus	2	1	15 (0.3%)	1	2	30 (0.6%)
Constipation	1	-	2 (<0.1%)	-	-	-
Meteorism	-	-	-	2	4	34 (0.7%)
Joint pain	-	-	-	1	-	-

*Data are shown as number of patients and percent of group in parentheses*.

For the treatment RCT, early discontinuation of treatment was observed in 112 patients in the placebo group (19.6%) and in 119 patients in the SF68 arm (21.1%, n.s.). Treatment discontinuation due to early cure was more frequent in SF68-treated patients (16.8 vs. 13.3%). However, this did not reach statistical significance (*p* =0.099). Discontinuation due to intolerability was not significantly more often in one or the other group (0.4 vs. 1.1%, n.s., Figure 1). Treatment discontinuation for tolerability reasons in the open-label treatment study was similar as in the SF68 arm of the controlled study (0.3 vs. 0.4%, n.s.). For the prevention RCT, early discontinuation of prevention was seen in 38 patients in the placebo group (5.4%) and in 36 patients in the SF68 arm (5.2%, n.s.). Discontinuation due to intolerability was more frequent in the placebo group (0.7 vs. 0.1%). However, this difference did not reach statistical significance (*p* = 0.103). Treatment discontinuation for tolerability reasons was more frequently observed in the open-label prevention study (2.5%).

## Discussion

This was an analysis of two RCTs evaluating efficacy and safety of *Enterococcus faecium* SF68 in the treatment of acute diarrhea and prevention of antibiotic-associated diarrhea, and their corresponding open-label studies. Both RCTs were positive studies for the primary endpoint of a decrease in diarrhea duration and a reduction in occurrence of new diarrhea cases, respectively. SF68 was safe and well-tolerated.

With a total number of 6,236 patients included in the treatment trials and 5,737 subjects in the prevention studies, this represents by far the largest data set on SF68, actually accounting for more than half of all patients ever included in clinical trials with SF68. To the best of our knowledge, it also represents the largest dataset ever reported for any probiotic in the treatment or prevention of acute diarrhea (11). While these data were reported in abstract form in 1986 (19), it remains unclear why these data have not been reported as full article in the past. Given the fact that these are the largest trials with SF68 (and probiotics) in terms of treatment and prevention of acute diarrhea, and considering the possible impact of these data on clinical practice, we think it is our duty as caregivers to report these findings. However, our studies have one clear and major limitation: all trials were performed in Italy in the 1980s. So, (i) there was no institutional review board established at the leading study center at that time (University of Milan) as by then a legally binding obligation to obtain ethical committee approval had not existed yet; (ii) European GCP guidelines had not been published, (iii) apparently no registration on clinicaltrials.gov was done; and (iv) raw data was not accessible anymore despite intensive efforts supported by the company holding marketing authorization. More than 30 years after completion of the studies, they probably no longer exist as the obligation to preserve records has been time-barred in the meantime. Nonetheless, the studies adhered to all rules and regulations applicable in Italy at that time, the manuscript is based on a thorough study report prepared by Gipharmex, which was submitted to regulatory authorities in several countries, and independent statistical re-analyses were performed where applicable. For full transparency, the study report can be found in Supplementary Datasheet 1.

Considering all these limitations, a seven-day course of *Enterococcus faecium* SF68 was efficacious in the treatment of acute diarrhea. The rate of patients with ongoing diarrhea for three or more days was significantly reduced from 59.2 to 50.2%, and median duration of diarrhea decreased from 4 to 3 days. The results from the open-label study are in line with these findings. The total number of patients (*n* = 6,236) and the placebo-controlled, double-blind study design strengthen the so far limited evidence for SF68‘s use in the treatment of acute diarrhea (9). SF68 probably acts by restoring the protective function carried out by intestinal flora against pathogens. In clinical practice, shorter disease duration might have the following considerable impact: (i) less unnecessary treatment escalations (such as antibiotics for not improving symptoms); (ii) shorter absence from work and school; (iii) and probably lower health-care costs due to shorter hospitalizations. However, this has to be proven and our findings cannot be extrapolated to acute diarrhea in general, since bacterial infections with Salmonella as well as severely ill patients were excluded. At least in the latter, other treatment modalities appear to be more adequate than restoration of intestinal flora, although probiotics may have an add-on effect.

The prevention studies allowed including patients with a wide spectrum of types and doses of antibiotics. This can be seen as a limitation, but also as a benefit of the prevention studies. It is a limitation because the resulting cohorts are heterogenous, which makes crisp interpretation of the data more difficult. On the other hand, this is beneficial because it results in applicability of the findings to a wide spectrum of antibiotic treatments.

*Enterococcus faecium* SF68 prevented development of antibiotic-associated diarrhea. Significantly fewer patients reported diarrhea under antibiotic treatment when they received prevention with the probiotic drug (8.6%) compared to placebo (16.2%). The findings of the open-label study are accordant. The placebo rate of 16.2% goes in line with previous studies and meta-analyses showing occurrence of diarrhea with antibiotic treatment in about 20% (21). So, antibiotic-associated diarrhea is a problem and can be found in a non-negligible proportion of patients. The relative reduction of antibiotic-associated diarrhea by almost 50% (with an estimated number needed to treat of 13) is in accordance with a recent Cochrane review showing a decrease from 19 to 8% with a mixed group of probiotic treatments (21). For *Enterococcus faecium* SF68, our trial presents, by far, the largest study demonstrating its efficacy in the prevention of antibiotic-associated diarrhea. Of note, probiotic preventive treatment has its merits even in those patients developing diarrhea, since diarrhea appears to be significantly shorter compared to what is seen in placebo-treated patients. Since no major advances in regard to antibiotic treatment have been made in the last two decades, our data are of considerable value despite the fact that the trial was conducted back in the 1980s.

*Enterococcus faecium* SF68 was safe and well-tolerated. Assessment of overall tolerability showed that adverse events are very infrequent. In fact, most of the reported adverse events represent typical symptoms of acute enteritis or known side-effects of antibiotic treatment. So, even fewer side-effects were actually related directly to the SF68 treatment. Rate of adverse events was not higher compared to placebo.

In conclusion, the probiotic *Enterococcus faecium* SF68 is effective in the treatment of acute diarrhea and prevention of antibiotic-associated diarrhea. SF68 is generally well-tolerated and shows an excellent safety profile. Whether these conclusions also apply to other strains of *Enterococcus faecium* remains to be investigated.

## Data Availability Statement

All datasets generated for this study are included in the article/Supplementary Material.

## Ethics Statement

Ethical review and approval was not required for the study on human participants in accordance with the local legislation and institutional requirements. Written informed consent to participate in this study was provided by the patient or the participants' legal guardian/next of kin.

## Author Contributions

TG, MM, DT, HW, and SV: study concept and design. DT and HW: acquisition of data. TG, MM, DT, HW, and SV: analysis and interpretation of data. TG and MM: drafting of manuscript. DT, HW, and SV: critical revision of the manuscript for important intellectual content. TG, MM, and SV: supervision. All authors contributed to the article and approved the submitted version.

## Conflict of Interest

TG has a consulting contract with Sanofi-Aventis, received a travel grant from Falk Pharma GmbH and Vifor, and an unrestricted research grant from Novartis. MM was a former (2011–2016) employee of Boehringer Ingelheim and presently a consultant for Sanofi-Aventis. DT and HW are current employees of Sanofi-Aventis. SV has a consulting contract with Sanofi-Aventis and received consultant fees and unrestricted research grants from Vifor and Falk Pharma GmbH.

## Supplementary Material

Supplementary Datasheet 1 Study protocol (Gipharmex).

Supplementary Figure 2 Time to resolution of primary and secondary symptoms in the open-label treatment studies in comparison to the RCT.

## References

[1] Tam CC Rodrigues LC Viviani L Dodds JP Evans MR Hunter PR. Longitudinal study of infectious intestinal disease in the UK (IID2 study): incidence in the community and presenting to general practice. Gut. (2012) 61:69–77. 10.1136/gut.2011.238386 21708822 PMC3230829

[2] Lew JF Glass RI Gangarosa RE Cohen IP Bern C Moe CL. Diarrheal deaths in the United States, 1979 through 1987: a special problem for the elderly. JAMA. (1991) 265:3280–4. 10.1001/jama.1991.03460240076031 2046110

[3] Kosek M Bern C Guerrant RL. The global burden of diarrhoeal disease, as estimated from studies published between 1992 and 2000. Bull World Health Organ. (2003) 81:197–204. 10.1590/S0042-96862003000300010 12764516 PMC2572419

[4] Roberts JA Cumberland P Sockett PN Wheeler J Rodrigues LC Sethi D. The study of infectious intestinal disease in England: socio-economic impact. Epidemiol Infect. (2003) 130:1–11. 10.1017/S0950268802007690 12613740 PMC2869933

[5] Edelstein M Merk H Deogan C Carnahan A Wallensten A. Quantifying the incidence and cost of acute gastrointestinal illness in Sweden, 2013–2014. Epidemiol Infect. (2016) 144:2831–9. 10.1017/S0950268816000467 26964750 PMC9150422

[6] Szajewska H Skórka A. *Saccharomyces Boulardii* for treating acute gastroenteritis in children: updated meta-analysis of randomized controlled trials. Aliment Pharmacol Ther. (2009) 30:960–1. 10.1111/j.1365-2036.2009.04113.x 19807726

[7] Szajewska H Skórka A Ruszczynski M Gieruszczak-Białek D. Meta-analysis: *Lactobacillus* GG for treating acute gastroenteritis in children – updated analysis of randomised controlled trials. Aliment Pharmacsol Ther. (2013) 38:467–76. 10.1111/apt.12403 23841880

[8] Guarino A Ashkenazi S Gendrel D lo Vecchio A Sharmir R Szajewska H European society for pediatric gastroenterology, hepatology, and nutrition/european society for pediatric infectious diseases evidence-based guidelines for the management of acute gastroenteritis in children in Europe. J Pediatr Gastroenterol Nutr. (2014) 59:132–52. 10.1097/MPG.0000000000000375 24739189

[9] Szajewska H Guarino A Hojsak I Indrio F Kolacek S Shamir R. Use of probiotics for management of acute gastroenteritis. J Pediatr Gastroenterol Nutr. (2014) 58:531–9. 10.1097/MPG.0000000000000320 24614141

[10] Szajewska H Urbanska M Chmielewska A Weizman Z Shamir R. Meta-analysis: *Lactobacillus Reuteri* strain DSM 17938 (and the original strain ATCC 55730) for treating acute gastroenteritis in children. Benef Microbes. (2014) 5:285–93. 10.3920/BM2013.0056 24463209

[11] Allen SJ Martinez EG Gregorio GV Dans LF. Probiotics for treating infectious diarrhoea. Cochrane Database Syst Rev. (2010) 2010:CD003048. 10.1002/14651858.CD003048.pub3 21069673 PMC6532699

[12] Bellomo G Finocchiaro C Frigerio G New prospects in the treatment of enteritides in paediatrics. Méd et Hyg. (1979) 37:3781–4.

[13] Bellomo G Mangiagli A Nicastro L Frigerio G A controlled double-blind study of SF 68 strain as a new biological preparation for the treatment of diarrhoea in pediatrics. Curr Ther Res. (1980) 28:927–36.

[14] Camarri E Belvisi A Guidoni G Marini G Frigerio G. A double-blind comparison of two different treatments for acute enteritis in adults. Chemotherapy. (1981) 27:466–70. 10.1159/000238017 7028412

[15] Alvisi V Tralli M Loponte A Pavani F Massari M Double-blind study of treatment with SF 68 or with antibiotics in acute enteritis in adults. Clin Ther. (1982) 101:581–6. 6751663

[16] Mitra AK Rabbani GH. A double-blind, controlled trial of bioflorin (*Streptococcus Faecium* SF68) in adults with acute diarrhea due to *Vibrio* cholerae and enterotoxigenic *Escherichia Coli*. Gastroenterology. (1990) 99:1149–52. 10.1016/0016-5085(90)90638-H 2203662

[17] Lund B Edlund C. Probiotic *Enterococcus faecium* strain is a possible recipient of the vanA gene cluster. Clin Infect Dis. (2001) 32:1384–5. 10.1086/319994 11303279

[18] Holzapfel W Arini A Aeschbacher M Coppolecchia R Pot B. *Enterococcus faecium* SF68 as a model for efficacy and safety evaluation of pharmaceutical probiotics. Benef Microbes. (2018) 9:375–88. 10.3920/BM2017.0148 29633645

[19] Frigerio G A lactic acid producer enterococcus in the prevention of antibiotic-associated diarrhea and in the treatment of acute diarrheal disorders: a double-blind multicenter placebo-controlled clinical trial. Dig Dis Sci. (1986) 31(10 Suppl):496S.

[20] Lamberti LM Fischer Walker CL Black RE. Systematic review of diarrhea duration and severity in children and adults in low- and middle-income countries. BMC Public Health. (2012) 12:276. 10.1186/1471-2458-12-276 22480268 PMC3364857

[21] Goldenberg JZ Lytvyn L Steurich J Parkin P Mahant S Johnston BC Probiotics for the prevention of pediatric antibiotic-associated diarrhea. Cochrane Database Syst Rev. (2015) CD004827. 10.1002/14651858.CD004827.pub4 26695080

