# Supplementary material for: Randomized, Placebo-Controlled, Double-Blind and Open-Label Studies in the Treatment and Prevention of Acute Diarrhea With Enterococcus faecium SF68
Source: Front Med (Lausanne). 2020 Jun 19;7:276. doi: 10.3389/fmed.2020.00276 (PMC7326027; doi:10.3389/fmed.2020.00276)
Supplement: Supplementary Datasheet 1 — Study protocol (Gipharmex). [file Data_Sheet_1.PDF]

ENGLISH TRANSLATION

CONTROLLED DOUBLE - BLIND AND OPEN STUDY

MULTICENTRIC RESEARCH ON LACTOPRODUCER ENTEROCOCCUS SF68:

PREVENTIVE ACTIVITY IN ANTIBIOTIC-ASSOCIATED DIARRHEA AND THERAPEUTIC EFFICACY IN ACUTE ENTERITIS

Supplement at N. 8 of Kole
Continuing Education in Gastroenterology
Periodic of Gipharmex S.p.A. – Via P. Palagi 2, 20129 Milan
Registration of the Milan Court N. 467 of 6.12.1982

MULTICENTRIC CLINICAL INVESTIGATION ON BIOFLORIN

PRESENTATION

The Research and Development Unit of Gipharmex S.p.A. is proud to submit the results of this cooperative multicentric clinical investigation. The immediate publication of the results with the names of all the participating authors of the research, thus making possible the investigation, is designed to fulfill our commitment to all those physicians who have adhered to the project.

More than 1000 participating Italian physicians, more than 12.000 patients monitored throughout Italy, the methods adopted, corresponding to the most modern standards of the controlled clinical trial, are all elements of legitimate satisfaction for an initiative that, apart from the clinical results achieved, testifies to the possibility of realizing multidisciplinary approaches useful in assessing and defining the clinical-therapeutic properties of new drugs in the phase of enlarged use (the so-called phase 4) with particular reference to the current prescriptive use.

The results broadly confirm the practical utility, in terms of optimal tolerability and documented efficacy, even in clinical forms largely self-limiting, of the biological preparation consisting of the lactobacterium SF68 (BIOFLORIN) both in the treatment of enteritis and other acute forms of diarrhea as well as in the prevention of the most common reactions to antibiotics. These results are in line with those demonstrated in clinical research made in the early stages, but their importance derives from the fact that they now refer to case studies of much larger dimensions and relevancy to the commonly found disease, with notable correlation found by observers.

The aims of the researches and the structuring of the procedures did not provide specific detection on the nature and characteristics of the clinical forms to be studied, this being possible only in mono or paucicentric researches of intensive-type. The possible conclusions in this regard are therefore limited; however some aspects of a general nature have been well highlighted (for example, the high frequency of such clinical forms along with the configuration of the spectrum of symptoms that accompanies them, the frequency of reactions depending on the type of antibiotics taken and on the route of administration, etc.) and these elements represent a significant find that go beyond the precise definition of the therapeutic classification of the biological approach in study.

The presentation of the report on the multicentric research cannot be closed without thanking all those who have made feasible the realization of the initiative: to Prof. Mauro Moroni, for his valuable support and precious suggestions, to the Research and Development Unit of the Gipharmex group for setting up and processing the initiative, to the networks of external collaborators of Gipharmex and Bioresearch-Line RK for the intelligent methods with which they linked together the Clinical Centers and, at last, to all the Italian physicians who joined and supported the initiative with enthusiasm, enabling it to become a solid reality, thanks to their professional competence.

GIPHARMEX S.p.A.
Research and Development Unit

Hospital and regional physicians
co-authors of the multicentric research
in collaboration with the Research and Development Unit
of the Gipharmex group

MEDICI OPERANTI IN CENTRI OSPEDALIERI

AFFINI R.	SUZZARA (MN)	BARALDI B.	SERIATE (BG)
AGRICOLA R.	LANZO (TO)	BARASSI V.	LEGNAGO (VR)
AIELLO P.	SUBIACO (RM)	BARBERO M.	SAVONA
ALBANESE R.	SACILE (PN)	BARBUIANI M.	ADRIA (RO)
ALFANO G.	PAGANI (SA)	BARDELLI G.	MOLINELLA (BO)
ALLEGRI P.	GENOVA	BARDINI N.	CECINA (LI)
ALOIA V.	ACQUAVIVA (BA)	BARLOTTA A.	TORINO
ALTICHIERI M.	BUSSOLENGO (VR)	BARONI G.C.	MILANO
ANDREOLETTI M.	CERNUSCO S/N (MI)	BARSANTI G.	LUCCA
ANGELI L.	LUCCA	BARTOLINI F.	BARGA (LU)
ANGIOLI D.	AREZZO	BELLODI G.	GUASTALLA (RE)
ANNUNZIATA N.	MATERA	BELLUOMINI R.	LUCCA
ARANCI E.	ASTI	BELMONTE A.	COSENZA
ARAQUISTAIN G.	ROMA	BENEVENTI W.	RIVAROLO (TO)
ARENA G.	PALMI (RC)	BENFENATI F.	MOLINELLA (BO)
ARIANO R.	BORDIGHERA (IM)	BENZI D.	CREMA (CR)
AUZZANI A.	CREMONA	BERGONZI F.	TREVIGLIO (BG)
BAFFELLI E.	BRENO (BS)	BERNACCHI G.	CASTELLAMARE DI STABIA (NA)
BAGLIETTO B.	CARRARA (MS)	BERNINI G.	GUASTALLA (RE)
BAIONE M.T.	CAVA DEI TIRRENI (SA)	BERTINI M.	PISA
BAITA A.	VIMERCATE (MI)	BERTOLETTI A.	CREMONA
BALBASSARINI M.	ROMA	BERTOLUCCI P.	LUCCA
BALDASSARRE G.	ACQUAVIVA (BA)	BERTON A.	PIETRALIGURE (SV)
BALESTRA B.	RIVAROLO (TO)	BESOZZI F.	GAVARDO (BS)
BALLARDINI G.	TREVIGLIO (BG)	BETTATI G.	S. SECONDO PARMENSE (PR)
BALLINI A.	TREVIGLIO (BG)	BETTINI R.	IMOLA (BO)
BANDIERA G.	GENOVA VOLTRI (GE)	BIANCHI G.	CREMONA
BANDINO C.	S. GAVINO (CA)	BIANCHI L.	PONTREMOLI (MS)

BICCIATO L.	CITTADELLA (PD)	CARBONE D.	ROMA
BINELLI F.	PIACENZA	CARBONE G.	MONTEBELLUNA (TV)
BINI R.	LA SPEZIA	CARLINO G.	CASSINO (FR)
BNA' B.	MANTOVA	CAROLI A.	MONTEBELLUNA (TV)
BODINI P.	CREMONA	CASADEI A.	FORLI'
BOGNI M.E.	VERCELLI	CASALI R.	ROMA
BOI R.	CAGLIARI	CASARIN P.	SACILE (PN)
BONCINELLI L.	COMO	CASSANO F.	MELITO PORTO SALVO (RC)
BONDI G.	FORLI'	CASTELLUCCIO A.	SANREMO (IM)
BONFIGLIO M.	IMOLA (BO)	CAUSI G.	ADRIA (RO)
BONI M.	CORREGGIO (RE)	CAVALLI C.	PIACENZA
BRANZ F.	TRENTO	CAVASSINI G.B.	MOLINELLA (BO)
BROGI G.	CASTELFIORENTINO (FI)	CAVAZZUTI F.	ROVIGO
BRUNATI S.	MAGENTA (MI)	CAZZADORIA A.	VERONA
BURLANDO C.	SAMPIERDARENA (GE)	CECCHETTO G.	MONTEBELLUNA (TV)
BUSATTO L.	NOALE (VE)	CELLINI A.	SACILE (PN)
BUSCARINI L.	PIACENZA	CESARI F.	BERGAMO
CADEDDU P.L.	IERZU (NU)	CHASSEUR R.	AOSTA
CALABRESE G.	REGGIO CALABRIA	CHELLA S.	PISA
CALCAGNO G.	SAVONA	CHIA S.	S. GAVINO (CA)
CALIA P.	PISTOIA	CHINELLO L.	DOLO (VE)
CALIARI S.	ROVIGO	CIANCONI G.	OSTIA (RM)
CALLURA G.	BRESCIA	CINQUE N.	PISTOIA
CALTABIANO S.	ORBETELLO (GR)	CIRAOLO F.	MILAZZO (ME)
CANCELLIERI C.	FORLI'	COCCO F.	EMPOLI (FI)
CANE' G.	LOIANO (BO)	COINU M.	CAGLIARI
CANESCHI F.	AREZZO	COLETTI C.	CASSINO (FR)
CANI E.	IMOLA (BO)	COLOMBINI V.	ROMA
CANNISTRACI F.	MILAZZO (ME)	COLOMBO E.	VIMERCATE (MI)
CANNISTRACI G.	MILAZZO (ME)	COLOMBO M.	CERNUSCO S/N (MI)

COMASCHI M.	GENOVA VOLTRI (GE)	DE LEO A.	PAGANI (SA)
CONTI M.	BRENO (BS)	DE LIPSIS E.	ROMA
CONZ P.A.	CITTADELLA (PD)	DE MAESTRI C.	CREMONA
COPPOLA C.	SUZZARA (MN)	DE MICHELI E.	MODENA
CORBELLINI A.	RIVOLTA D'ADDA (CR)	DE PASQUALE F.	MESSINA
CORSINI P.	GROSSETO	DE PRA' M.	CARRARA (MS)
CORTINI C.	FORLIMPOPOLI (FO)	DE STEFANO A.	LOIANO (BO)
CRAVERO F.	ALBA (CN)	DEL CORSO L.	PISA
CRICONIA A.M.	ROMA	DEL PERO B.	BOLZANO
CRINO' B.	LOIANO (BO)	DEL PIANO R.	NOVARA
CUCINELLI M.L.	AREZZO	DEL POGGIO P.	TREVIGLIO (BG)
CURIONI R.	MAGENTA (MI)	DELL'AQUILA A.M.	CASERTA
CUSCINI P.	MOLINELLA (BO)	DELLA CASA R.	SALERNO
CUSTURONE D.	PALMI (RC)	DELLA MARCHINA M.	RIMINI (FO)
D'ALESSIO G.	BENEVENTO	DELLA SALA A.	PAGANI (SA)
D'AMICO N.	CATANZARO	DELLA SANTA M.	PISA
D'ANGELO A.	DOLO (VE)	DESIDERI M.	LATISANA (UD)
D'ANGELO T.	PISA	DESTE A.	CREMA (CR)
D'ANNEO G.	REGGIO CALABRIA	DEVIZZI G.	VIZZOLO PREDABISSI (MI)
D'ARCO A.	PONTREMOLI (MS)	DI BARTOLO F.	CECINA (LI)
D'IGNAZIO F.	ROMA	DI GIANDOMENICO G.	BOLOGNA
DAL PANE M.	FORLIMPOPOLI (FO)	DI NOLFO M.	SERIATE (BG)
DALAI P.	BUSSOLENGO (VR)	DI SALVIO E.	PAGANI (SA)
DALLA VEDOVA F.	BUSSOLENGO (VR)	DIANA A.	SAVIGLIANO (CN)
DANESE G.	ROMA	DRAGO T.	CAMOGLI (GE)
DANTE G.L.	PIETRALIGURE (SV)	DREI F.	BOLZANO
DE ANGELIS S.	SEZZE (LT)	ERVO R.	ALBENGA (SV)
DE CARLO M.	GENOVA-SESTRI PONENTE (GE)	EVANGELISTI G.	IMOLA (BO)
DE CIUTIIS C.	CASERTA	FAGGIOLI M.	FERRARA
DE DONA' D.	RAVENNA	FAGLIA E.	MILANO

FALSETTI F.	BRESCIA	GANDOLFI E.	REGGIO CALABRIA
FAMILIARI F.	MELITO PORTO SALVO (RC)	GANGA E.	CAGLIARI
FARIELLO V.	CAVA DEI TIRRENI (SA)	GANGA R.	CAGLIARI
FAVALES E.	MILANO	GARAGNANI A.	IMOLA (BO)
FAVARATI G.	LEGNAGO (VR)	GARDA E.	TORINO
FEDERICI G.	PISA	GASPARONI A.	CONEGLIANO (TV)
FEDERIGHI G.	PISTOIA	GEMINIANI G.D.	LUGO (RA)
FENINI R.	TREVIGLIO (BG)	GENTILE E.	ADRIA (RO)
FERLINI A.	FAENZA (RA)	GIACCA S.	BORDIGHERA (IM)
FERRACIN G.	VICENZA	GIANNINI R.	SAVONA
FERRARESE M.	ALESSANDRIA	GIORCELLI W.	VERCELLI
FERRARI F.	CREMONA	GIRONDA G.B.	VILLAFRANCA (VR)
FERRARINI F.	MANTOVA	GITTO F.	BARCELLONA P.G. (ME)
FERRARO F.	MELITO PORTO SALVO (RC)	GOGLINO A.	ALESSANDRIA
FERROTTI A.	OSTIA (RM)	GOLLINI C.	CORREGGIO (RE)
FICHERA S.	PALMANOVA (UD)	GRAGNANI G.	CECINA (LI)
FIORENTINI F.	TRENTO	GRAMELLINI F.	FORLIMPOPOLI (FO)
FOCA' D.	CAVA DEI TIRRENI (SA)	GRECO N.	CATANZARO
FONTANA F.	BARGA (LU)	GRILLANDA G.	CERNUSCO S/N (MI)
FONTANA G.	FORLI'	GRILLO A.	BOLOGNA
FORMANTICI F.	VIMERCATE (MI)	GRITTI F.	BOLOGNA
FORTE G.	CATANZARO	GUADAGNINI I.	CREMA (CR)
FOTI G.	SAVONA	GUADAGNINO V.	BENEVENTO
FRAGUGLIA C.	ALBENGA (SV)	GUIDERI R.	MASSA MARITTIMA (GR)
FRANCESCHINI A.	VOLTA MANTOVANA (MN)	HUSCHER C.	GAVARDO (BS)
FUSER R.	NOALE (VE)	IMPROTA L.	S. AGNELLO SORRENTO (NA)
GAGGERO A.	GENOVA VOLTRI (GE)	IOPPOLO V.	VICENZA
GAGGERO E.	GENOVA-SESTRI PONENTE (GE)	IOVINO G.	S. AGNELLO SORRENTO (NA)
GALANO L.	CAVA DEI TIRRENI (SA)	ISOLA I.	BORDIGHERA (IM)
GANASSINI A.	VERONA	LAI M.	IERZU (NU)

LANUCARA P.	REGGIO CALABRIA	MANICONE O.	MATERA
LANZAFAME A.	S. BONIFACIO (VR)	MANNO G.	COSENZA
LASCARO M.	MATERA	MANOPULO R.	BOLOGNA
LAUDANNA E.	CARRARA (MS)	MARALDI F.	FORLI'
LAURI G.	ROMA	MARCHESI M.	BOLZANO
LAURIA A.	REGGIO CALABRIA	MARCHETTINI G.	GROSSETO
LAZZA M.	VIZZOLO PREDABISSI (MI)	MARCHI M.	CORREGGIO (RE)
LEONARDI M.	SESTRI LEVANTE (GE)	MARENGO G.	MONCALIERI (TO)
LEONE C.	BORDIGHERA (IM)	MARIGO S.	LA SPEZIA
LEONE G.	VOLTA MANTOVANA (MN)	MARINO F.P.	CASERTA
LEPRE A.	CREMA (CR)	MARINO R.	VIZZOLO PREDABISSI (MI)
LIPPI MALDINI A.	LUGO (RA)	MARINONI F.	LEGNAGO (VR)
LITTA MODIGNANI R.	TREVIGLIO (BG)	MARINUCCI I.	FAENZA (RA)
LO CURZIO G.	MILAZZO (ME)	MARTINOGLIO P.	TORINO
LO NIGRO M.	MATERA	MASIELLO O.	LOIANO (BO)
LO PRESTI D.	PALMI (RC)	MASSUCCI M.	ORBETELLO (GR)
LOPES R.	SUBIACO (RM)	MATTI F.	VOGHERA (PV)
LORUSSO F.P.	CASERTA	MAXIA G.	S. GAVINO (CA)
LUPINACCI G.	CREMA (CR)	MAZZEO F.	BORGONOVO V.T. (PC)
LUPO S.	ALBENGA (SV)	MAZZINI V.	MOLINELLA (BO)
MADINI G.C.	CREMONA	MAZZUCA S.	CATANZARO
MAESTRINI M.	MASSA MARITTIMA (GR)	MELANDRI P.	LUGO (RA)
MAGNANINI G.	GUASTALLA (RE)	MELARAGNO P.	ROMA
MAIOLINO M.	MESSINA	MENGOLI R.	CORREGGIO (RE)
MAIORANA V.	MESSINA	MENNA G.	SALERNO
MALARA F.	MESSINA	MESCHI P.	BARGA (LU)
MALLOCCI R.	GHILARZA (OR)	MESSINA Q.	SACILE (PN)
MANAI M.	CAGLIARI	MIAN P.	CERNUSCO S/N (MI)
MANDANICI A.	BARCELLONA P.G. (ME)	MIGNANI E.	LA SPEZIA
MANICARDI V.	GUASTALLA (RE)	MILILLO V.	CONEGLIANO (TV)

MODENA G.	NOALE (VE)	PADULA F.	PECETTO TORINESE (TO)
MOLINARI A.	ALESSANDRIA	PAGANINI P.A.	RAPALLO (GE)
MONTANAR F.	PALMANOVA (UD)	PALADINI A.	PISA
MONTEVERDE A.	NOVARA	PALAGI E.	LUCCA
MONTI M.	BOLOGNA	PANI C.	VIMERCATE (MI)
MORELLI A.	MATERA	PANUCCIO D.	LOIANO (BO)
MORETTI G.B.	SERIATE (BG)	PARATORE A.	BARCELLONA P.G. (ME)
MORICCA N.	ROMA	PARELLO S.	ASTI
MORINI S.	PESCIA (PT)	PARENTE M.	CASSINO (FR)
MORONI M.	MILANO	PARRI A.	EMPOLI (FI)
MOTTA E.	CREMA (CR)	PASCARELLI E.	REGGIO CALABRIA
MURA E.	GHILARZA (OR)	PATELLANI M.	SESTRI LEVANTE (GE)
MURGIA B.	IERZU (NU)	PAVERO A.	RAPALLO (GE)
MURGIA M.	IERZU (NU)	PAZZAGLIA R.	CASTELNUOVO GARFAGNANA (LU)
NAPODANO C.	PAGANI (SA)	PAZZI P.	FERRARA
NEGRINI F.	BERGAMO	PEDACE G.C.	OSTIA (RM)
NEGRINI G.P.	BRESCIA	PEDATA A.	CASTELLAMARE DI STABIA (NA)
NERI D.	EMPOLI (FI)	PEDRINI O.	MOLINELLA (BO)
NICOLLI F.	RAVENNA	PELLEGATTI P.	CAGLIARI
NICROSINI F.	VOGHERA (PV)	PELLIZZERI F.	MESSINA
NIMIS R.	LATISANA (UD)	PEROZZIELLO F.	VIZZOLO PREDABISSI (MI)
NURRA P.	SAVONA	PERRONI G.B.	MESSINA
OCCHIPINTI P.	NOVARA	PERSICO L.	ROMA
ODDI A.	GENOVA-NERVI (GE)	PESCE G.	ALESSANDRIA
OLIVETTI R.	VOLTA MANTOVANA (MN)	PETA G.	BOLOGNA
ORLANDINI D.	CORREGGIO (RE)	PETRACCA E.	ROVIGO
PACIFICO R.	SALERNO	PIANCINO G.	PECETTO TORINESE (TO)
PACINI F.	CASTELFIORENTINO (FI)	PICHI V.	AREZZO
PACINI R.	PISTOIA	PICOLLO G.	ALBA (CN)
PADULA A.	MATERA	PIERATTI G.	COMO

PIERUCCINI G.	BARGA (LU)
PIGHINI C.	PONTREMOLI (MS)
PILASTRI M.G.	CASTELFRANCO (TV)
PILLERI G.P.	CAGLIARI
PINNA G.	CAGLIARI
PINO G.	ALESSANDRIA
PITTSCHILLER K.	BOLZANO
POLIMENI F.	REGGIO CALABRIA
POMPEI A.	CAMOGLI (GE)
PORCEDDA R	S. GAVINO (CA)
PRISCO F.	CAVA DEI TIRRENI (SA)
PRISTERA R.	BOLZANO
PUCI R.	LANZO (TO)
QUATTROCCHI R.	MAGENTA (MI)
RAGAINI S.	TREVIGLIO (BG)
RANDONE G.	TREVIGLIO (BG)
RANIERI S.	RAVENNA
RAVAGNANI E.	MONCALIERI (TO)
RE L.	TREVIGLIO (BG)
REA C.	ROMA
RICCIARDI A.	CASERTA
ROLFO P.	TORINO
ROMANELLI R.	LOIANO (BO)
ROMANO P.M.	GENOVA-NERVI (GE)
ROMANO R.	GENOVA-NERVI (GE)
RONCARATI C.	FORLIMPOPOLI (FO)
ROSONE A.	SAVONA
ROSSI G.	AREZZO
ROSSI G.	GUASTALLA (RE)
ROSSI M.	MASSA MARITTIMA (GR)
ROSSI S.	PIACENZA
ROTTA E.	CORREGGIO (RE)
ROVESTI D.	SUZZARA (MN)
RUBINI F.	VERONA
RUBINI R.	LEGNAGO (VR)
RUSSO F.	SUBIACO (RM)
RUTILI A.	ORBETELLO (GR)
SABA P.	PESCIA (PT)
SACCO P.	GENOVA VOLTRI (GE)
SALONE G.	SEZZE (LT)
SALUCCO C.	EMPOLI (FI)
SANFILIPPO M.	PIETRALIGURE (SV)
SANNAZZARI P.	ALBENGA (SV)
SANTILLO G.	CASTELNUOVO GARFAGNANA (LU)
SARDEO G.	MONTEBELLUNA (TV)
SCALABRINO A.	PESCIA (PT)
SCASSO A.	PISA
SCHIARETTI L.	S. SECONDO PARMENSE (PR)
SCHIAVI L.A.	LOIANO (BO)
SCIALDONE A.	CASERTA
SCRINZI F.	S. BONIFACIO (VR)
SEBASTIANI P.	SAMPIERDARENA (GE)
SEROFILLI S.	RIMINI (FO)
SICCARDI L.	GHILARZA (OR)
SIGNORELLI S.	CORREGGIO (RE)
SOFIA M.	RIVOLTA D'ADDA (CR)
SOMENZI A.M.	CREMA (CR)
SOTTINI G.	BRESCIA
STABELLINI G.	FERRARA
STAITI A.	BARCELLONA P.G. (ME)

STOPPA L.	ADRIA (RO)	VACCARI G.L.	SUZZARA (MN)
TAFI A.	CASTELFIORENTINO (FI)	VALENTI M.T.	LEGNAGO (VR)
TANSINI P.	PIACENZA	VALMACHINO G.	CASTELFRANCO (TV)
TANZI G.	ROMA	VANNATI G.B.	RAPALLO (GE)
TASSARA A.	AOSTA	VARRICCHIO A.	ALESSANDRIA
TATA F.	SANPIERDARENA (GE)	VATTERONI M.	PISA
TEDESCO V.	PALMI (RC)	VENERI L.	GUASTALLA (RE)
TEDOLDI A.	PIETRALIGURE (SV)	VENEZIA L.	ACQUAVIVA (BA)
TESTA S.	SAVONA	VENTURA M.	PISA
TIBERI P.	SEZZE (LT)	VENTURELLI R.	RIVOLTA D'ADDA (CR)
TISCI R.	ACQUAVIVA (BA)	VENZANO C.	GENOVA-SESTRI PONENTE (GE)
TOGLIA M.	LANZO (TO)	VERNACI R.	REGGIO CALABRIA
TOMA G.	PONTREMOLI (MS)	VERZE' A.	VILLAFRANCA (VR)
TOMBA A.	NEGRAR (VR)	VEZZANI F.	REGGIO EMILIA
TORCIA L.	ROMA	VILARDO L.	COSENZA
TORRI F.	GAVARDO (BS)	VILLA R.	BERGAMO
TOSCANO A.	ROMA	VIVIANI G.	BRENO (BS)
TOSELLI P.	SAVIGLIANO (CN)	VOLPI A.	MONTEBELLUNA (TV)
TRABACCO M.	VIMERCATE (MI)	ZABBERONI W.	RAVENNA
TRAMALLONI G.L.	RIVOLTA D'ADDA (CR)	ZACCARELLI N.	FORLIMPOPOLI (FO)
TREVISANI L.	FERRARA	ZAMPINI A.M.	CREMA (CR)
TRIFILETTI F.	MESSINA	ZARRA R.	SEZZE (LT)
TRIMBOLI V.	COSENZA	ZAULI T.	FAENZA (RA)
TROVATO G.	MESSINA	ZENARI L.	NEGRAR (VR)
TROVELLO C.	CASERTA	ZENNARO T.	ROMA
TRUFFA G.	BRESCIA	ZINOLLI L.	SANPIERDARENA (GE)
TURCO D.	TORINO		

MEDICI DI TERRITORIO

ABATINI M.	ARICCIA (RM)	AZZAROLI C.	FORLI'
ALBANESE V.	MESSINA	BALDINI L.	NAPOLI
ALIBRANDO A.	MESSINA	BARANELLO G.	MIRABELLO SANNITICO (CB)
ALLASCIA B.	SAVIGLIANO (CN)	BARAVALLE A.	ALBA (CN)
ALU' A.	TRIESTE	BARDELLI A.	MILANO
ALUNNI M.	PERUGIA	BARONE G.	SIRACUSA
AMATO V.	MOLFETTA (BA)	BARTOLDI M.	MARTELLAGO (VE)
AMBROSIO A.	BASSANO (VI)	BASSO G.	GATTORNA (GE)
AMMENDOLA C.	BARRA (NA)	BASTAROLI E.	PAVIA
AMOROSO L.	SAN SEVERO (FG)	BATTAGLIA D.	PALERMO
ANDREINI R.	VECCHIANO (PI)	BATTAINI S.	MILANO
ANGELINI F.	NOVARA	BATTILORO G.	NAPOLI
ANGELINO A.	SANREMO (IM)	BATTISTI L.	MANTOVA
ANGONE G.	FASANO (BR)	BAVASTRELLI M.	ROMA
ANGUILLA G.	LECCE	BECCHI G.	REGGIO EMILIA
ANNALORO C.	MILANO	BEDINI L.	PISA
ANNUNZIATA G.	MATERA	BELLANCA N.	CALTANISETTA
ANTONIETTI A.	TREVISO	BELLIGOLI A.M.	SOMMACAMPAGNA (VR)
ANTONINI M.	BIELLA (VC)	BELLINGRERI V.	SOLBIATE ARNO (VA)
APPOLLONI L.S.	FABRIANO (AN)	BELLONI A.	LA SPEZIA
ARGENTO M.P.	NAPOLI	BELOTTI L.	BOLOGNA
ARMANI F.	UDINE	BELUTTI G.B.	SALE MARASINO (BS)
ARPAIA P.	COSENZA	BENINATO M.	VIDOR (TV)
ARTIBANI A.	MONDOLFO (PS)	BENSI A.	ASSISI (PG)
ARUTA N.	ARZANO (NA)	BENVENUTO M.	CESATE (MI)
ASCIONE F.	PORTICI (NA)	BERARDUCCI F.	SULMONA (AQ)
ATZORI I.	PORTOSCUSO (CA)	BERETTA U.	MONZA (MI)
AUTORINO D.	BARI	BERGAMASCHI G.C.	BERGAMO
AVELLINI M.	PERUGIA	BETTI P.	FORLI'

BEVACQUA P.	COSENZA	BUSNARDO F.	BASSANO (VI)
BEZZI R.	LA SPEZIA	CACACI V.	PEDASO (AP)
BIANCHI M.	ORZONOVO (SP)	CALCAGNI C.	ROMA
BIANCO G.	NAPOLI	CALCULLI N.	MATERA
BICINI M.	PERUGIA	CALDERONE G.	NAPOLI
BILANCIO P.	GRUMO NEVANO (NA)	CALDERONI A.	GODO (RA)
BIZZARRO G.	NAPOLI	CALICCHIA A.	ROMA
BOERCHI A.	MILANO	CALLAMUGGI G.	TORINO
BOMBIERI G.	SCORZE' (VE)	CAMA F.	MESSINA
BONGIOVANNI F.	BOLOGNA	CAMBILARGIU L.	ROMA
BONGIOVANNI I.	VARESE	CAMINO F.	MILANO
BONSIGNORE R.	NAPOLI	CAMPOBASSO G.	FOGGIA
BORGIA L.	LECCE	CANALI F.	REGGIO EMILIA
BORGIA T.	LECCE	CAPOLINO P.	STEZZANO (BG)
BORGOGNA A.	RANICA (BG)	CAPORELLO M.	LARIANO (RM)
BORIOLI A.	NOVARA	CAPPIO M.	BIELLA (VC)
BORRONI M.	SARONNO (VA)	CAPRI' G.	CATANIA
BOSI M.	REGGIO EMILIA	CAPUTO A.	LAMEZIA TERME (CZ)
BOTTAZZOLI A.	PISA	CARAVETTA P.	COSENZA
BOTTI M.	PIACENZA	CARELLA F.	S. REMO (IM)
BRATTOLI M.	MOLFETTA (BA)	CARELLI F.	MILANO
BREGLIA G.	SENISE (PZ)	CARELLO M.	MARANO (NA)
BRIZIO A.	NAPOLI	CARIDI G.	REGGIO CALABRIA
BRIZIO E.	FOSSANO (CN)	CASARTELLI G.	GIRONICO (CO)
BROUSSARD G.	TERNI	CASCIA S.	CASTELPLANIO STAZIONE (AN)
BRUNO V.	TARANTO	CASI F.	MILANO
BRUSCO E.	SALUZZO (CN)	CASSANDRO A.	NAPOLI
BUCCI M.	TREVISO	CASSARINI F.	BOLOGNA
BURLINI F.	VERONA	CASSETTA M.	TARANTO
BUSI A.	CESENATICO (FO)	CASTAGNINO G.	SIRACUSA
BUSIGNANI F.	ORIGGIO (VA)	CATALANO R.	CATANIA

CATANO C.	BROLO (ME)	CORCIONE M.	ROMA
CATTANEO G.	OSTIGLIA (MN)	CORDISCO E.	TERMOLI (CB)
CAVALLI M.	MARANO (NA)	CORDOVA D.	REGGIO CALABRIA
CAVANDOLI P.	REGGIO EMILIA	CORFINI A.	TREMIGNON DI PIAZZOLA (PD)
CAVAZZONI D.	BOLOGNA	CORGATELLI G.	VARESE
CAVONE M.	BARI	CORVINO P.	NAPOLI
CECCON M.A.	S. MARIA DELLE MOLE (RM)	COSCIA A.	NAPOLI
CEDRONE R.	CASALVIERI (FR)	COSTANTINI S.	ANCONA
CENZATO G.	NAPOLI	COSTANTINI S.	BOLOGNA
CERIANI A.	JERAGO (VA)	COTTANI A.	ROMA
CHIACCHIO G.	GRUMO NEVANO (NA)	CREMASCHI S.	VEZZANO (RE)
CHIARAMONTE T.	FALZE' DI PIAVE (TV)	CREMONINI G.P.	BOLOGNA
CHINNICI V.	CATENA NUOVA (EN)	CUCCHIARA P.	BOLZANO
CHIODAROLI A.	MILANO	CUCINOTTA A.	MESSINA
CIAININI G.	MILANO	CULOTTA P.	MILANO
CIGOLINI S.	ARQUATA (AL)	CURATOLA P.	SOVERATO (CZ)
CINONE M.	LUCITO (CB)	CUTTIN S.	MONZA
CIRILLO D.	GRUMO NEVANO (NA)	D'ALESSANDRO R.	COSENZA
CIRILLO M.P.	MILANO	D'ANDREA R.	CASAGIOVE (CE)
COCCA V.	CASORIA (NA)	D'ANGELA C.	BARI
COCCONI F.	ACQUANEGRA (MN)	D'ASCENZO G.	MONTENERO DI BISACCIA (CB)
COLAJANNI R.	MILANO	D'ERCOLE M.	MODUGNO (BA)
COLAMINE' V.	NAPOLI	D'IMPERIO F.	ROMA
COLANERI A.	BARANELLO (CB)	DALLA VIA D.	VICENZA
COMISSO P.L.	PALAZZOLO DELLO STELLA (UD)	DAMIANO S.	NAPOLI
CONIO P.	TAGGIA (IM)	DE ANDREIS B.	SAN REMO (IM)
CONTI A.	MONZA (MI)	DE BENEDITTIS M.	RONCHI DEI LEGIONARI (GO)
CONTI M.L.	BADALUCCO (IM)	DE CAPRIO M.	NAPOLI
CONTI S.	FALCONARA (AN)	DE CICCO R.	SERINO (AV)
CONTINI E.	SASSARI	DE DEO A.	SULMONA (AQ)
CORAZZA G.	GENOVA	DE FRANCESCO F.	MONTE MARANO (AV)

DE LEO V.	RUVO DI PUGLIA (BA)	DI SOMMA C.	MONTE SARCHIO (BN)
DE LISIO E.	ROMA	DI STEFANO O.	BRESCIA
DE LUCCHI P.	GENOVA	DIASPRO G.	NAPOLI
DE MARCO N.	CAMPOLIETO (CB)	DIBLAS R.	MONTEBELLUNA (TV)
DE MARZO P.	BARI	DIONESE S.	CONEGLIANO (TV)
DE MATTEIS P.	TERNI	DIOTALLEVI M.	FALCONARA (AN)
DE PAOLI F.	ROMENTINO (NO)	DONADELLI G.	DOSSO BUONO (VR)
DE ROSA E.	S. MARIA CAPUA VETERE (CE)	DONAZZAN A.	BASSANO (VI)
DE SILVA L.	AVELLINO	DUGGENTO G.	FALCONARA (AN)
DE TOMMASO C.	BARI	DURONIO P.	L'AQUILA
DE VECCHI E.	SERNAGLIA DELLA BATTAGLIA (TV)	DUVIA R.	COMO
DEL GROSSO B.	ROMA	ERMINI G.	BOLOGNA
DEL PRETE F.	GRUMO NEVANO (NA)	ESPOSITO F.	NAPOLI
DEL VESCOVO R.	S. MARIA DELLE MOLE (RM)	FABRETTI M.	ANCONA
DELFINI G.P.	CREMONA	FAGGIOLI F.	BOLOGNA
DELITALA A.	ORISTANO	FALATO M.R.	GUARDIA S. FRAMONDI (BN)
DELLA MARCA G.	PORTICI (NA)	FARINA A.	SANT'ARCANGELO (PZ)
DI BIASIO G.	PERUGIA	FARINA G.	ROMA
DI CARLO S.	BARI	FAZIO S.	S. ANGELO LIMOSANO (CB)
DI CARO S.	MESSINA	FEDRIGO PERISSUTTI F.	PERTEGADA DI LATISANA (UD)
DI GIOVINE E.	FOGGIA	FERIOLI M.	CASUMARO (FE)
DI GIROLAMO E.	TORELLA DEL SANNIO (CB)	FERONE F.	NAPOLI
DI GIUSEPPE A.	REGGIO CALABRIA	FERRANDO F.	CAVALICCO (UD)
DI GRANDI S.	CATANIA	FERRARI G.	CASCIAGO (VA)
DI MARIA M.A.	FIUMICINO (RM)	FERRARI M.	MILANO
DI MARTINO G.	PALERMO	FESTA A.	NAPOLI
DI MIGLIO G.	BRESCIA	FILACI A.	LAUREANA DI BORELLO (RC)
DI NUNNO C.	BOLOGNA	FILIERI A.	LECCE
DI PASQUALE M.G.	MENTANA TOR LUPARA (RM)	FILIPPI E.	CUNEO
DI PIETRO A.	OZZANO DELL'EMILIA (BO)	FIORENTINI L.	PADOVA
DI PINO L.	REGGIO CALABRIA	FIORLETTA M.	ROMA

FLAVONI A.	BOLOGNA	GESUALDI F.	LATRONICO (PZ)
FOCO G.	COSENZA	GHERMANDI E.	BOLOGNA
FOIS G.	ALGHERO (SS)	GHIRARDELLI G.	GENOVA
FONDERICO C.	OTTAVIANO (NA)	GIACULLI V.	BARI
FORCOLIN R.	VERZUOLO (CN)	GIANFREDA R.	VERONA
FORMENTI E.	BERGAMO	GIANNI G.	PISA
FORNASIR G.	CAERANO S. MARCO (TV)	GIANNUOLI D.	BARI
FRANGELLA V.	GIOIA TAURO (RC)	GIOFFRE M.G.	NAPOLI
FRAZZINGARO S.	LAZISE (VR)	GIOMBI M.	CERRETO D'ESI (AN)
FROIO S.	SOVERATO (CZ)	GIORDANO C.	ARBOREA (CA)
FULVIO P.	MILANO	GIROLAMI A.M.	MOROLO (FR)
GABRIEL G.	CAMPOBASSO	GIULIANO S.	SOMMA VESUVIANA (NA)
GAIONE M.	OVADA (AL)	GIUSTI M.	CATANZARO
GALAI M.	REMANZACCO (UD)	GLIELMO G.	BENEVENTO
GALAS E.	GRADISCA D'ISONZO (GO)	GONZI A.	MILANO
GALASSINI D.	PALAZZOLO DELLA STELLA (UD)	GRASSI A.	UDINE
GALDIERI V.	RIMINI (FO)	GRASSO A.	ACI S.ANTONIO (CT)
GALLICCHIO N.	TARANTO	GRASSO L.	ARIANO IRPINO (AV)
GALLIO F.	VENARIA (TO)	GRECO L.	VARESE
GANDOLFO G.	S. REMO (IM)	GREMIGNAI G.	PISA
GARAGNANI M.	BOLOGNA	GREPPI E.	BORGO D'ALE (VC)
GARDINI R.	AVELLINO	GRESSI E.	GORIZIA
GAROFANO I.	PIRRI (CA)	GRIECO V.A.	CASORIA (NA)
GASTALDO G.	GENOVA	GRILLOTTI E.	GENOVA-BOLZANETO
GATTI M.	FABRIANO (AN)	GRISTINA S.	ROMA
GATTULLI G.	RUVO (BA)	GROPPO D.	FOSSANO (CN)
GAVARINI O.	LA SPEZIA	GUARINO M.	AVELLINO
GENTILE C.	PALERMO	GUBIANI M.	UDINE
GENTILEZZA M.	VELLETRI (RM)	IANNESSI E.	L'AQUILA
GENTILI S.	NARNI (TR)	IEZZONI L.	BARI
GENTILINI A.	LAVIS (TN)	INDOLFI F.	AUSONIA (FR)

INDRIERI V.	PEDACE (CS)	LOPRETE P.	MILANO
INTINI G.	PUTIGNANO (BA)	LORENZONI L.	LAVIS (TN)
IORI G.	REGGIO EMILIA	LORUSSO O.	BARI
IORIO R.E.	CAPUA (CE)	LUGANI A.	GENOVA-CORNIGLIANO
IPPOLITI G.B.	PAVIA	LUPIERI S.	UDINE
ISOLA G.	GENOVA	LUSARDI M.	CASTELFIDARDO (AN)
JENNO G.	VENARIA (TO)	LUSZTIG I.	REGGIO EMILIA
JORIZZO V.G.	SCHIO (VI)	LUZZETTI G.	CIVITAVECCHIA (RM)
KOUVALIS C.	GORIZIA	MADDALUNO G.	PORTICI (NA)
LA ROSA S.	GELA (CL)	MAGGIOLINI P.	TERNI
LANZONI M.	MILANO	MAGGIOLO A.	GENOVA
LARCIPRETE P.	TERMOLI (CB)	MAGNI S.	ALBANO (RM)
LATINO C.	ROMA	MAGNO C.	AVELLINO
LAVORGNA F.	TELESE (BN)	MAIATICO A.	BENEVENTO
LAZZARETTI G.P.	MILANO	MANGOGNA S.	BOLZANO
LECCE P.	ISOLA DELL'IRI (FR)	MANTI B.	SOVERATO (CA)
LECCISO F.	LECCE	MARANELLI C.	NAPOLI
LEO A.	TARANTO	MARCELLI P.	TERNI
LEONE F.	BORGOSATOLLO (BS)	MARCENARO R.	GENOVA-BOLZANETO
LEVI RADAELLI E.	MILANO	MARCHETTI D.	FERMO (AP)
LIBARDI L.	CASELLE DI SOMMA CAMPAGNA (VR)	MARCHETTI F.	PISA
LICATA M.	CALTANISETTA	MARCHETTINI F.	S. REMO (IM)
LICCIARDI F.	MARANO DI NAPOLI (NA)	MARCHISIO C.	FOSSANO (CN)
LIETO S.	PRATOLA SERRA (AV)	MARIANI G.	MONZA (MI)
LIPARI V.	CASTELVETRANO (TP)	MARINARO A.M.	ALGHERO (SS)
LIPIRA S.	ACIREALE (CT)	MARINI M.	TRIESTE
LO NIGRO G.	GRAVINA (CT)	MARIOTTI E.	GENOVA-CORNIGLIANO
LODDE M.	COLFOSCO (TV)	MARONGIU C.	LA SPEZIA
LOFFI G.	CREMONA	MARRA F.	REGGIO CALABRIA
LONGO G.	MESSINA	MARTINETTI M.	CARBONIA (CA)
LOPEZ F.	MARGHERITA DI SAVOIA (FG)	MASOTTI G.P.	VILLANOVA (RA)

MASSARI C.A.	FERRARA	MONDINI S.	MAROTTA (PS)
MASTROMONACO B.	CAMPOBASSO	MONTANARI M.	REGGIO EMILIA
MATRA' A.	BOLOGNA	MONTANELLI G.	GELLO (PI)
MATTAZZI F.	CASTELVETRANO (TP)	MONTANILE C.	AVELLINO
MAURO M.	LITTA PARODI (AL)	MONTELLA R.	NAPOLI
MAURO R.	LECCE	MONTI M.	CAVALLASCA (CO)
MAZZOTTI G.P.	VILLANOVA DI B. CAVALLO (RA)	MORETTI W.	CAPRIOLO (BS)
MAZZUCCA L.	COSENZA	MORIZIO R.	BARI
MELANDRI T.	CESENATICO (FO)	MOROLLI G.	RIMINI (FO)
MELITO M.	NAPOLI	MOSCOLONI M.	FALCONARA (AN)
MELLACE A.	SOVERATO (CZ)	MOSCUZZA F.	SIRACUSA
MELODIA N.	BARI	MOSTARDINI M.	ARICCIA (RM)
MEMBRINI F.	VARESE	MUNARIN F.	MONTEBELLUNA (TV)
MENARDO A.	LENDINARA (RO)	MURRONI L.	CORTOGHIANA (CA)
MENGOLI P.	BOLOGNA	MUSELLA C.	S. GIORGIO A CREMANO (NA)
MERCENARO M.G.	SELARGIUS (CA)	MUSENGA C.	CAMPOBASSO
MERICI G.	UDINE	MUSURACA G.	TORRE DEL BENACO (VR)
MEROLLA E.	NAPOLI	NANIA F.	CATANZARO
MESCOLINI L.	REDONDESCO (MN)	NAPOLI M.	PIEVEBELVICINO (VI)
MIELE P.	AVELLINO	NARDIELLO P.	PORTICI (NA)
MIGLIAZZA F.	MILANO	NASSUATO G.	PADOVA
MILIANO G.	MILANO	NAVARRIA S.	UDINE
MINAUDO G.	PALERMO	NEBBIA A.	CAMPOBASSO
MINUSCOLI G.	ABAZIA VALLALTA (BG)	NERI F.	NOVARA
MISEROTTI G.	PIACENZA	NICOLINI P.	ROVIGO
MISURACA G.	APRIGLIANO (CS)	NIZZOLI A.	ROVELLO PORRO (CO)
MOCERINO M.	GRANDATE (CO)	NOBILE L.	VITINIA (RM)
MODOLO C.	SUSEGANA (TV)	NORA A.	PONTECORVO (FR)
MOGLIA S.	MILANO	OLIVARI L.	GENOVA
MOLINARI M.	PAVIA	OLIVETI A.	SENIGALLIA (AN)
MONDELCI M.	MOIE (MAIOLATI SPONTINI) (AN)	OPPO C.	ORISTANO

ORAZZO F.	NAPOLI	PELISSERO S.	TORINO
ORI F.	SUSENIA (TV)	PELLEGRINO P.	BOVES (CN)
ORIENTI S.	BOLOGNA	PELLOSO M.	VERONA
ORLANDO A.	MARANO DI NAPOLI (NA)	PELOSI A.	ARIANO IRPINO (AV)
ORLANDO G.	TARANTO	PELOSI F.P.	NAPOLI
ORSENIGA R.	MONZA (MI)	PELTRONE E.	BADOLATO (CZ)
OTTAVIANI M.	SOMMA CAMPAGNA (VR)	PEPI A.	MONTECOSARO (MC)
PAGLIARANI V.	CESENATICO (FO)	PEREGO C.	MONZA (MI)
PAINI G.	VOLTA MANTOVANA (MN)	PERNA V.	PORTICI (NA)
PALERMO P.	S. VITO (TA)	PEROTTI E.	TORRE D'ISOLA (PV)
PALLADINO A.	NAPOLI	PEROZZI I.	ZOLA PREDOSA (BO)
PALMIERI A.	NAPOLI	PERRELLA S.	TORRE DEL GRECO (NA)
PALMIERI G.	CAMPOBASSO	PESARO A.	TORBOLE CASAGLIO (BS)
PALMIERI L.	BARI	PETRACCA M.	ROVIGO
PALMIERO S.	BUGNARA (AQ)	PETRARCA F.	CAMPOBASSO
PALOMBI M.	BENEVENTO	PETRETTI M.R.	BENEVENTO
PANARESE F.	BENEVENTO	PETRONIO E.	TRIESTE
PANTALEO G.	FASANO (BR)	PETRUZZIELLA V.	PRATA P.V. (AV)
PANTO' E.	MESSINA	PETTENE A.	PAVIA
PAOLELLA G.B.	CATANIA	PETTOROSSI R.	MILANO
PAOLETTI PELLEGRI F.	S. TERENZO (SP)	PIERI R.	CESENA (FO)
PAPAGNO F.	BRESCIA	PIETRONI P.	ANCONA
PARISI M.	ROMA	PILETTI M.	MILANO
PARLANGELI T.	NOVOLI (LE)	PIOLI R.	REGGIO EMILIA
PASQUALITTO P.	VEROLI (FR)	PIRRONE G.	CATANIA
PASQUALUCCI S.	L'AQUILA	PISCAGLIA A.	RIMINI (FO)
PATIMO R.	MOLFETTA (BA)	PISCITELLI A.	NAPOLI
PAVANETTO N.	PREGANZIOL (TV)	PISTOIA S.	VERCELLI
PAVONE V.	BARI	PLATTO B.	SAREZZO (BS)
PEDALINO G.	CATANIA	POGGIO F.	GENOVA RIVAROLO
PEDOTO M.C.	BENEVENTO	POLICICCHIO G.	COSENZA

POLITO A.	TEANO (LE)	ROMANO V.	NAPOLI
POMPIGNOLI P.	FALCONARA (AN)	ROMEO N.	MESSINA
PORELLO G.	BIELLA (VC)	ROSAI G.	MILANO
PORTOGHESE F.	BARI	ROSATO F.	BOLOGNA
PROCICCHIANI R.	RONCHI DEI LEGIONARI (GO)	ROSSANO G.	NAPOLI
PUCCETTI M.	SPINA (PG)	ROSSI ERBA M.	BRESCIA
PUDDU G.	GONNESA (CA)	RUBINI S.	BOLOGNA
PUGNETTI G.	FINO MORNASCO (CO)	RUGGERI P.	PIACENZA
PULITANO' G.	MESSINA	RUSCICA R.	TERNI
PULZONE A.	AVELLINO	RUSSO C.	S. GIORGIO A CREMANO (NA)
QUAGLIO M.	ROVIGO	RUSSO M.	NAPOLI
QUARTO M.	TARANTO	SABATINI M.	ALBANO LAZ. (RM)
QUATTROCIOCCHI P.	VEROLI (FR)	SABUSCO G.	CAMPOBASSO
RACCONE A.	TORTONA (AL)	SAGGESE C.	NAPOLI
RAFFA M.	S. REMO (IM)	SALVANESCHI M.	PAVIA
RAINERI L.	NAPOLI	SALVATORI A.	POMEZIA (RM)
RAPETTI R.	OVADA (AL)	SANGIUOLO A.	SANT'ANGELO A CUPOLO (BN)
REGGIANI N.	REGGIO EMILIA	SANTARINI F.	RIMINI (FO)
RESTA F.	BARI	SANTINI G.	RICCIONE (FO)
RICCI E.	ALESSANDRIA	SATTA P.G.	CAGLIARI
RIEGLER G.	NAPOLI	SAVIO F.	PALERMO
RIMA S.	LECCE	SAYA A.	SIRACUSA
RINALDI M.	NAPOLI	SCANDURRA S.	BOLOGNA
RIPA G.	TARANTO	SCARPONI T.	PERUGIA
RIVA M.G.	MONZA (MI)	SCHENONE R.	GENOVA
RIZZO F.	S. MARTINO V.A. (VR)	SCHIAVON C.	VILLAFRANCA PADOVANA (PD)
RIZZOLO M.	MONZA (MI)	SCHIROSA G.	MONTALBANO IONICO (MT)
ROBUTTI U.	SPINETTA MARENGO (AL)	SCIACCA R.	TORINO
ROFFO A.	LA SPEZIA	SCIACCHITANO D.	MUZZANA DEL TURGNANO (UD)
ROMANO F.	POZZUOLO DEL FRIULI (UD)	SCOGNAMIGLIO E.	NAPOLI
ROMANO F.	LAVAGNA (GE)	SCOPA G.	NAPOLI

SCORBA C.	CASCIAGO (VA)	STERNI P.	PORTICI (NA)
SECHI A.	ALGHERO (SS)	SULLO S.	CASTELVETERE (AV)
SEDITA E.	CALTANISETTA	SUPPA A.	MARTELLAGO (VE)
SEMINI L.	MILANO	TABACCO A.	MONFALCONE (GO)
SERGI S.	REGGIO CALABRIA	TAFFURELLI U.	CASTEL MAGGIORE (BO)
SERINO R.	NAPOLI	TARANTINI G.	RIVA LIGURE (IM)
SERPE A.	NAPOLI	TARANTINO G.	NAPOLI
SERRAGLINI A.	PISA	TASCA A.	CARTIGLIANO (VI)
SEVERIN E.	MONTEBELLUNO (TV)	TEDINO F.	FORLI'
SGRELLI V.	PERUGIA	TEODORO N.	GARDIGIANO (VE)
SICA S.	MILANO	TIBY V.	MILANO
SIGNORINI G.	RICCIONE (FO)	TOLA P.	SASSARI
SILVESTRI V.	PICCIONE (PG)	TOMASSETTI Q.	ANCONA
SIMONCINI L.	UDINE	TOMMASINI U.	GORIZIA
SIMONI S.	CASTELLANA GROTTE (BA)	TONATTO A.	TORINO
SIMULA G.L.	ALGHERO (SS)	TONELLI F.	SESTO S. GIOVANNI (MI)
SINI G.P.	ROMA	TORCHIA F.	REGGIO CALABRIA
SIRAGUSA G.	CATANIA	TORELLI S.	SENIGALLIA (AN)
SIRITO D.	GENOVA-SESTRI PONENTE	TORTA G.	ROMA
SOMMARUGA N.	MILANO	TORTI P.	GENOVA-RIVAROLO
SORBO M.	PORTICI (NA)	TOSCANO R.	UDINE
SORIANI M.	ROMA	TOSI B.	VERONA
SORRENTINO A.	NAPOLI	TOSI F.	BELFORTE DEL CHIENTI (MC)
SPADANUDA A.	MONTEMITRO (CB)	TOTO F.	COLFELICE (FR)
SPAGNUOLO A.	NAPOLI	TOZZI R.	BOLOGNA
SPATA G.	REBBIO (CO)	TRAPUZZANO O.	SOVERATO (CZ)
SPINA A.	AVELLINO	TROFA G.	S. GIORGIO DEL SANNIO (BN)
SPINNATO L.	BOLOGNA	TROVO R.	REANA DEL ROYALE (UD)
SPIRITU N.	ALGHERO (SS)	TULIMIERO G.	AVELLINO
STARACE G.	NAPOLI	TURCHI R.	ROMA
STEGAGNO M.	S. LAZZARO DI SAVENA (BO)	UKMAR F.	TRIESTE

ULZEGA E.	MONSERRATO (CA)	VINCIGUERRA R.	MATERA
URAGO M.	TARANTO	VISCA G.	FONTANALIRI (FR)
USAI A.	ORISTANO	VITANTONIO T.	LIMOSANO (CB)
VACCA G.	VILLACIDRO (CA)	VITELLOZZI P.	ROMA
VALLERA G.	PRATOLA PELIGNA (AQ)	VITIELLO A.	DOLIA NOVA (CA)
VALSENTI C.	S. GEMINI (TR)	VITIELLO M.	QUARTUCCIU (CA)
VARIA M.	CASTELVETRANO (TP)	VOLPE A.	AVELLINO
VENDRAMINETTO E.D.	CARTIGLIANO (VI)	ZACCONE L.	CUNEO
VENEZIANI G.	ROMA	ZAGARELLA G.	PETRIGNANO D'ASSISI (PG)
VENTURA P.	BERGAMO	ZALEAGNA C.	ROMA
VENTURELLI G.	ZOLA PREDOSA (BO)	ZALTRON V.	SCORZE' (VE)
VENTURI L.	ROMA	ZAMO' C.	CASTELNUOVO DEL GARDA (VR)
VENUTI E.	MIRABELLA ECLANO (AV)	ZANETTI G.P.	LUGAGNANO (VR)
VERNAGLIONE C.	TARANTO	ZANGRANDO A.	LATISANA (UD)
VERRI A.	BOLOGNA	ZANOTTA G.	BARRA (NA)
VEZZU' L.	SAN GIORGIO IN BOSCO (PD)	ZEMA F.	REGGIO CALABRIA
VIASCO P.	LA SPEZIA	ZERELLA F.	BENEVENTO
VIGANO' A.	PISA	ZUCCA G.	CAGLIARI
VIGORELLI R.	MILANO	ZUCCHI S.	PIACENZA
VILLANO A.	VENTICANO (AV)		

UNITA' RICERCA E SVILUPPO - GIPHARMEX S.p.A. - MILANO

==

BRUN M.

BRUNETTI G.

FRIGERIO G.

GHEZZI C.

POZZOLI M.

MULTICENTRIC RESEARCH ON LACTOPRODUCER ENTEROCOCCUS SF68: PREVENTIVE ACTIVITY IN ANTIBIOTIC-ASSOCIATED DIARRHEA AND THERAPEUTIC EFFICACY IN ACUTE ENTERITIS ─ CONTROLLED DOUBLE-BLIND AND OPEN STUDY ─

INTRODUCTION

Intestinal dismicrobism as a result of oral or parenteral administration of antibiotics may easily bring about diarrhea (1); although these forms are generally easily reversible, there is no shortage of reports of more severe cases right up to the appearance of pseudomembranous colitis (2).
Intestinal flora is in fact one of the most valid defense mechanisms against the lodging of enteropathogens (3), as demonstrated by the lower microbial charge of pathogenic germs that is necessary to induce intestinal infection when the inoculum is subsequent to the administration of antibiotics (4).
Intestinal bacterial flora in fact, and in particular lactic acid bacteria, are able to inhibit the growth of enteropathogens germs through the production of organic acids and bacteriocins, lowering the pH and redox potential and competing for energy sources (5).
Appropriate measures to repopulate the intestinal lumen are then of primary interest in the prevention of diarrhea caused by antibiotics (1, 5) and, in addition to measures that restore the salt and water balance, they are also effective in the treatment of enteritis.
In these forms, in fact, the indiscriminate use of antibiotics has limited indications, often resulting in unnecessary and not rarely harmful, since this causes dismicrobism in an area where there is already an alteration of the endogenous bacterial flora that causes selection of antibiotic-resistant mutants.
Controlled clinical trials have indeed demonstrated the effectiveness of administering vital enterococci SF68 in the treatment of diarrheal enteritis in the adult (7-10), in the child (11-14), as well as in the prevention of intestinal dismicrobisms as a consequence of antibiotics use (15).
Enterococci lacto producers strain SF68 (BIOFLORIN of Giuliani S.A. Lugano - Switzerland, represented in Italy by Gipharmex S.p.A. Milan) have particular favorable biological characteristics:

- stability at acidic pH (16, 17)
- high speed of replication (19 minutes) (16)
- ability to colonize the human intestinal environment rapidly (18)
- absence of pathogenicity and invasiveness (16)
- resistance, of non–transferable type, to numerous antibiotics (16, 17)
- inhibitory ability against various enteropathogens germs (16, 17, 19) carried out by means of phenomena of biological competition, of intestinal acidification as well as through the production of biologically active substances or bacteriocins (19, 20)
- immunomodulatory (21, 22) and antiviral (23, 24) properties.

PATIENTS AND METHODS

Study protocols

In order to acquire further documented information about the preparation, it is desired to evaluate on a large case series, and then by means of a large multicentric investigation, on one side the effectiveness of Bioflorin on the prevention of diarrhea caused by antibiotics (preventive studies), on the other the therapeutic efficacy of the product on forms of acute diarrhea in adults and school-age children (therapeutic studies).

Each of these two studies was carried out by two types of complementary investigations: a controlled double-blind study, reserved for patients followed in hospital, and a open study involving patients followed in the clinics of physicians of territory.

Although a double-blind controlled study is the best method to guarantee the most reliable results, it is difficult to apply in investigations carried out at a territorial level: these play, on the other hand, particular importance for the evaluation of the preparation in the usual prescriptive conditions.

The study protocols proposed are reported in Table 1.

In the controlled studies, subjects were compared in progressive numeric order by assigning a random code number to each one.

The treatment consisted of taking 1 capsule x 2 times/day (preventive studies) or x 3 times/day (therapeutic studies) for a period of 7 days. In the controlled studies, lots No. 1 for Bioflorin and No. 1 for placebo were utilized; in the open studies, various batches available on the market were used.

The proposed clinic cards for recording the data gathered in the controlled researches are reported in Figure 1; similar clinical cards were utilized for the open study researches.

The studies of preventive type were intended to evaluate the efficacy of the preparation in reducing the incidence and/or severity of acute diarrhea episodes that were manifested within 7 days after the start of an antibiotic therapy, adopted for the treatment of respiratory, urinary or similar infection forms. The type of antibiotic and the route of administration adopted were assessed as well as the form and entity of diarrhea, intending as mild diarrhea the appearance of 2-3 daily discharges of liquid or semiliquid stool without mucous or blood, as moderate diarrhea the presence of 4-6 daily discharges prevalently liquid or sometimes with mucous, and as serious diarrhea the appearance of more than 6 daily liquid episodes, often accompanied by mucous and sometimes with blood.

The number of days of antibiotic therapy before the appearance of diarrhea was also taken into account, as well as the duration of the episode. Any eventual appearance of glossitis and/or labial and anal fissures were also noted.

In studies of therapeutic type the parameters proposed to evaluate the effectiveness of Bioflorin in the treatment of acute diarrhea forms were the number of defecations that occur daily, the stool consistency, the presence or absence of mucus in the stool, abdominal pain, meteorism, vomiting, nausea and fever; these parameters were ascertained before starting the treatment and daily within 7 days of therapy (Figure 1).

The evaluation of the results had to relate to the number of days of persistence of the various altered parameters, and in particular of a frequency of defecation greater than or equal to 3 times a day, and of a liquid or semi-liquid stool consistency: these two parameters were considered both separately and in a combined manner to better assess the condition of the bowel diarrhea.
Stool testing surveys were not included in the program study because of the difficulty to carry out such tests on all patients along with their scarce diagnostic significance of the clinical forms in question: the etiological classification then was essentially based on the opinion of the attending physician.

Processing and statistical analysis of data

In controlled studies the homogeneity of the two compared groups was evaluated by means of the X^2 test (25).
In the preventive type studies the statistical elaboration of the results was carried out by means of the X^2 test, while in the therapeutic type studies the estimated percentage of pathological persistence of the main parameters was elaborated by means of the" life-table": the difference of the trend of the curves in comparison was evaluated statistically by means of the Wilcoxon test (25).
The statistical analysis of the data was performed by the Biometric and Medical Statistic Unit of Gipharmex S.p.A. of Milan, by using IBM - PC and the programs Framework (Ashton -Tate) and SL- Micro (Questionnaire Service Company).

RESULTS

The clinical observations were made by a total of 469 physicians belonging to 150 Hospital Centers, and by 653 physicians of the territory. The total patients admitted to the study were 12289, grouped as follows :

Design	PREVENTIVE studies	THERAPEUTIC studies	TOTAL
CONTROLLED DOUBLE-BLIND	1397	1134	2531
OPEN	4665	5093	9758
TOTAL	6062	6227	12289

The characteristics of the patients studied and the assessment of the obtained results are listed here below, respectively for the controlled double-blind study of preventive type, the open study of preventive type, the controlled double-blind study of therapeutic type and the open study of therapeutic type.
The data relating to the tolerability of treatment with Bioflorin are outlined in the text; in the final table (Table 10) details of undesirable side-effects that occurred during treatment are reported: these took place in a small minority of cases, were non-specific and were equally distributed between the placebo group and the Bioflorin group.

1) CONTROLLED DOUBLE - BLIND STUDY OF PREVENTIVE TYPE

1397 patients were admitted to the controlled study, of whom 700 were treated with placebo and 697 with Bioflorin.
The distribution of patients according to sex, age, place of observation, infectious form, antibiotic treatment and route of administration adopted, is shown in Table 2: the homogeneity of the two groups in comparison, evaluated by means of X^2 test, showed no significant differences for any of the factors taken into consideration (Table 2, Figures 2 and 3).
The great majority of patients completed the study and only in a small minority of cases the treatment was interrupted because of the appearance of undesirable events, largely attributable to concomitant antibiotic treatment and in any case evenly distributed between the placebo group and the group treated with Bioflorin (Tables 2 and 10).

A summary of results is reported in Table 3.
A statistically significant difference in the frequency of the occurrence of diarrhea during the period of antibiotic treatment between the placebo group (16.2%) and the group treated with Bioflorin (8.6%) was observed, and also the severity of the diarrheal episodes appeared lower in the latter group (Figure 4).
No significant differences in the number of days after which diarrheal episodes appeared between the two groups were noted, (Table 3, Figure 5), while data relating to the duration of diarrhea showed statistically significant differences in favor of the Bioflorin (Table 3, Figure 6).

The frequency of the occurrence of diarrhea in relation to different types of antibiotics used is indicated in Figure 7.
The comparison between the preparations in study becomes statistically relevant in the larger groups, namely in those treated with cephalosporins, wide-spectrum amoxicillin and amino glycosides: in all these groups Bioflorin significantly reduced the incidence of diarrhea. The same trend was also reported in patients treated with cotrimoxazole , erythromycin and with various other antibiotics, even if at levels not significant for the small size of the sample group.
In patients treated with penicillin (75 cases) and with clinda-lincomycin (15 cases), no differences were observed between the placebo and Bioflorin groups.
Figure 8 shows the incidence of diarrhea in relation to the route of administration adopted: there were no statistically significant differences between the route of administration oral and parenteral, although overall there was a slightly more frequent occurrence of diarrhea during oral administration.
During the antibiotic therapy the appearance of glossitis was noted in 2.4 % of patients treated with Bioflorin with respect to 4.7% of patients in the placebo group ($p < 0.05$) and the appearance of fissures respectively in 1.2 % and 2.4 % (Table 3 and Figure 9).

2) OPEN STUDY OF PREVENTIVE TYPE

4665 patients were admitted to the open study, whose distribution by sex, age, infectious form, antibiotic treatment and route of administration is reported in Table 2 and in Figures 10 and 11.
The overwhelming majority of patients completed the study (93 %): undesired events appeared only in a small minority of cases, and they always appeared to be entirely nonspecific (Table 10).

A summary of results is shown in Table 3 .
The appearance of diarrhea was reported in a total of 11.5% of patients, resulting mild in the great majority of cases (82.2 %) (Table 3 and Figure 12).
The frequency of occurrence of diarrhea during treatment with different types of antibiotics and the severity of the diarrheal forms observed are reported in Figure 13.
In 2/3 of the patients diarrhea appeared by the third day of antibiotic therapy and the duration was not more than two days, lasting even only one day in 22 % of the cases (Table 3, Figures 14 and 15).
Glossitis were reported in 3.9 % of patients and fissures in 1.5 %.

3) CONTROLLED DOUBLE - BLIND STUDY OF THERAPEUTIC TYPE

1134 patients were admitted to the study, of whom 570 were treated with placebo and 564 with Bioflorin.
The distribution of patients by sex, age, place of observation, etiopathogenesis and case history is shown in Tables 4/a and 4/b and in Figures 16, 17, 18, 19.
The homogeneity of the two groups in comparison, evaluated by means of X^2 test, showed no significant differences for any of the factors taken into consideration.
The great majority of patients completed the study and only in a small minority of cases (6 in the placebo group and 2 in the Bioflorin group) the treatment was interrupted because of the appearance of undesirable events of various type (Table 10).

The evaluation by means of "life-table" of the estimated percentages of persistence of pathological parameters are shown in Tables 5, 6, 7, 8, 9.
Table 5 provides data on the persistence of bowel issues with defecation of 3 or more times a day, while Table 6 shows the data on the persistence of a liquid or semi-liquid consistency of stools.
Since a complete normalization of the bowel must include the return to normality both of the stool frequency and consistency, the data concerning this combined assessment are reported in Table 7.
The persistence of mucus in the stool and of abdominal pain are reported respectively in Table 8 and Table 9.
The difference of the trend of the curves in comparison, as evaluated by means of the Wilcoxon test, showed statistically significant differences between treatments relatively to all the parameters considered, and demonstrated a more rapid remission of the symptoms during treatment with Bioflorin compared to placebo (Figures 20, 21, 22, 23, 24).
The data regarding the persistence of nausea, vomiting, bloating and fever, while confirming a consistent trend in favor of Bioflorin, however did not demonstrate statistically significant differences, in part also in relation to the small size of the samples, these symptoms being present only in a part of cases.

4) OPEN STUDY OF THERAPEUTIC TYPE

5093 patients were admitted to the study, whose distribution by sex, age, etiopathogenesis and case history is shown in Tables 4/a and 4/b and in Figures 25, 26, 27, and 28.
The great majority of patients completed the study and only in 0.3% of cases the treatment was suspended due to the appearance of undesirable events, which however were hardly attributable to the treatment itself (Tables 4 and 10).

The curves of persistence of altered parameters observed during treatment with Bioflorin in the open study are expressed in Figure 29: the data concerning the treatment with Bioflorin and placebo obtained from the controlled study are also shown for comparison.
The comparison between the estimated percentage of persistent diarrhea in patients treated with Bioflorin and taking part in the open study and in patients taking placebo in the controlled study demonstrated a high statistical significance on the Wilcoxon test: W = -140818, Z= -8.707, $p < 0.01$: this finding, although it must necessarily be considered with caution, as it refers to two different sample groups, confirms the favorable clinical outcome of diarrheal diseases after treatment with Bioflorin.
All in all the ”Bioflorin curves" relating to the double-blind and open study were not significantly different; however, a tendency towards a faster remission of symptoms in patients belonging to the open study rather than in those who took part in the double-blind study was evident, probably in relation to the grater severity of the clinical form of these latter, in fact responsible for the admission to hospital.

COMMENT AND CONCLUSIONS

Thanks to the large sample population studied, the multicentric research conducted allowed to reconfirm the utility of a "probiotic" approach to the problem of diarrhea, by means of the administration of enterococci lactoproducers SF68 (7-14), in opposition to the "antibiotic" setting of traditional chemotherapy.

In the preventive-type studies it was possible to observe the non-negligible incidence of diarrheal disorders in the bowel during the course of antibiotic therapy: on average 16% of the patients in the control group, in fact, manifested diarrhea during treatment with all types of antibiotics considered, during administration of either oral or parenteral.

These observations are confirmed by the published data, from which it emerges the occurrence of diarrhea during treatment with different antibiotics, regardless of the route of administration adopted, with percentages of incidence that vary widely from Author to Author, ranging from 4 to 36% (1).

The administration of Bioflorin as a preventive measure during antibiotic treatment was proven effective in significantly reducing the frequency of such diarrheal diseases, while also lessening the severity of the diarrhea attacks.

The administration of Bioflorin for therapeutic purposes in the treatment of acute diarrheal diseases both in adults and children led to a more rapid recovery from the symptoms compared to placebo: this difference appeared statistically significant although the patient often spontaneously healed from this clinical form of the illness; this result is of particular interest since in such forms a therapy to shorten the clinical course of the disease can be prescribed, without necessarily resorting to an indiscriminate antibiotic therapy.

The results obtained in the open type studies, relating to a sample population that partly differs by age, severity of the clinical forms and antibiotic treatment of the disease, confirm the positive results reported in controlled trials, confirming the activity of the preparation in both preventive and therapeutic situations, while also emphasizing the safety and tolerability of such treatment.

Therefore the administration of enterococci lactoproducers SF68 proved effective not only in the prevention of intestinal dismicrobism following an antibiotic treatment, but also in the treatment of acute diarrheal forms, and this by restoring the protective action carried out by the intestinal bacterial flora against enteropathogens. The effectiveness of the treatment with Bioflorin, together with its tolerability and the absence of possible complications, which are instead not uncommon during antibiotic therapy (1, 2), confirm then the importance of an alternative approach to antibiotic therapy that enhances the ability of the defense of the intestinal flora.

SUMMARY

A large multicentric clinical study involving 12289 patients from 150 hospitals and 653 physicians of territory has been performed to assess the activity of a preparation containing vital enterococci lactoproducers of the strain SF68 (BIOFLORIN), in the prevention of antibiotic-associated diarrhea and in the treatment of acute enteritis.

Both studies of preventive type and studies of therapeutic type were carried out in two kinds of investigation having complementary meaning, one double-blind controlled study, performed on hospital patients, and an open study, involving patients followed by the physicians of territory in their local surgeries.

Compared to placebo, the administration of Bioflorin was proven to be effective in the prevention of intestinal dismicrobism resulting from the use of antibiotics, significantly reducing the incidence of diarrheal diseases resulting from the antibiotic therapy, while also determining a trend towards milder clinical forms of the disease.

The administration of Bioflorin for therapeutic purposes in acute enteritis led to a more rapid resolution of the symptoms compared to placebo.

The open studies confirmed the positive results observed in the controlled studies, underlining the efficacy and tolerability of an approach to diarrhea that restores the protective action carried out by the intestinal flora against enteropathogens.

TABLE 1 - Study protocols

	PREVENTIVE-TYPE STUDIES	THERAPEUTIC-TYPE STUDIES
AIM	Evaluation of the preventive efficacy in the antibiotic related diarrhea	Evaluation of the therapeutic efficacy in acute forms of diarrhea
STUDY DESIGN	1. Controlled double-blind study (Hospital Centers)<br>2. Open study (Physicians of territory)	1. Controlled double-blind study (Hospital Centers)<br>2. Open study (Physicians of territory)
COMPARATIVE PREPARATION	Placebo in study 1	Placebo in study 1
SELECTION CRITERIA	To be admitted:<br>- adults or school-age patients under antibiotic treatment	To be admitted:<br>- patients suffering from acute diarrheal enterocolitis of various origins without detectable signs of sepsis and/or localizations to other organs
	To be excluded:<br>- patients under tetracyclines treatment	To be excluded:<br>- diarrheas from food intolerance (gluten, lactose, lactoproteins, food allergens)<br>- infectious diarrheas caused by Salmonella typhi, Entamoeba histolytica, Giardia lamblia, parasitosis<br>- diarrheas with signs of sepsis (enteroinvasive strains)
STUDIED PARAMETERS	See card Mod. 700.13/C1 and 700.15/A1	See card Mod. 700.14/C2 and 700.16/A2
PREPARATION UNDER STUDY	BIOFLORIN<br>(enterococci LAB strain SF68 in freeze-dried form;<br>Each capsule contains at least 75 millions of cells)	
DOSAGE	1 capsule twice a day at mealtimes for 7 days	1 capsule three times a day for 7 days

TABELLA 2 - Studi di tipo PREVENTIVO. Descrizione della casistica

Disegno		CONTROLLATO DOPPIO-CIECO				APERTO
	TRATTAMENTI	Placebo (%)	Bioflorin (%)	Totale (%)	Test di omog.	Bioflorin (%)
NO. DI PAZIENTI AMMESSI ALLO STUDIO		700	697	1397	--	4665
Sesso	Femmine	305 (43.6)	299 (42.9)	604 (43.2)	X² = 0.040	2342 (50.2)
	Maschi	395 (56.4)	398 (57.1)	793 (56.8)	n.s.	2323 (49.8)
Età	< 14 anni	59 (8.4)	61 (8.8)	120 (8.6)	X² = 6.753	588 (12.6)
	14-20 "	29 (4.1)	19 (2.7)	48 (3.4)	n.s.	415 (8.9)
	21-40 "	99 (14.1)	104 (14.9)	203 (14.5)		1437 (30.8)
	41-60 "	142 (20.3)	166 (23.8)	308 (22.0)		1255 (26.9)
	61-70 "	141 (20.1)	115 (16.5)	256 (18.3)		555 (11.9)
	> 70 "	230 (32.9)	232 (33.3)	462 (33.1)		415 (8.9)
Pazienti	Ricoverati	636 (90.9)	634 (91.0)	1270 (90.9)	X² = 0.001	--
	Ambulatoriali	64 (9.1)	63 (9.0)	127 (9.1)	n.s.	4665 (100)
Forma infettiva	Respiratoria	431 (61.6)	421 (60.4)	852 (61.0)	X² = 0.225	2558 (54.8)
	Urologica	122 (17.4)	127 (18.2)	249 (17.8)	n.s.	1189 (25.5)
	Altre	147 (21.0)	149 (21.4)	296 (21.2)		918 (19.7)
Trattamento antibiotico	Cefalosporine	255 (36.4)	239 (34.3)	494 (35.4)	X² = 7.783	1045 (22.4)
	Ampi-amoxicillina	183 (26.1)	182 (26.1)	365 (26.1)	n.s.	1227 (26.3)
	Aminoglicosidi	76 (10.9)	82 (11.8)	158 (11.3)		359 (7.7)
	Cotrimossazolo	42 (6.0)	53 (7.6)	95 (6.8)		611 (13.1)
	Penicillina	44 (6.3)	31 (4.4)	75 (5.4)		313 (6.7)
	Eritromicina	28 (4.0)	27 (3.9)	55 (3.9)		438 (9.4)
	Clindamicina/Lincomicina	10 (1.4)	5 (.7)	15 (1.1)		229 (4.9)
	Altri	62 (8.9)	78 (11.2)	140 (10.0)		443 (9.5)
Via di somministr.	Parenterale	480 (68.6)	470 (67.4)	950 (68.0)	X² = 0.159	1320 (28.3)
	Orale	220 (31.4)	227 (32.6)	447 (32.0)	n.s.	3345 (71.7)
Sospensione anticipata	No	662 (94.6)	661 (94.8)	1323 (94.7)	X² = 2.720	4340 (93.0)
	Si, cause non inerenti	33 (4.7)	35 (5.0)	68 (4.9)	n.s.	279 (6.0)
	Si, effetti collaterali	5 (.7)	1 (.1)	6 (.4)		46 (1.0)

TABELLA 3 - Studi di tipo PREVENTIVO. Risultati

Disegno		CONTROLLATO DOPPIO-CIECO				APERTO
TRATTAMENTI		Placebo (%)	Bioflorin (%)	Totale (%)	Anal. statist.	Bioflorin (%)
NO. DEI CASI STUDIATI		662	661	1323		4340
DIARREA						
-Frequenza totale		107 (16.2)	57 (8.6)	164 (12.4)	X² = 16.63<br>p < 0.001	499 (11.5)
-Grado	lieve	66 (61.7)	44 (77.2)	110 (67.1)	X² = 4.860	410 (82.2)
	moderata	38 (35.5)	13 (22.8)	51 (31.1)	n.s.	79 (15.8)
	grave	3 (2.8)	0 (0.0)	3 (1.8)		10 (2.0)
-Comparsa dopo giorni	1	13 (12.1)	7 (12.3)	20 (12.2)	X² = 0.851	65 (13.0)
	2	42 (39.3)	19 (33.3)	61 (37.2)	n.s.	179 (35.9)
	3	26 (24.3)	15 (26.3)	41 (25.0)		157 (31.5)
	4	16 (15.0)	11 (19.3)	27 (16.5)		60 (12.0)
	> 4	10 (9.3)	5 (8.8)	15 (9.1)		38 (7.6)
-Durata per giorni	1	15 (14.0)	18 (31.6)	33 (20.1)	X² = 7.190	109 (21.8)
	2	38 (35.5)	17 (29.8)	55 (33.5)	p < 0.05	224 (44.9)
	> 2	54 (50.5)	22 (38.6)	76 (46.3)		166 (33.3)
GLOSSITI		31 (4.7)	16 (2.4)	47 (3.6)	X² = 4.253<br>p < 0.05	182 (3.9)
RAGADI		16 (2.4)	8 (1.2)	24 (1.8)	X² = 2.047<br>n.s.	70 (1.5)

TABELLA 4/a - Studi di tipo TERAPEUTICO. Descrizione della casistica

Disegno		CONTROLLATO DOPPIO-CIECO				APERTO
	TRATTAMENTI	Placebo (%)	Bioflorin (%)	Totale (%)	Test di omog.	Bioflorin (%)
NO. DI PAZIENTI AMMESSI ALLO STUDIO		570	564	1134	--	5093
Sesso	Femmine	274 (48.1)	278 (49.3)	552 (48.7)	X^2 = 0.124	2511 (49.3)
	Maschi	296 (51.9)	286 (50.7)	582 (51.3)	n.s.	2582 (50.7)
Età	< 14 anni	44 (7.7)	43 (7.6)	87 (7.7)	X^2 = 8.210	622 (12.2)
	14-20 "	18 (3.2)	13 (2.3)	31 (2.7)	n.s.	438 (8.6)
	21-40 "	86 (15.1)	115 (20.4)	201 (17.7)		1711 (33.6)
	41-60 "	154 (27.0)	141 (25.0)	295 (26.0)		1441 (28.3)
	61-70 "	101 (17.7)	111 (19.7)	212 (18.7)		474 (9.3)
	> 70 "	167 (29.3)	141 (25.0)	308 (27.2)		407 (8.0)
Pazienti	Ricoverati	466 (81.8)	447 (79.3)	913 (80.5)	X^2 = 0.975	--
	Ambulatoriali	104 (18.2)	117 (20.7)	221 (19.5)	n.s.	5093 (100)
Eziopatogenesi	Disordini alimentari	105 (18.4)	111 (19.7)	216 (19.0)	X^2 = 3.872	1838 (36.1)
	Infezioni	103 (18.1)	82 (14.5)	185 (16.3)	n.s.	922 (18.1)
	Cause iatrogene	85 (14.9)	87 (15.4)	172 (15.2)		504 (9.9)
	Tossinfezioni	60 (10.5)	51 (9.0)	111 (9.8)		652 (12.8)
	Variazioni climatiche	14 (2.5)	16 (2.8)	30 (2.6)		484 (9.5)
	Cause sconosciute	203 (35.6)	217 (38.5)	420 (37.0)		693 (13.6)
Sospensione anticipata	No	458 (80.4)	445 (78.9)	903 (79.6)	X^2 = 5.497	4525 (88.8)
	Si, guarigione anticipat.	76 (13.3)	95 (16.8)	171 (15.1)	n.s.	427 (8.4)
	Si, cause non inerenti	30 (5.3)	22 (3.9)	52 (4.6)		126 (2.5)
	Si, effetti collaterali	6 (1.1)	2 (.4)	8 (.7)		15 (.3)

TABELLA 4/b - Studi di tipo TERAPEUTICO. Dati basali

Disegno CONTROLLATO DOPPIO-CIECO APERTO

TRATTAMENTI		Placebo (%)	Bioflorin (%)	Totale (%)	Test di omog.	Bioflorin (%)
NO. DEI CASI		570	564	1134		5093
Numero di evacuazioni	3	128 (22.5)	130 (23.0)	258 (22.8)	X² = 0.475 n.s.	1181 (23.2)
	4	143 (25.1)	147 (26.1)	290 (25.6)		1294 (25.4)
	5	90 (15.8)	87 (15.4)	177 (15.6)		1069 (21.0)
	6	72 (12.6)	73 (12.9)	145 (12.8)		637 (12.5)
	> 6	137 (24.0)	127 (22.5)	264 (23.3)		912 (17.9)
Aspetto delle feci	Liquide	394 (69.1)	399 (70.7)	793 (69.9)	X² = 0.282 n.s.	3448 (67.7)
	Semiliq.	176 (30.9)	165 (29.3)	341 (30.1)		1645 (32.3)
Crampi e/o dolori addominali		387 (67.9)	386 (68.4)	773 (68.2)	X² = 0.018 n.s.	4074 (80.0)
Meteorismo		334 (58.6)	360 (63.8)	694 (61.2)	X² = 3.053 n.s.	3351 (65.8)
Nausea		241 (42.3)	246 (43.6)	487 (42.9)	X² = 0.156 n.s.	2124 (41.7)
Muco nelle feci		220 (38.6)	205 (36.3)	425 (37.5)	X² = 0.520 n.s.	2052 (40.3)
Febbre		220 (38.6)	197 (34.9)	417 (36.8)	X² = 1.486 n.s.	1625 (31.9)
Vomito		156 (27.4)	167 (29.6)	323 (28.5)	X² = 0.594 n.s.	1401 (27.5)

Tabella 5 - Studio CONTROLLATO DOPPIO-CIECO di tipo TERAPEUTICO.
Percentuali stimate di persistenza di una frequenza dell'alvo con 3 o più scariche/die

	PLACEBO				BIOFLORIN			
Intervallo di tempo (giorni)	Numero di casi			Percentuali stimate di persistenza (iniz.periodo)	Numero di casi			Percentuali stimate di persistenza (iniz.periodo)
	Inizio periodo	Guariti	Persi di vista		Inizio periodo	Guariti	Persi di vista	
0 - 1	570	118	--	100.0	564	156	--	100.0
1 - 2	452	114	4	79.3	408	124	4	72.3
2 - 3	334	94	11	59.2	280	103	7	50.2
3 - 4	229	77	6	42.3	170	60	2	31.5
4 - 5	146	50	3	27.9	108	37	--	20.3
5 - 6	93	29	--	18.2	71	27	2	13.4
6 - 7	64	20	--	12.5	42	9	--	8.2
> 7	44	--	44	8.6	33	--	33	6.5

ANALISI STATISTICA (WILCOXON TEST) W = 40052 ; Z = 3.746 ; $p < 0.01$

Tabella 6 - Studio CONTROLLATO DOPPIO-CIECO di tipo TERAPEUTICO.
Percentuali stimate di persistenza di feci liquide o semiliquide

Intervallo di tempo (giorni)	PLACEBO Numero di casi			PLACEBO Percentuali stimate di persistenza (iniz.periodo)	BIOFLORIN Numero di casi			BIOFLORIN Percentuali stimate di persistenza (iniz.periodo)
	Inizio periodo	Guariti	Persi di vista		Inizio periodo	Guariti	Persi di vista	
0 - 1	570	68	--	100.0	564	87	--	100.0
1 - 2	502	110	6	88.1	477	153	5	84.6
2 - 3	386	114	11	68.7	319	112	7	57.3
3 - 4	261	91	5	48.1	200	78	3	37.0
4 - 5	165	69	1	31.2	119	48	--	22.4
5 - 6	95	28	--	18.1	71	30	1	13.4
6 - 7	67	20	--	12.8	40	7	--	7.7
> 7	47	--	47	8.9	33	--	33	6.3

ANALISI STATISTICA (WILCOXON TEST) W = 43899 ; Z = 4.130 ; p < 0.01

Tabella 7 - Studio CONTROLLATO DOPPIO-CIECO di tipo TERAPEUTICO.
Percentuali stimate di persistenza di alvo diarroico (valutazione combinata dei caratteri frequenza dell'alvo e consistenza delle feci)

Intervallo di tempo (giorni)	PLACEBO Numero di casi			PLACEBO Percentuali stimate di persistenza (iniz.periodo)	BIOFLORIN Numero di casi			BIOFLORIN Percentuali stimate di persistenza (iniz.periodo)
	Inizio periodo	Guariti	Persi di vista		Inizio periodo	Guariti	Persi di vista	
0 - 1	570	45	--	100.0	564	68	--	100.0
1 - 2	525	88	6	92.1	496	136	5	87.9
2 - 3	431	111	13	76.6	355	120	7	63.7
3 - 4	307	96	7	56.6	228	87	3	42.0
4 - 5	204	78	3	38.7	138	50	--	25.8
5 - 6	123	36	--	23.8	88	35	2	16.5
6 - 7	87	26	--	16.8	51	11	--	9.8
> 7	61	--	61	11.8	40	--	40	7.7

ANALISI STATISTICA (WILCOXON TEST) W = 56914 ; Z = 5.376 ; $p < 0.01$

Tabella 8 - Studio CONTROLLATO DOPPIO-CIECO di tipo TERAPEUTICO.
Percentuali stimate di persistenza di muco nelle feci

	PLACEBO				BIOFLORIN			
Intervallo di tempo (giorni)	Numero di casi			Percentuali stimate di persistenza (iniz.periodo)	Numero di casi			Percentuali stimate di persistenza (iniz.periodo)
	Inizio periodo	Guariti	Persi di vista		Inizio periodo	Guariti	Persi di vista	
0 - 1	220	32	--	100.0	205	37	--	100.0
1 - 2	188	40	4	85.5	168	47	3	82.0
2 - 3	144	44	4	67.1	118	41	--	58.8
3 - 4	96	29	3	46.3	77	28	1	38.4
4 - 5	64	11	2	32.1	48	13	--	24.3
5 - 6	51	14	--	26.5	35	11	--	17.7
6 - 7	37	5	--	19.2	24	4	--	12.2
> 7	32	--	32	16.6	20	--	20	10.1

ANALISI STATISTICA (WILCOXON TEST) W = 4953 ; Z = 2.036 ; $p < 0.05$

Tabella 9 - Studio CONTROLLATO DOPPIO-CIECO di tipo TERAPEUTICO.
Percentuali stimate di persistenza di dolore addominale

Intervallo di tempo (giorni)	PLACEBO: Numero di casi			PLACEBO: Percentuali stimate di persistenza (iniz.periodo)	BIOFLORIN: Numero di casi			BIOFLORIN: Percentuali stimate di persistenza (iniz.periodo)
	Inizio periodo	Guariti	Persi di vista		Inizio periodo	Guariti	Persi di vista	
0 - 1	387	65	--	100.0	386	82	--	100.0
1 - 2	322	113	5	83.2	304	113	3	78.8
2 - 3	204	74	3	53.8	188	81	3	49.3
3 - 4	127	36	2	34.1	104	43	--	27.9
4 - 5	89	35	1	24.4	61	20	1	16.4
5 - 6	53	11	--	14.7	40	12	1	11.0
6 - 7	42	6	--	11.7	27	7	--	7.6
> 7	36	--	36	10.0	20	--	20	5.7

ANALISI STATISTICA (WILCOXON TEST) W = 12673 ; Z = 2.115 ; $p < 0.05$

TABELLA 10 - Eventi indesiderati

Disegno	CONTROLLATO DOPPIO-CIECO						APERTO	
**Studi di tipo PREVENTIVO**	**PLACEBO**		**BIOFLORIN**		**TOTALE**		**BIOFLORIN**	
No. pz. con eventi ind./no.totale pz.	14/700 (2.0%)		10/697 (1.4%)		24/1397 (1.7%)		344/4665 (7.4%)	
Sospensione anticipata del trattam.	SI	NO	SI	NO	SI	NO	SI	NO
	5	9	1	9	6	18	46	298
Descrizione -Meteorismo	-	2	-	4	-	6	5	29
-Nausea	1	1	-	2	1	3	8	63
-Vomito	2	1	1	-	3	1	2	31
-Esantema, prurito	-	1	-	2	-	3	3	27
-Dolore addominale	1	1	-	1	1	2	10	48
-Disturbi gastrici	1	2	-	-	1	2	13	62
-Artralgia	-	1	-	-	-	1	-	-
-Cefalea	-	-	-	-	-	-	5	38
**Studi di tipo TERAPEUTICO**	**PLACEBO**		**BIOFLORIN**		**TOTALE**		**BIOFLORIN**	
No. pz. con eventi ind./no.totale pz.	8/570 (1.4%)		6/564 (1.1%)		14/1134 (1.2%)		238/5093 (4.7%)	
Sospensione anticipata del trattam.	SI	NO	SI	NO	SI	NO	SI	NO
	6	2	2	4	8	6	15	223
Descrizione -Disturbi gastrici	1	-	-	3	1	3	1	44
-Dolore addominale	2	-	1	-	3	-	5	33
-Esantema, prurito	1	1	-	1	1	2	-	15
-Vomito	1	-	1	-	2	-	4	30
-Cefalea	-	1	-	-	-	1	2	39
-Stipsi	1	-	-	-	1	-	-	2
-Nausea	-	-	-	-	-	-	3	60

FIGURA 1 - Schede cliniche adottate per gli studi CONTROLLATI

SCHEDA CLINICA mod. 700.13/C1

Data |_|_| |_|_| |_|_|

STUDIO CONTROLLATO DI TIPO PREVENTIVO CON SF 68

La scheda clinica mod. 700.13/C1 è stata predisposta per annotare i dati riguardanti il paziente e la sua situazione clinica di base nonchè l'eventuale comparsa di fenomeni secondari al trattamento antibiotico, che rappresenta l'oggetto dello studio.

Da non compilare

- OSPEDALE ______________________
- MEDICO (firma) ______________________

|_|_|_|_|_| cc — |_|_| pz

- PAZIENTE:

Iniz. |_|_|_| - Sesso M |m| F |f| - Età (anni) |_|_|

- Tel. ______________________

Ambulatoriale |a| Ricovero |r|

FORMA CLINICA richiedente trattamento antibiotico:

- respiratoria |R| - urologica |U| - altre |A|

Descrizione ______________________

- Trattamento antibiotico (barrare 1 sola casella):

- Penicillina	P	- Ampi-amoxicillina	A
- Clindamicina/Lincomicina	L	- Eritromicina	E
- Cefalosporine	C	- Cotrimossazolo	T
- Aminoglicosidi	G	- Altri: indicare ________	V

- Via di somministrazione: - orale |O| - parenterale |P|

- TRATTAMENTO DI TIPO PREVENTIVO CON **"PREVEN - LAB"**

dotazione n. |_|_|_|_|_| (1 cps x 2/die ai pasti, per 7 giorni)

|P| |B|

- Interruzione anticipata del trattamento:

NO	n
SI per guarigione	g
SI per effetti collaterali	s
SI per motivi non inerenti	e

- RILIEVI CLINICI

- Comparsa di DIARREA nei 7 giorni seguenti l'inizio del trattamento antibiotico:

NO |0| - SI lieve |1| - Si moderata |2| - SI grave |3|

Se SI, dopo quanti giorni: |_|

Se SI, per quanti giorni: |_|_|

- Comparsa di:
  - Glossiti NO |n| - SI |y|
  - Ragadi labiali o anali NO |n| - SI |y|

- Effetti collaterali: NO |n| - SI |y|

se SI , quali? ______________________ |_|_|

Note - La diarrea è intesa come presenza di almeno 2-3 scariche giornaliere di feci liquide e non formate:
- diarrea lieve: 2-3 scariche liquide o semiliquide senza muco e/o sangue
- diarrea moderata: 4-6 scariche prevalentemente liquide o saltuariamente con muco
- diarrea grave: più di 6 scariche liquide, spesso con muco, talora con sangue.

Da inoltrare a: GIPHARMEX Direzione Medica e Ricerca
20129 MILANO - Via Palagi 2 - tel. 02/20541

FIGURA 1 - (Segue)

SCHEDA CLINICA mod. 700.14/C2

STUDIO CONTROLLATO DI TIPO TERAPEUTICO CON SF 68

La scheda clinica mod. 700.14/C2 è stata predisposta per annotare i dati riguardanti il paziente e la sua situazione di base nonchè l'evoluzione del quadro clinico nel corso dello studio.
Per facilitare le rilevazioni, è opportuno consegnare al paziente ambulatoriale l'apposito diario giornaliero di semplice compilazione: il paziente avrà cura di restituirlo al Medico che completerà in tal modo la scheda clinica.

	Da non compilare
- OSPEDALE ____<br>- MEDICO (firma) ____	cc / pz
- PAZIENTE:<br>Iniz. [ ] - Sesso M [m] F [f] - Età (anni) [ ]<br>- Tel. ____<br>Ambulatoriale [a] Ricovero [r]<br>DIAGNOSI - Forma diarroica dovuta a:<br>- infezioni I - tossinfezioni T<br>- disordini alimentari A - cause iatrogene F<br>- variazioni climatiche C - cause sconosciute U	
- TRATTAMENTO TERAPEUTICO CON **"TRATEN - LAB"**<br>dotazione n. [ ] (1 cps x 3 volte al giorno in corrispondenza dei pasti, per 7 giorni)<br>- Interruzione anticipata del trattamento: NO n / SI per guarigione g / SI per effetti collaterali s / SI per motivi non inerenti e	[P] [B]

- RILIEVI CLINICI

Giorni		0 (basale)	1	2	3	4	5	6	7	DB	TPA
- Numero delle evacuazioni		[ ]	[ ]	[ ]	[ ]	[ ]	[ ]	[ ]	[ ]	[ ]	[ ]
- Feci	a) liquide	☐	☐	☐	☐	☐	☐	☐	☐	[ ]	[ ]
	b) semiliquide	☐	☐	☐	☐	☐	☐	☐	☐		
	c) semisolide	☐	☐	☐	☐	☐	☐	☐	☐		[ ]
	d) solide/formate	☐	☐	☐	☐	☐	☐	☐	☐		itg.
- Muco nelle feci	- NO	☐	☐	☐	☐	☐	☐	☐	☐	[ ]	[ ]
	- SI	☐	☐	☐	☐	☐	☐	☐	☐		
- Crampi e/o dolori addominali	- NO	☐	☐	☐	☐	☐	☐	☐	☐	[ ]	[ ]
	- SI	☐	☐	☐	☐	☐	☐	☐	☐		
- Meteorismo	- NO	☐	☐	☐	☐	☐	☐	☐	☐	[ ]	[ ]
	- SI	☐	☐	☐	☐	☐	☐	☐	☐		
- Vomito	- NO	☐	☐	☐	☐	☐	☐	☐	☐	[ ]	[ ]
	- SI	☐	☐	☐	☐	☐	☐	☐	☐		
- Nausea	- NO	☐	☐	☐	☐	☐	☐	☐	☐	[ ]	[ ]
	- SI	☐	☐	☐	☐	☐	☐	☐	☐		
- Febbre	- NO	☐	☐	☐	☐	☐	☐	☐	☐	[ ]	[ ]
	- SI	☐	☐	☐	☐	☐	☐	☐	☐		

- Effetti collaterali: NO [n] SI [y]
se SI, quali? ____ [ ]

Da inoltrare a: GIPHARMEX Direzione Medica e Ricerca
20129 MILANO - Via Palagi 2 - tel. 02/20541

FIGURA 2 - Studio CONTROLLATO DOPPIO-CIECO di tipo PREVENTIVO
Distribuzione per età (anni) nei due gruppi di pazienti

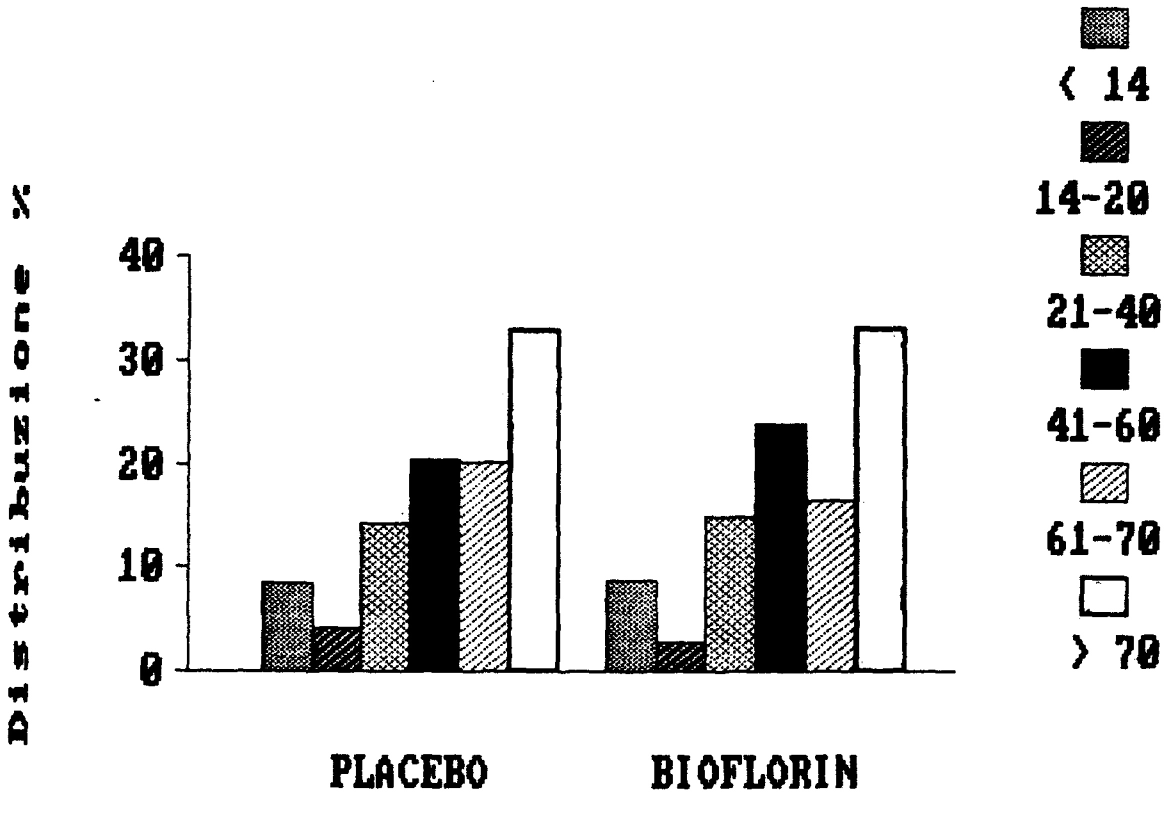

FIGURA 3 - Studio CONTROLLATO DOPPIO-CIECO di tipo PREVENTIVO
Antibiotici impiegati nei due gruppi di pazienti a confronto

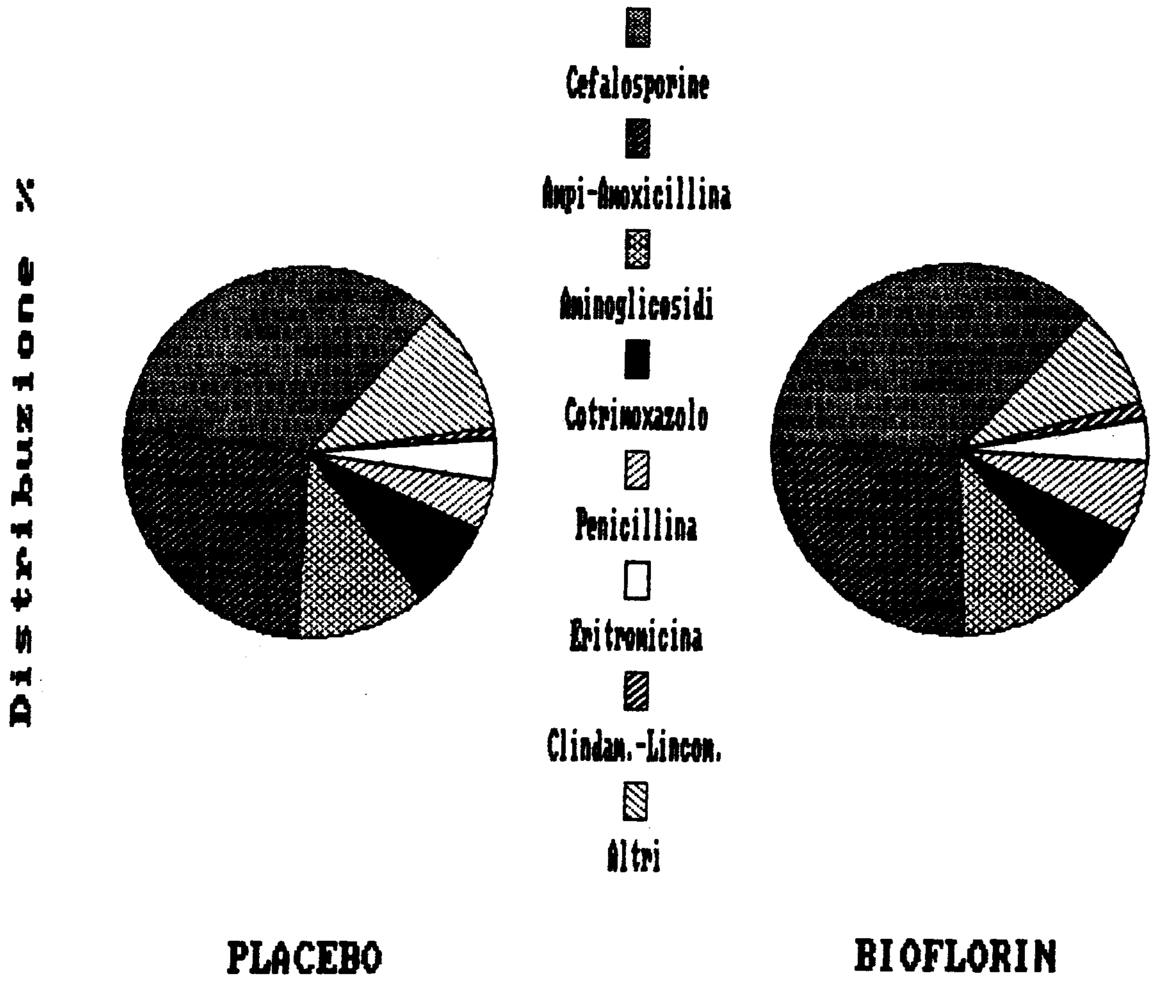

FIGURA 4 - Studio CONTROLLATO DOPPIO-CIECO di tipo PREVENTIVO Gravità della forma diarroica durante trattamento con antibiotici: confronto PLACEBO - BIOFLORIN

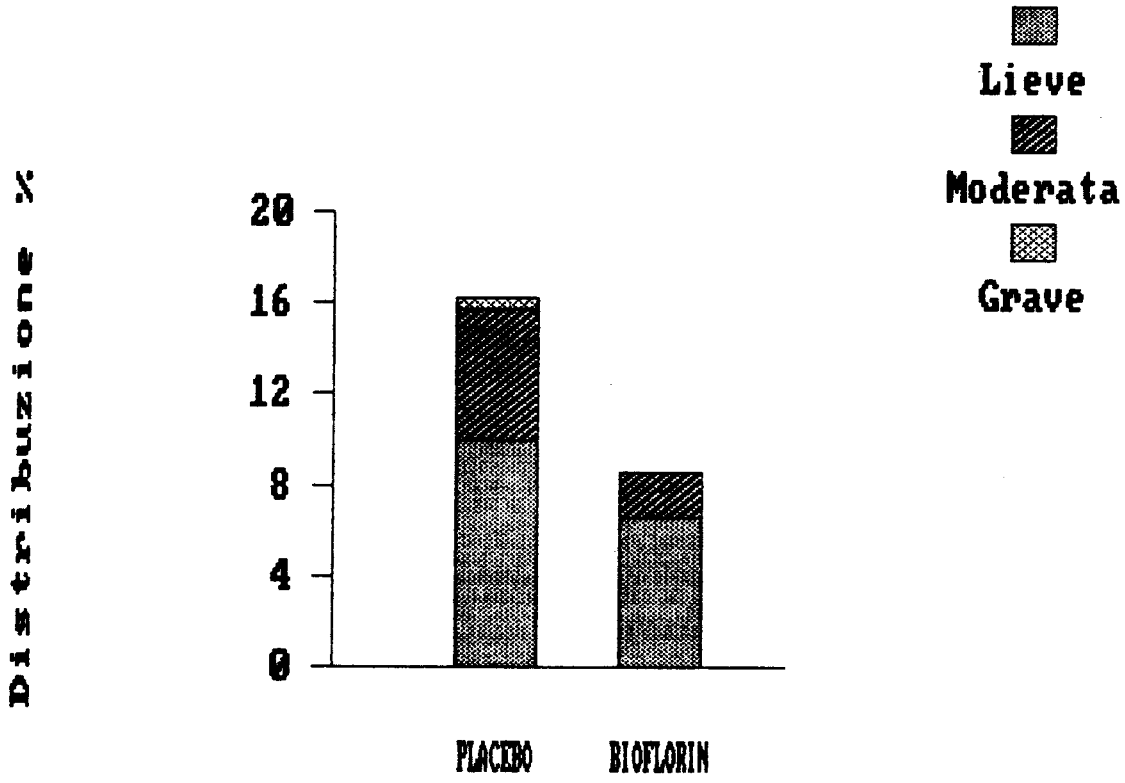

FIGURA 5 - Studio CONTROLLATO DOPPIO-CIECO di tipo PREVENTIVO Giorno di comparsa della diarrea durante terapia antibiotica nei due gruppi a confronto

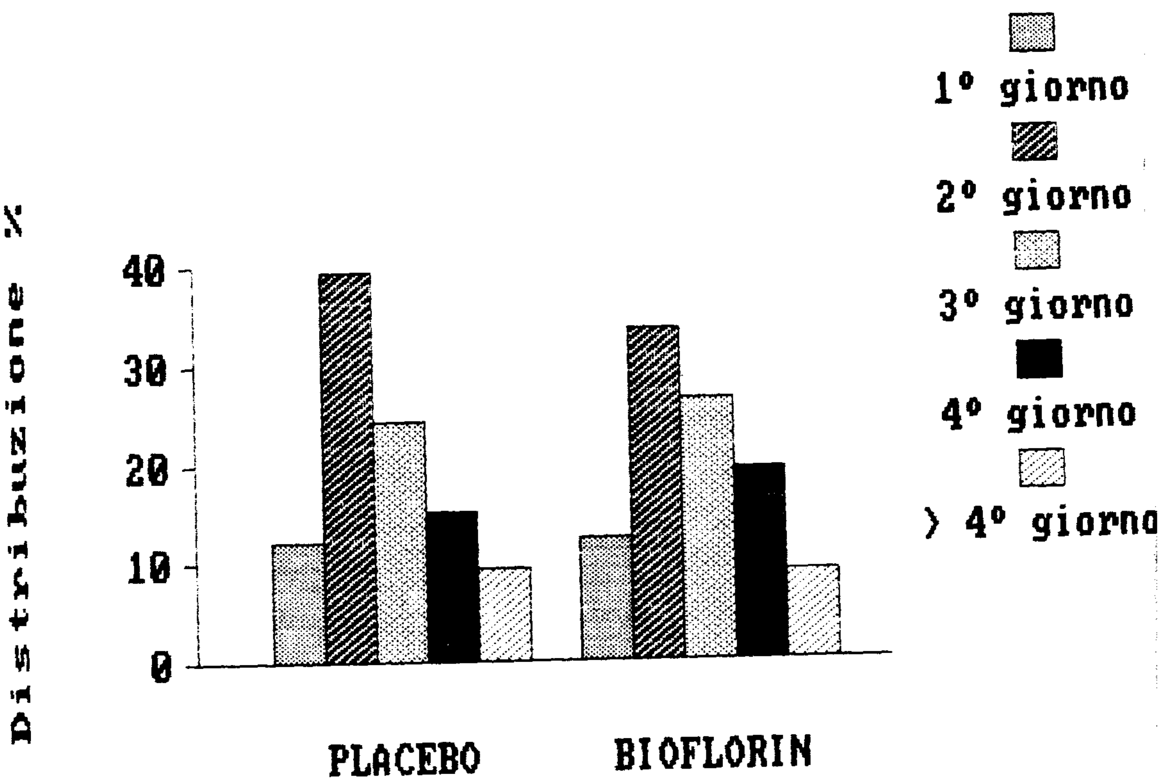

FIGURA 6 - Studio CONTROLLATO DOPPIO-CIECO di tipo PREVENTIVO
Durata della diarrea (giorni): confronto PLACEBO - BIOFLORIN

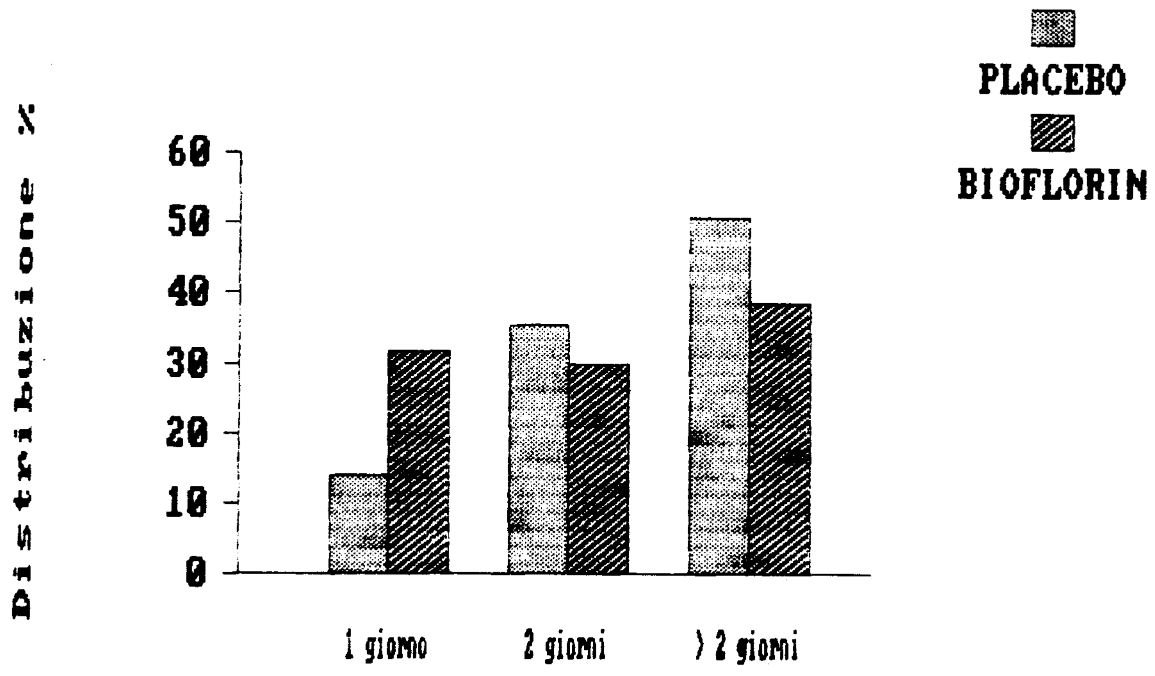

FIGURA 7 - Studio CONTROLLATO DOPPIO-CIECO di tipo PREVENTIVO
Grado e frequenza di comparsa della diarrea durante il trattamento con i diversi tipi di antibiotici: confronto PLACEBO (P) - BIOFLORIN (B)

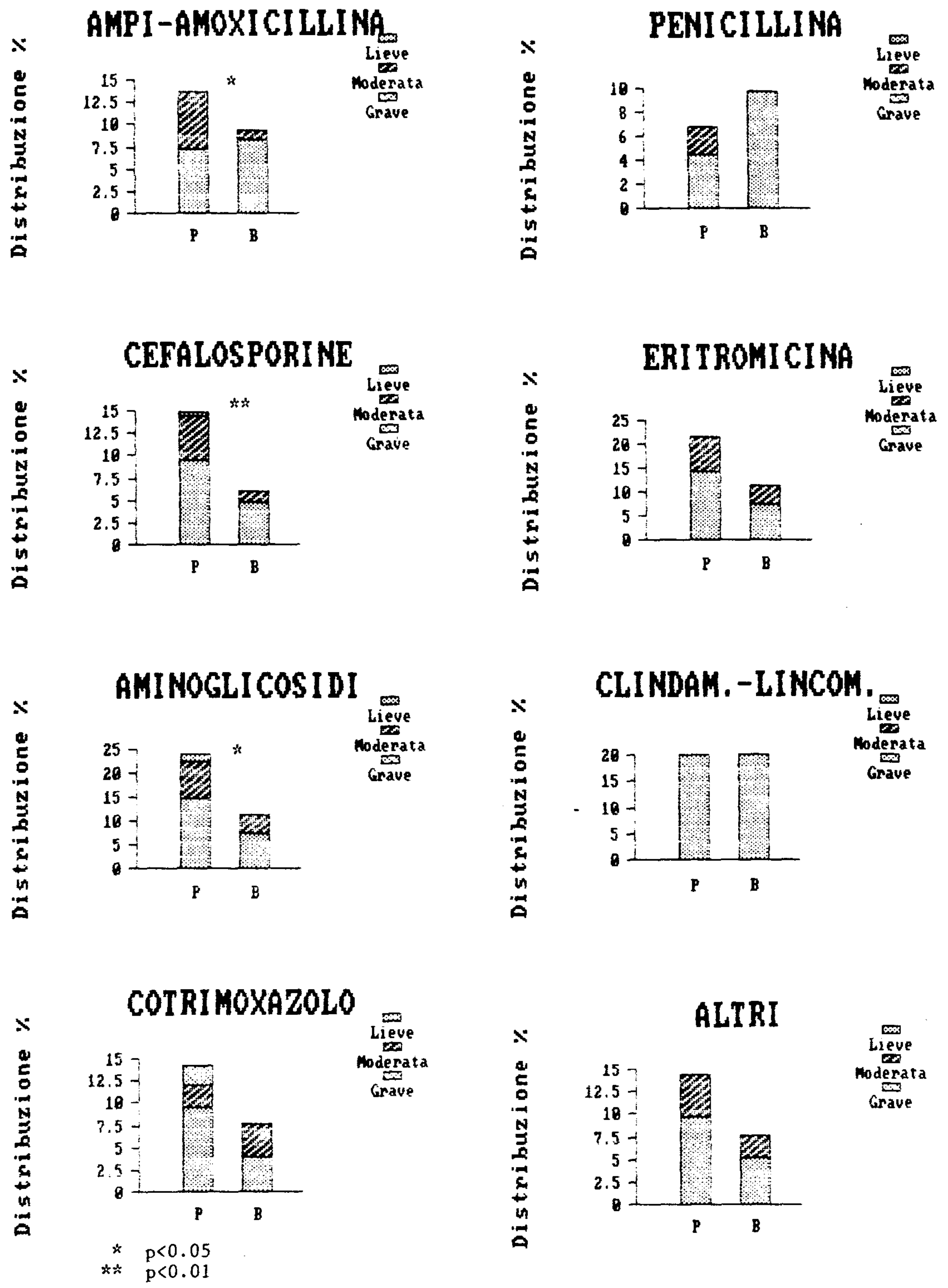

FIGURA 8 - Studio CONTROLLATO DOPPIO-CIECO di tipo PREVENTIVO
Grado e frequenza di comparsa della diarrea nei gruppi PLACEBO - BIOFLORIN durante terapia antibiotica: confronto tra la via di somministrazione orale (O) e parenterale (P)

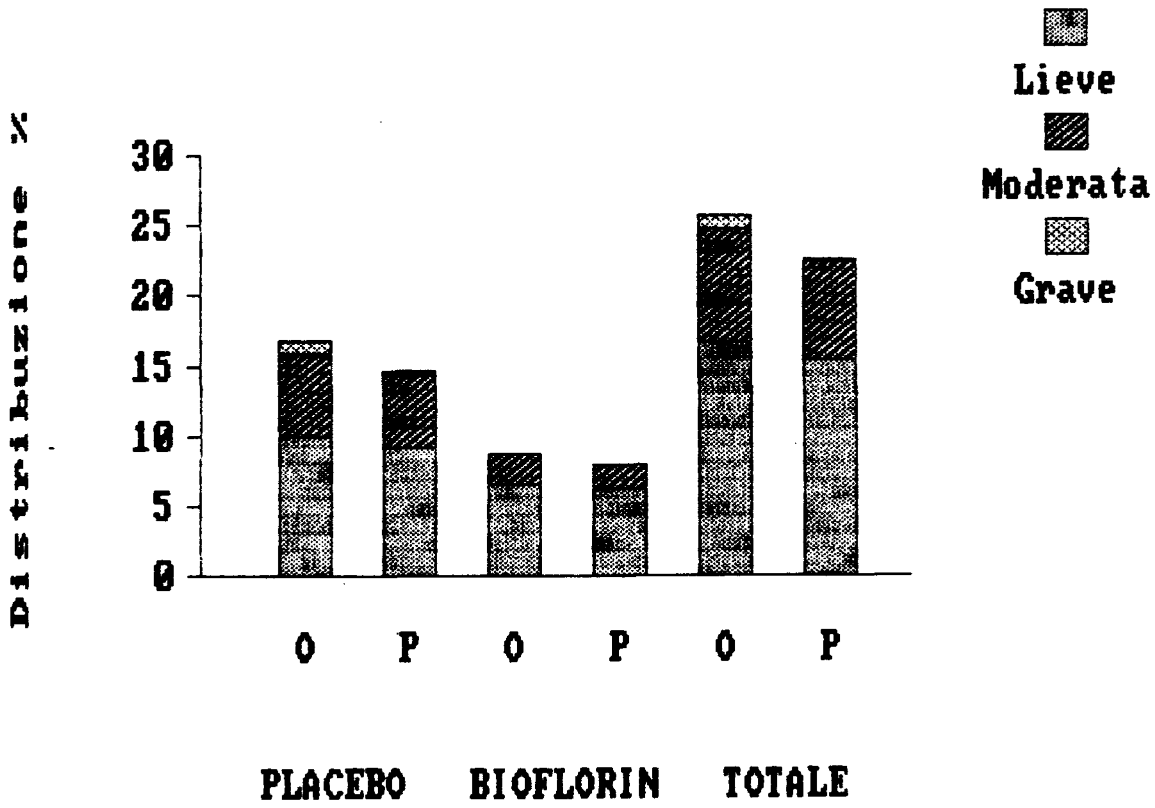

FIGURA 9 - Studio CONTROLLATO DOPPIO-CIECO di tipo PREVENTIVO
Frequenza di comparsa di glossiti e ragadi durante terapia antibiotica: confronto PLACEBO - BIOFLORIN

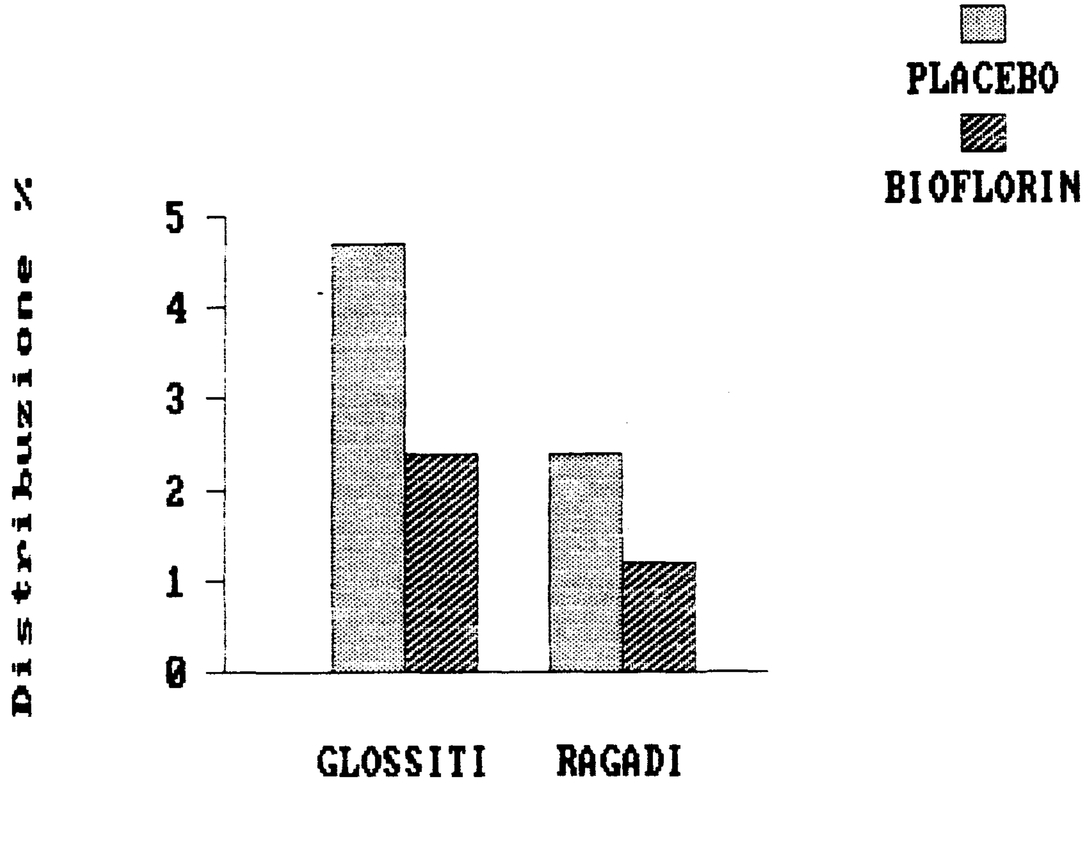

FIGURA 10 - Studio APERTO di tipo PREVENTIVO
Distribuzione per età (anni)

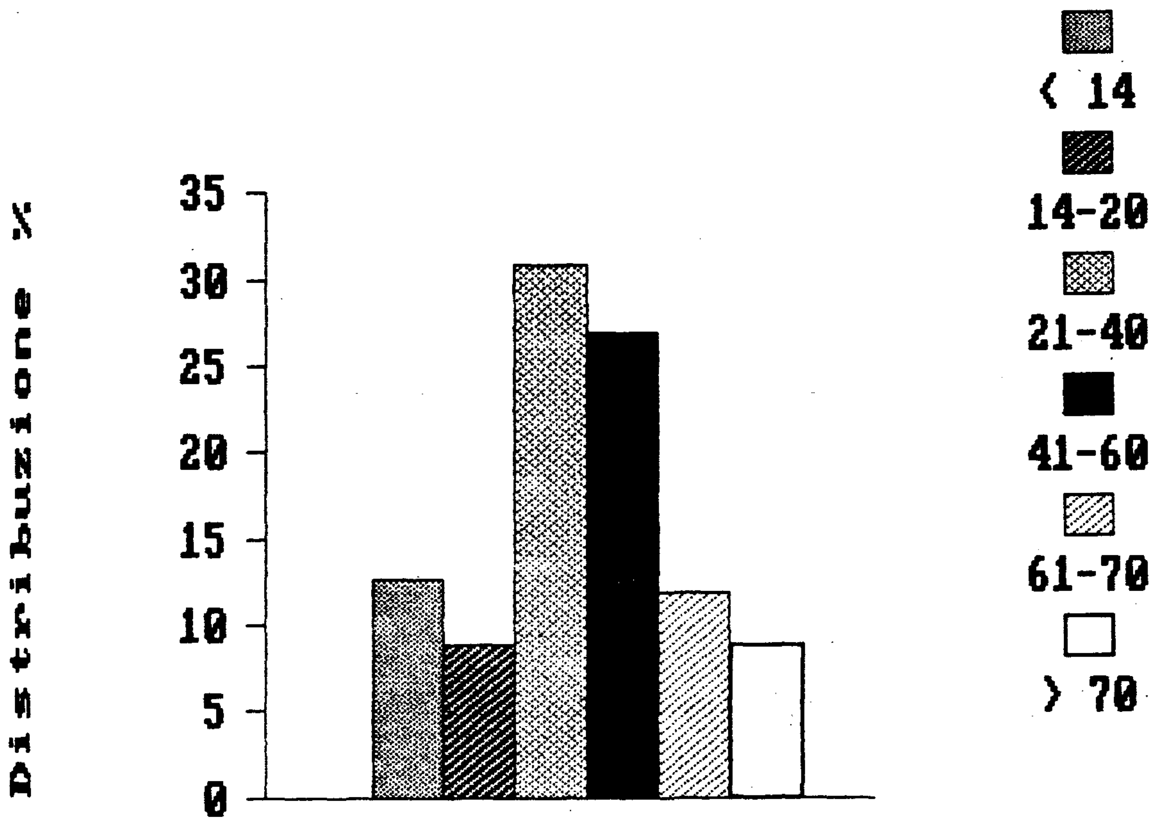

FIGURA 11 - Studio APERTO di tipo PREVENTIVO
Dati basali - Antibiotici adottati

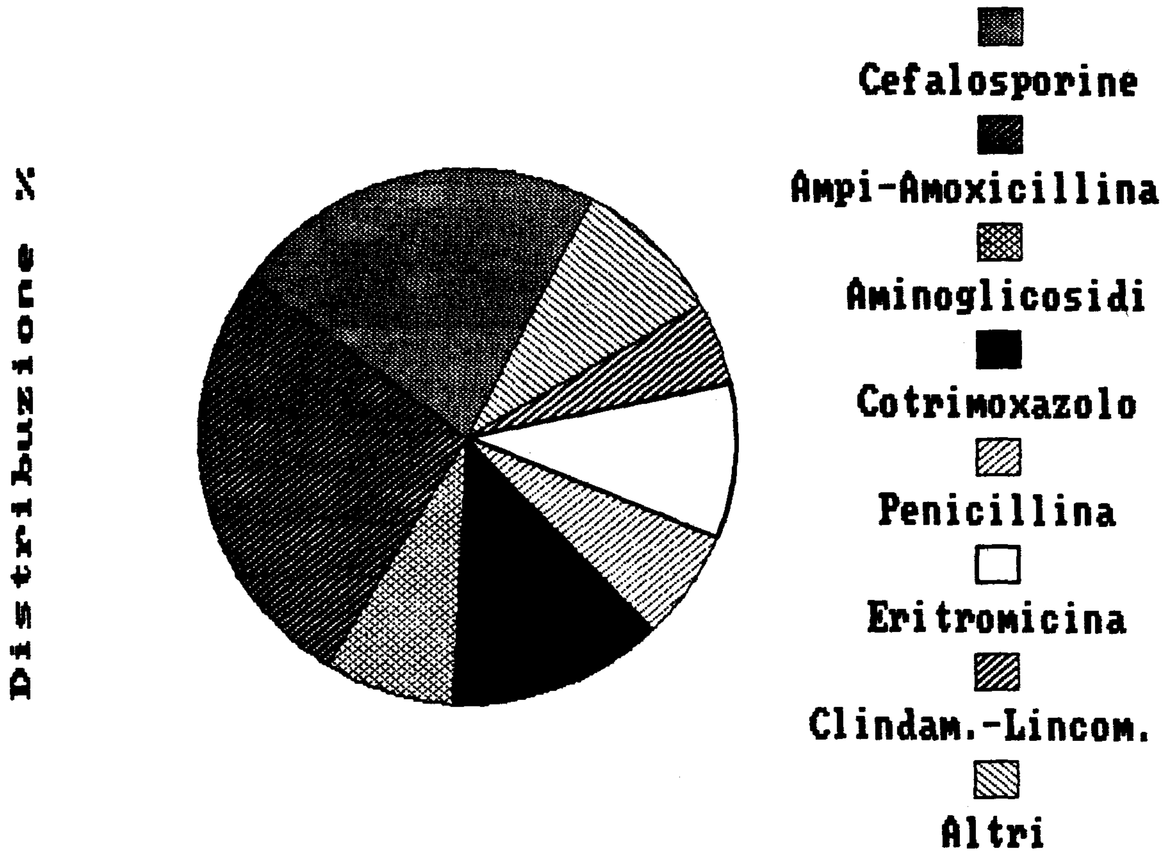

FIGURA 12 - Studio APERTO di tipo PREVENTIVO
Frequenza e gravità della reazione diarroica

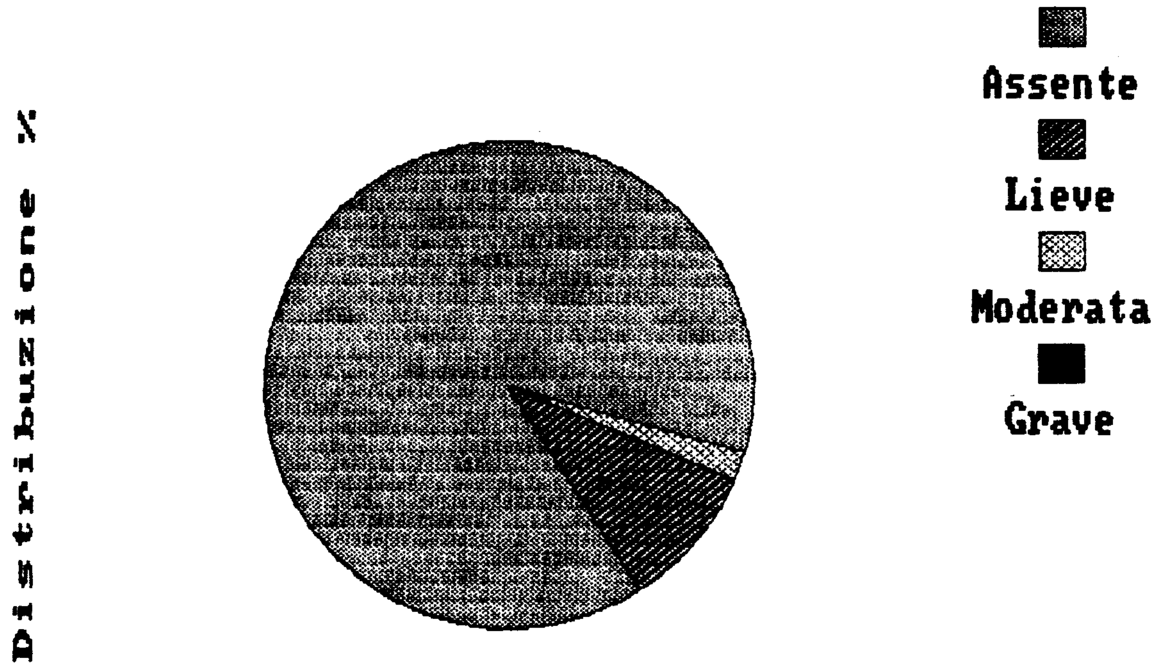

FIGURA 13 --Studio APERTO di tipo PREVENTIVO
Frequenza di comparsa ed entità della diarrea durante trattamento con i differenti antibiotici

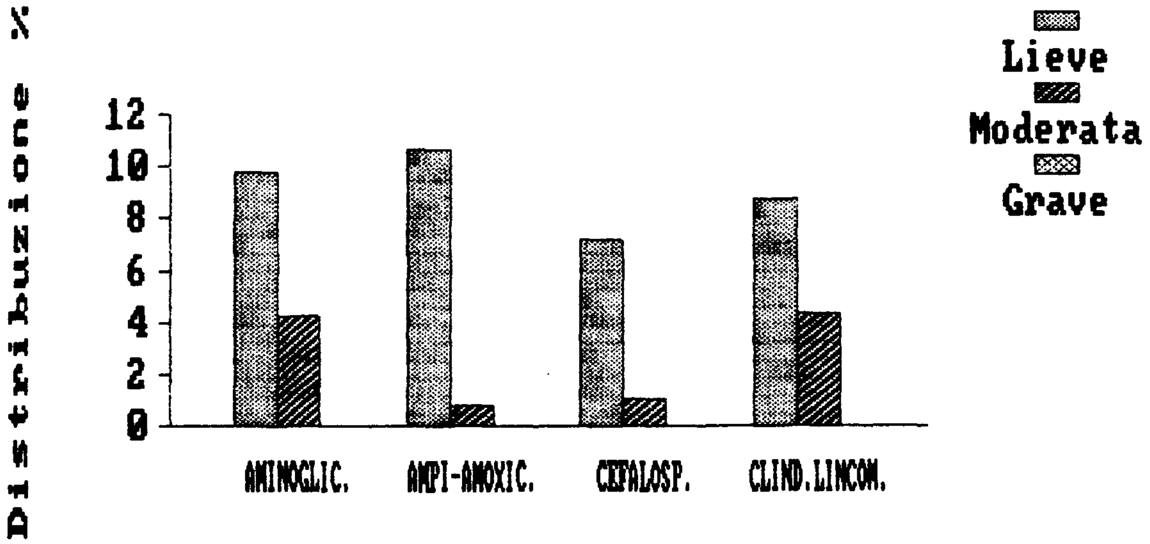

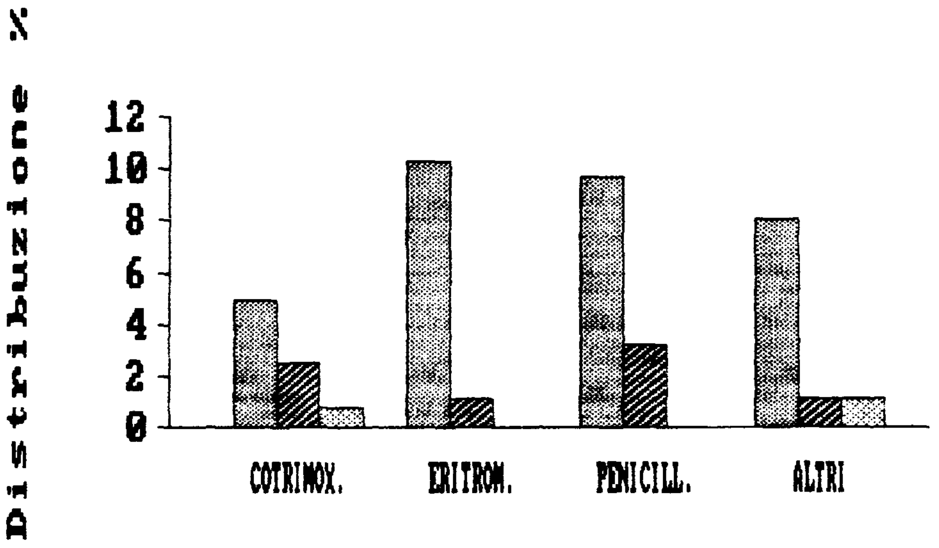

FIGURA 14 - Studio APERTO di tipo PREVENTIVO
Giorno di comparsa della diarrea

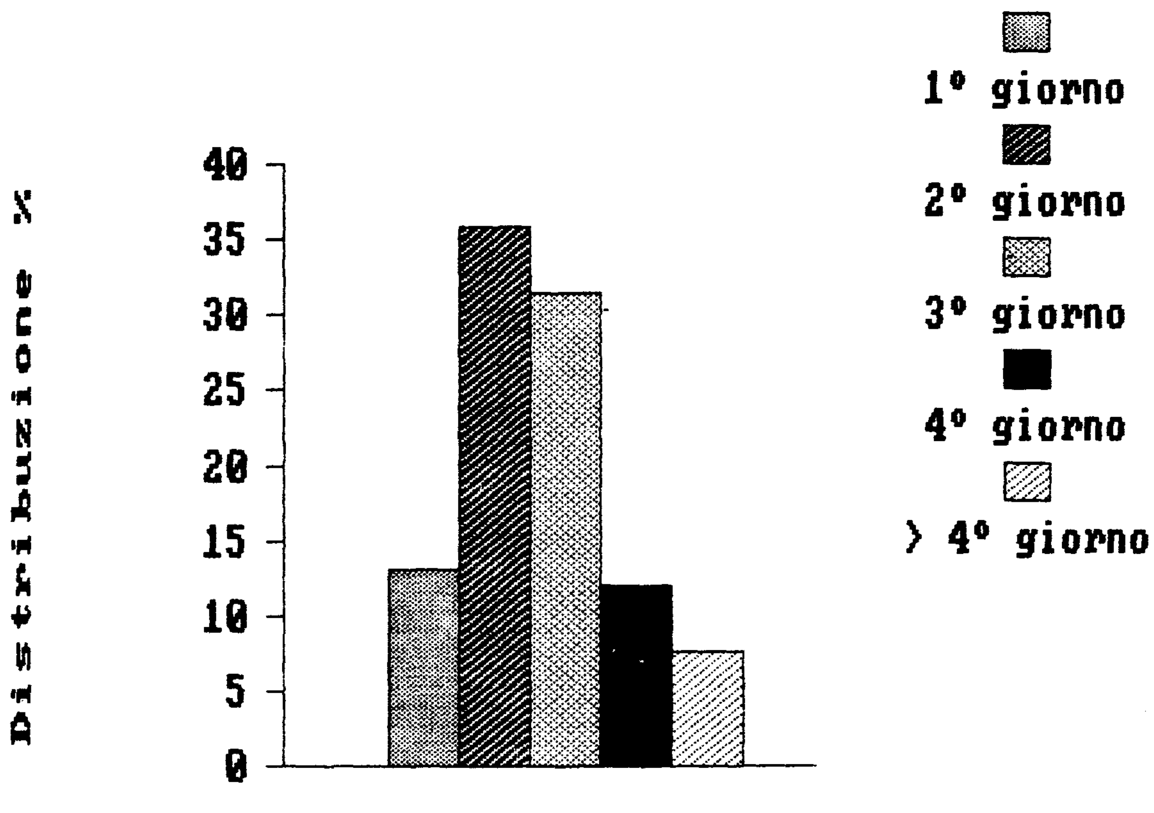

FIGURA 15 - Studio APERTO di tipo PREVENTIVO
Durata della diarrea (giorni)

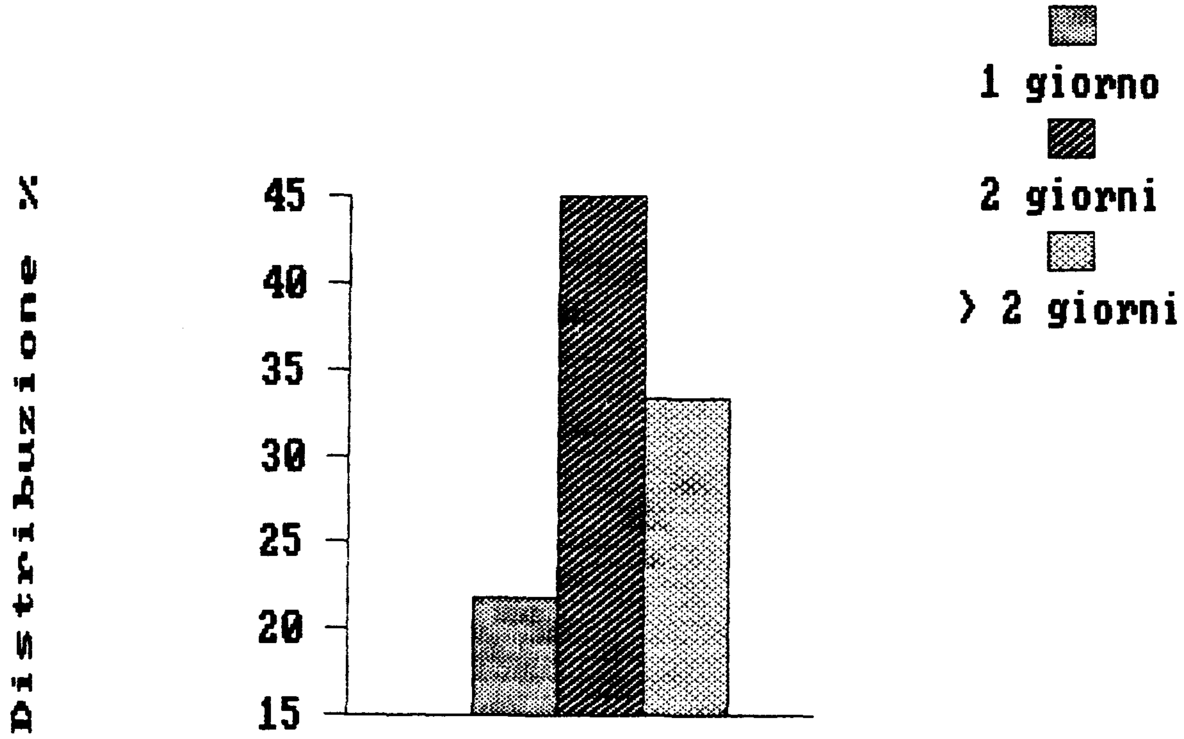

FIGURA 16 - Studio CONTROLLATO DOPPIO-CIECO di tipo TERAPEUTICO
Distribuzione per età (anni) nei due gruppi di pazienti

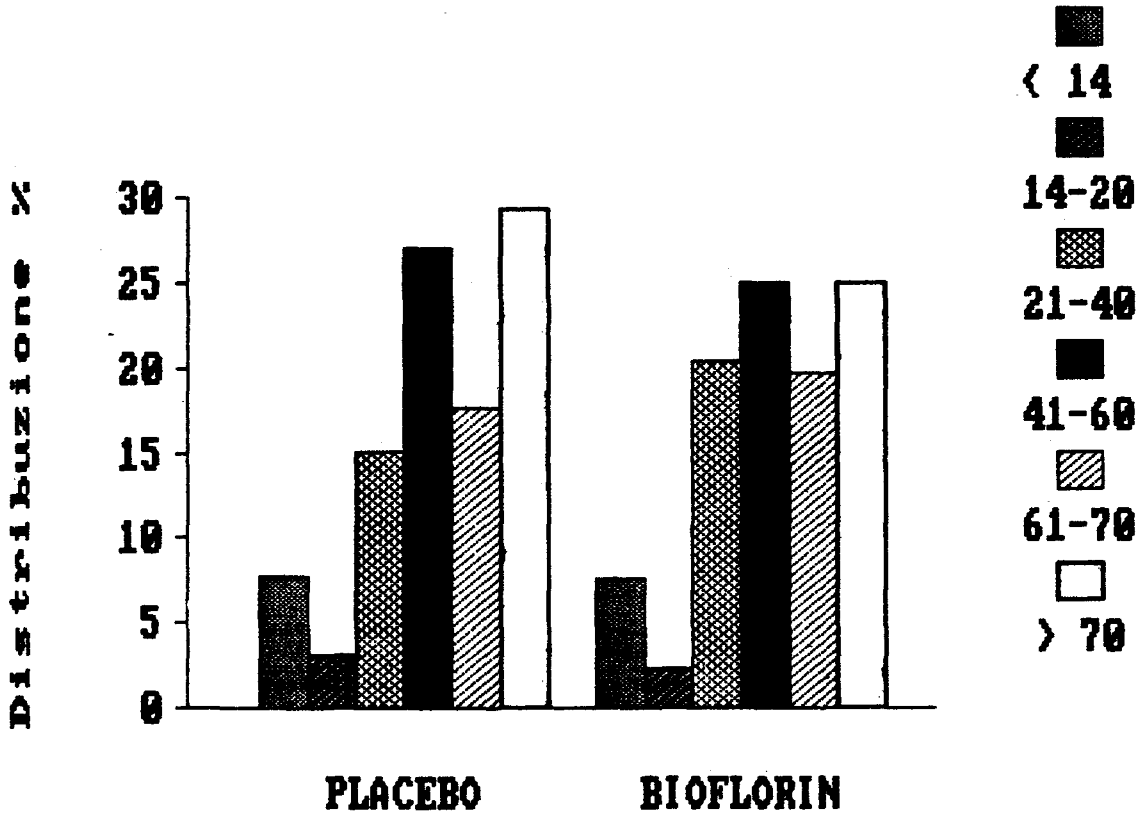

FIGURA 17 - Studio CONTROLLATO DOPPIO-CIECO di tipo TERAPEUTICO
Distribuzione per eziopatogenesi nei due gruppi

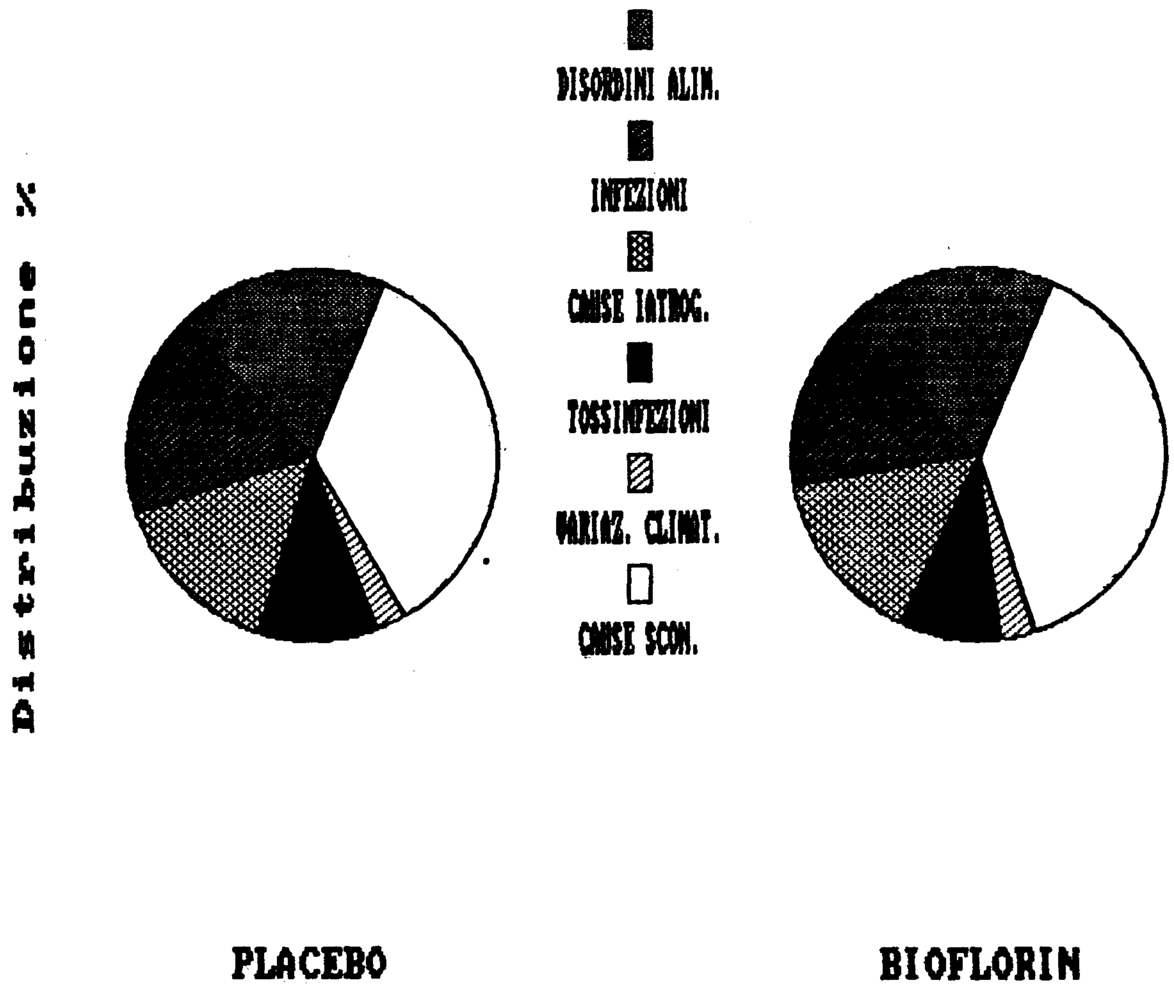

FIGURA 18 - Studio CONTROLLATO DOPPIO-CIECO di tipo TERAPEUTICO Frequenza dell'alvo (no. scariche/die) basale nei due gruppi di pazienti

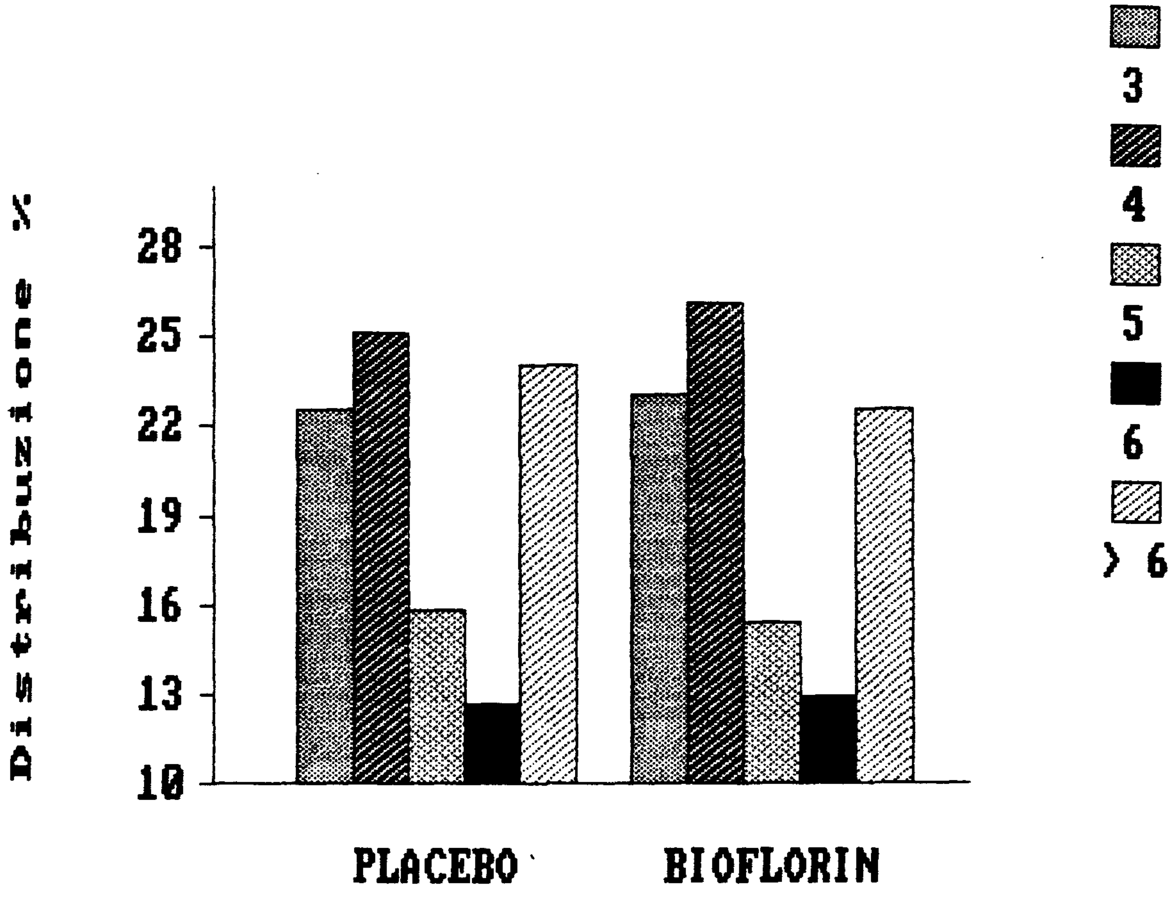

FIGURA 19 - Studio CONTROLLATO DOPPIO-CIECO di tipo TERAPEUTICO
Sintomatologia basale nei due gruppi

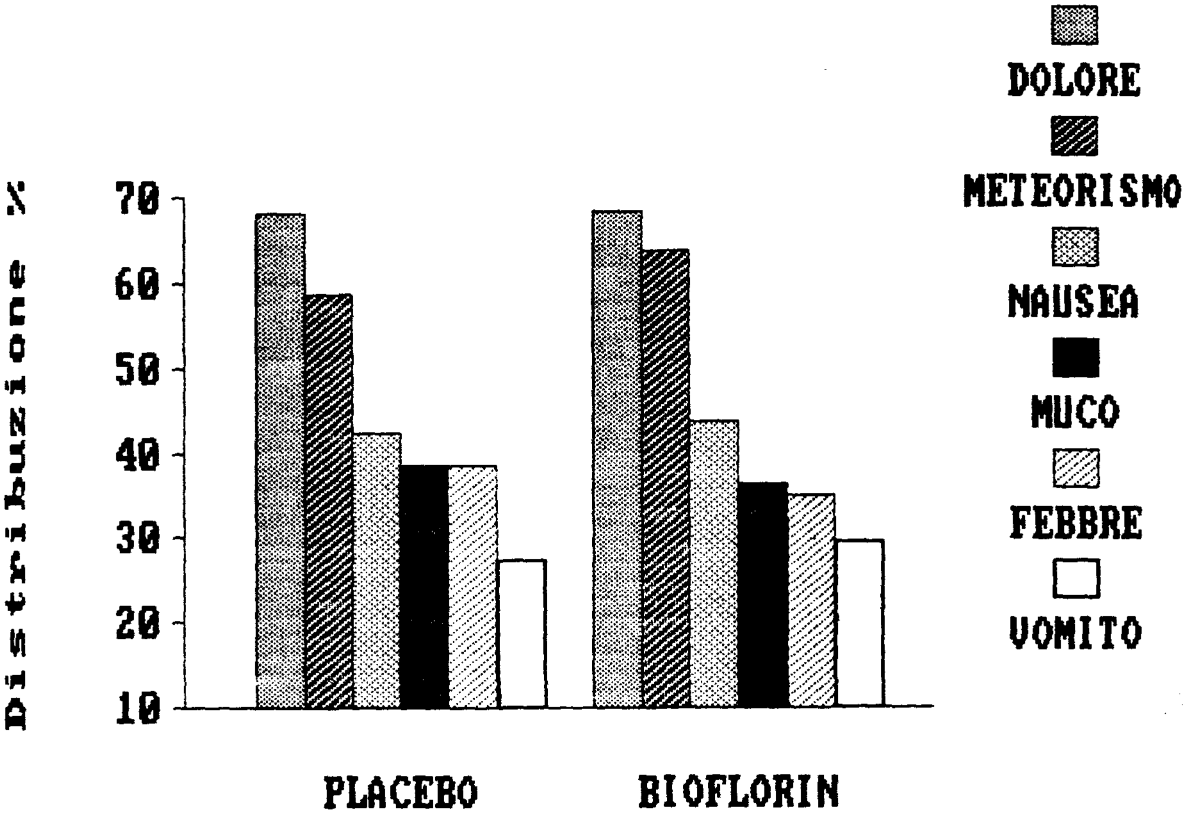

FIGURA 20 - Studio CONTROLLATO DOPPIO-CIECO di tipo TERAPEUTICO
Curve di persistenza di una frequenza dell'alvo con 3 o più scariche/die

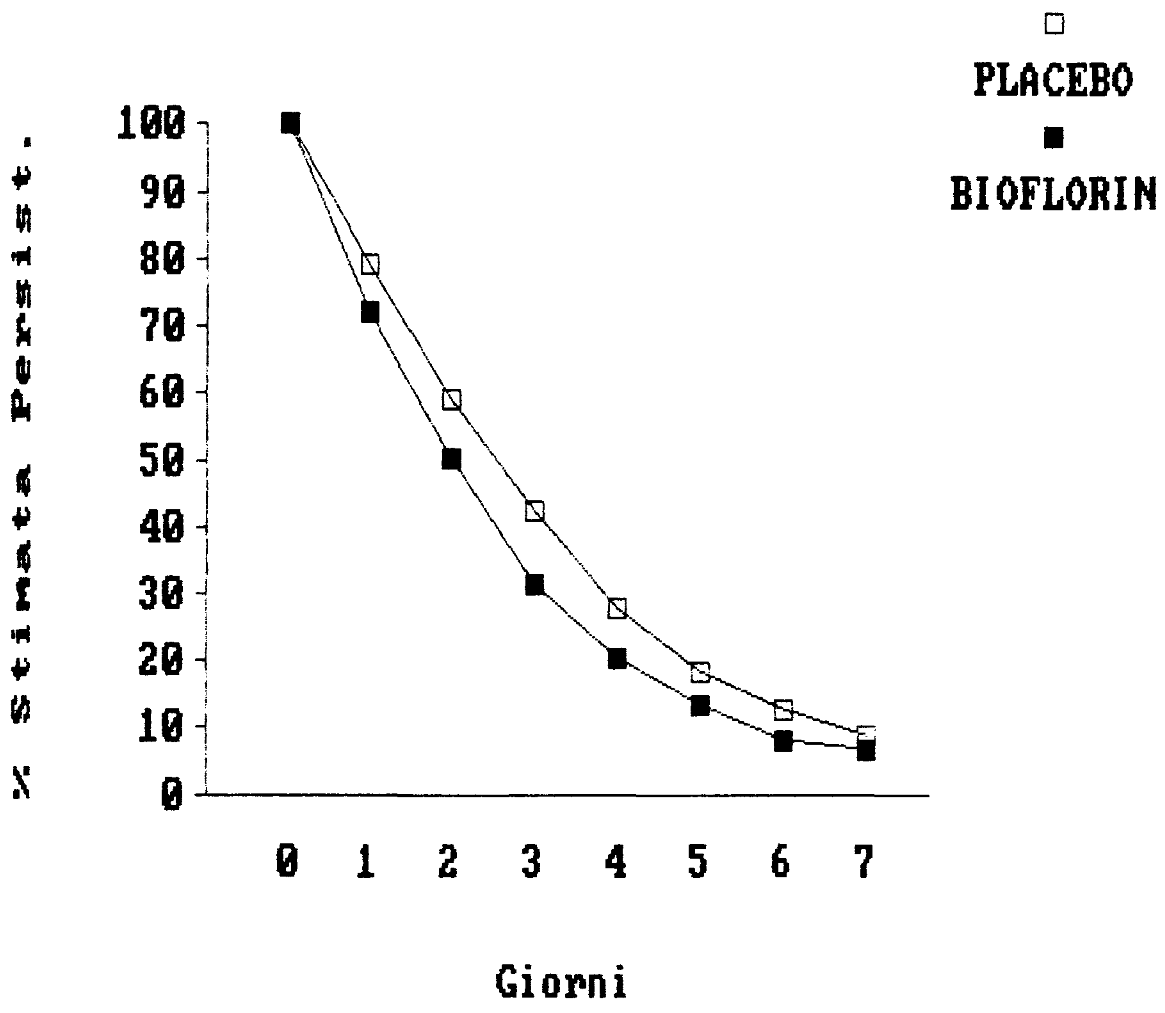

FIGURA 21 - Studio CONTROLLATO DOPPIO-CIECO di tipo TERAPEUTICO
Curve di persistenza di feci liquide o semiliquide

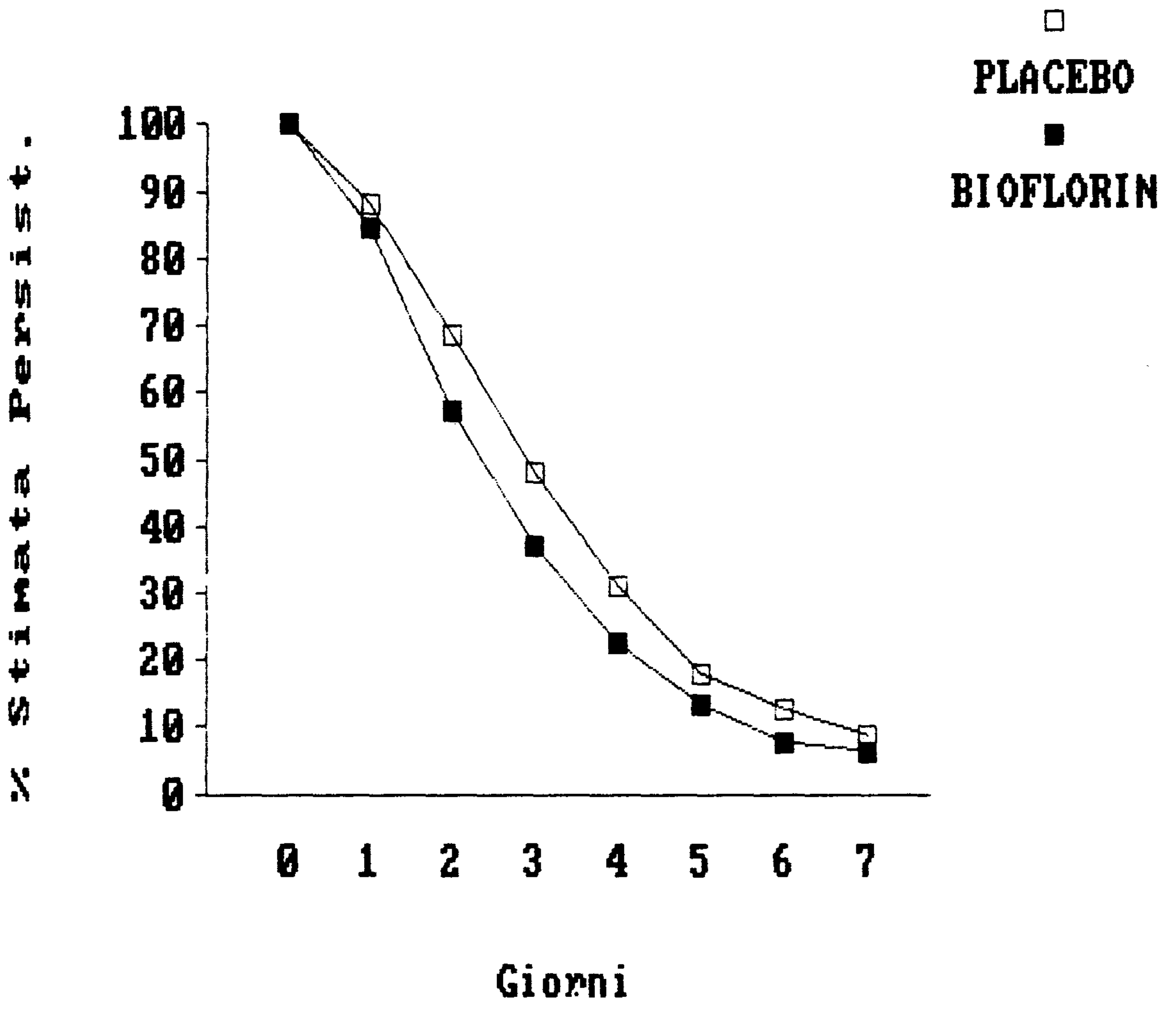

FIGURA 22 - Studio CONTROLLATO DOPPIO-CIECO di tipo TERAPEUTICO
Curve di persistenza di alvo diarroico (valutazione combinata dei caratteri frequenza dell'alvo e consistenza delle feci)

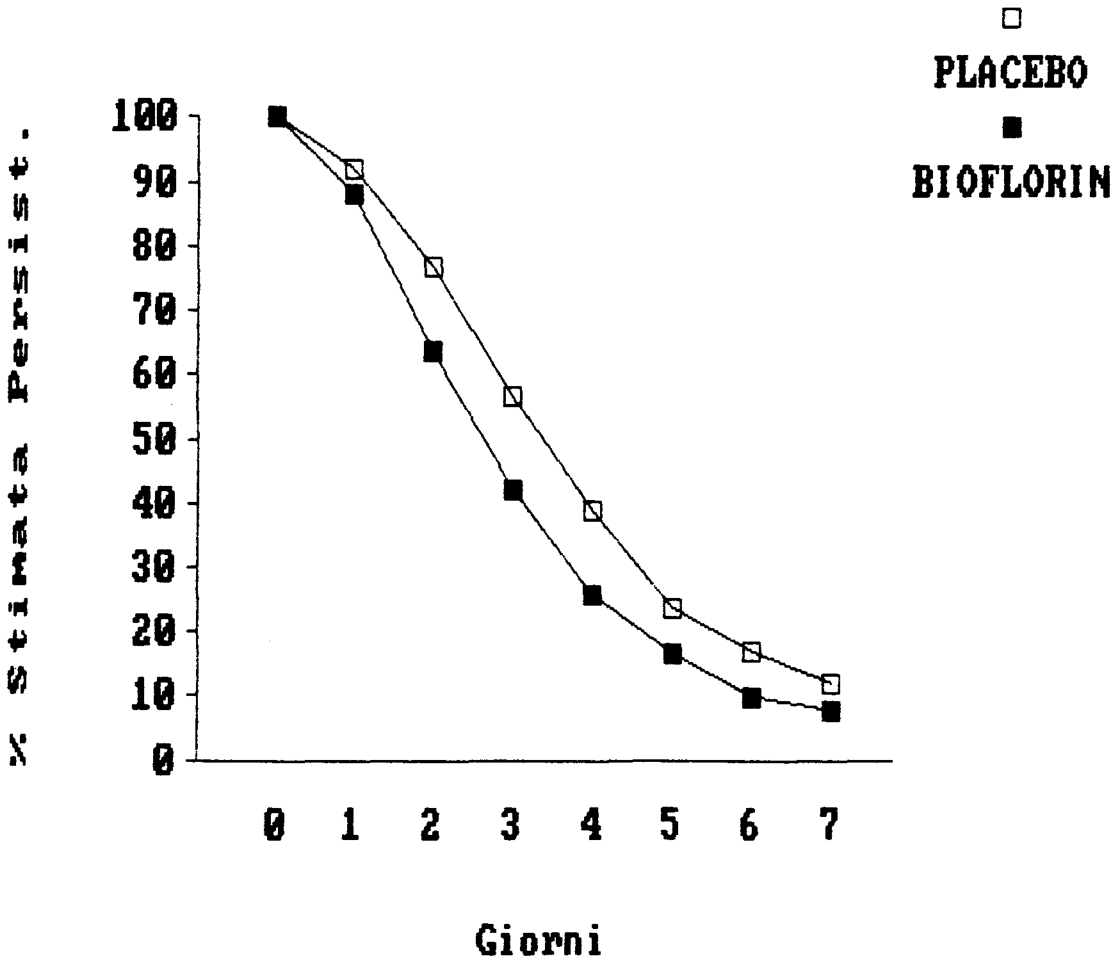

FIGURA 23 - Studio CONTROLLATO DOPPIO-CIECO di tipo TERAPEUTICO
Curve di persistenza di muco nelle feci

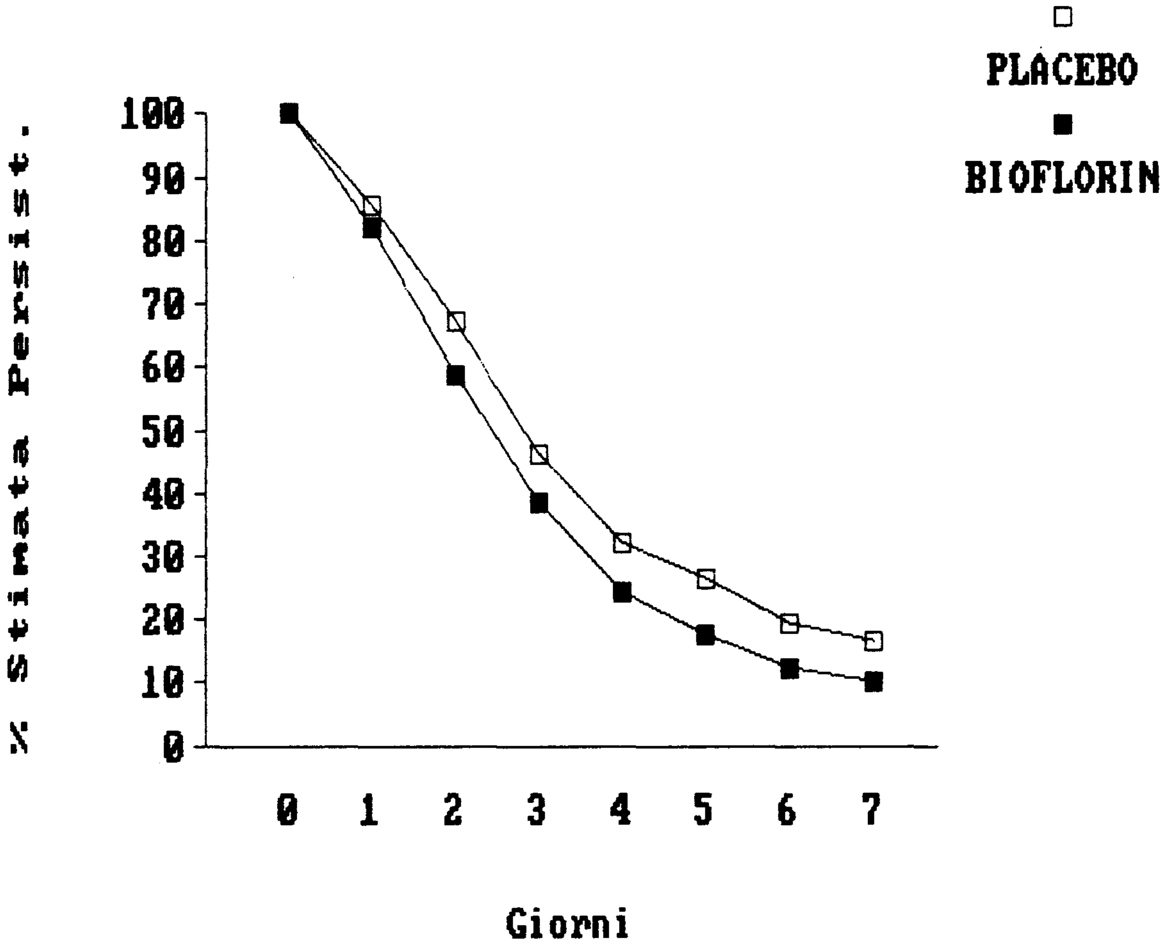

FIGURA 24 - Studio CONTROLLATO DOPPIO-CIECO di tipo TERAPEUTICO
Curve di persistenza di dolore addominale

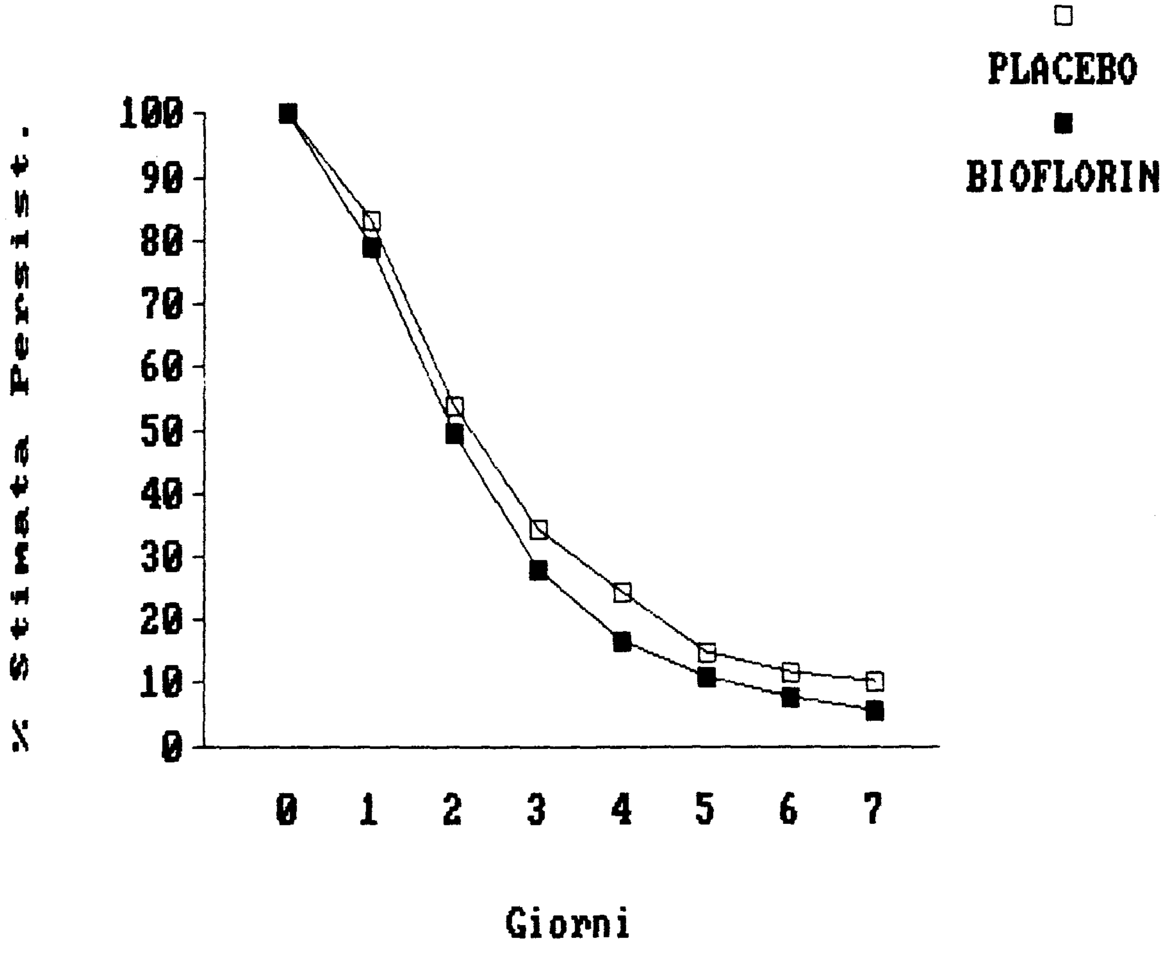

FIGURA 25 - Studio APERTO di tipo TERAPEUTICO
Distribuzione per età (anni)

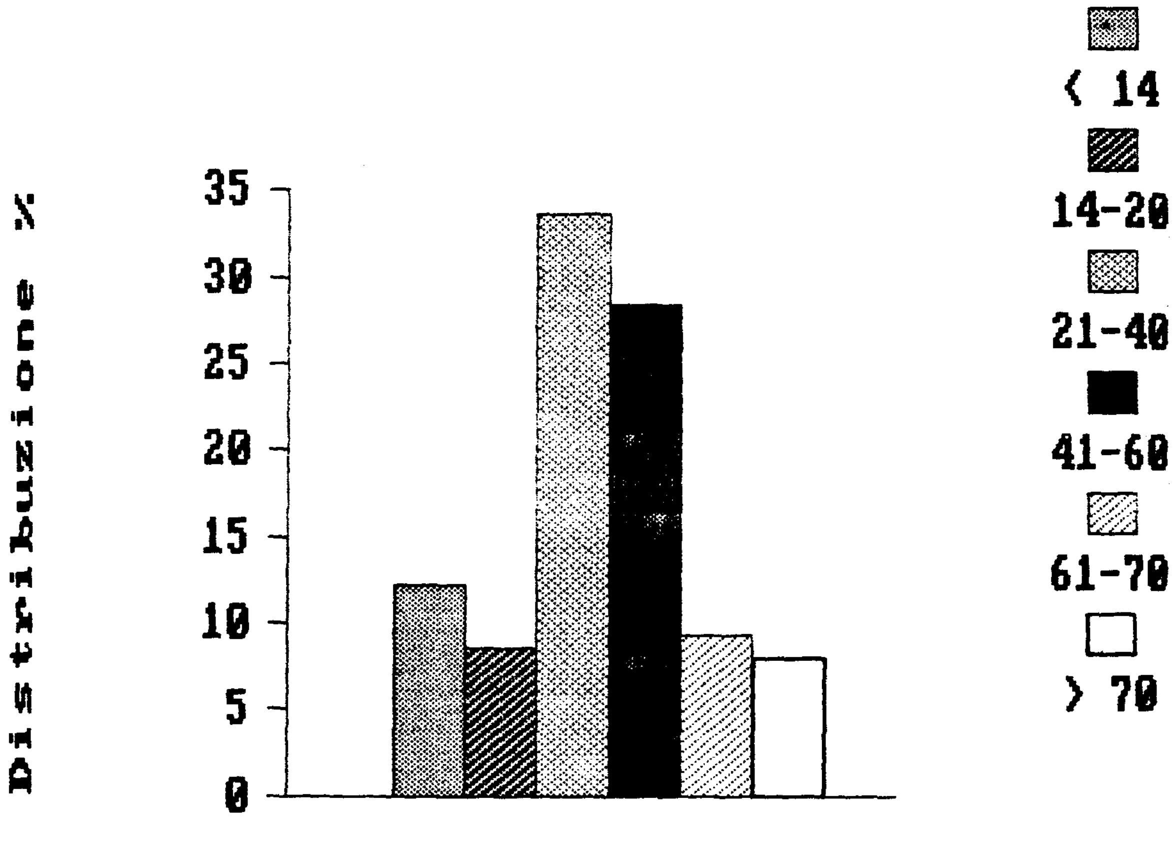

FIGURA 26 - Studio APERTO di tipo TERAPEUTICO
Distribuzione della forma diarroica per eziopatogenesi

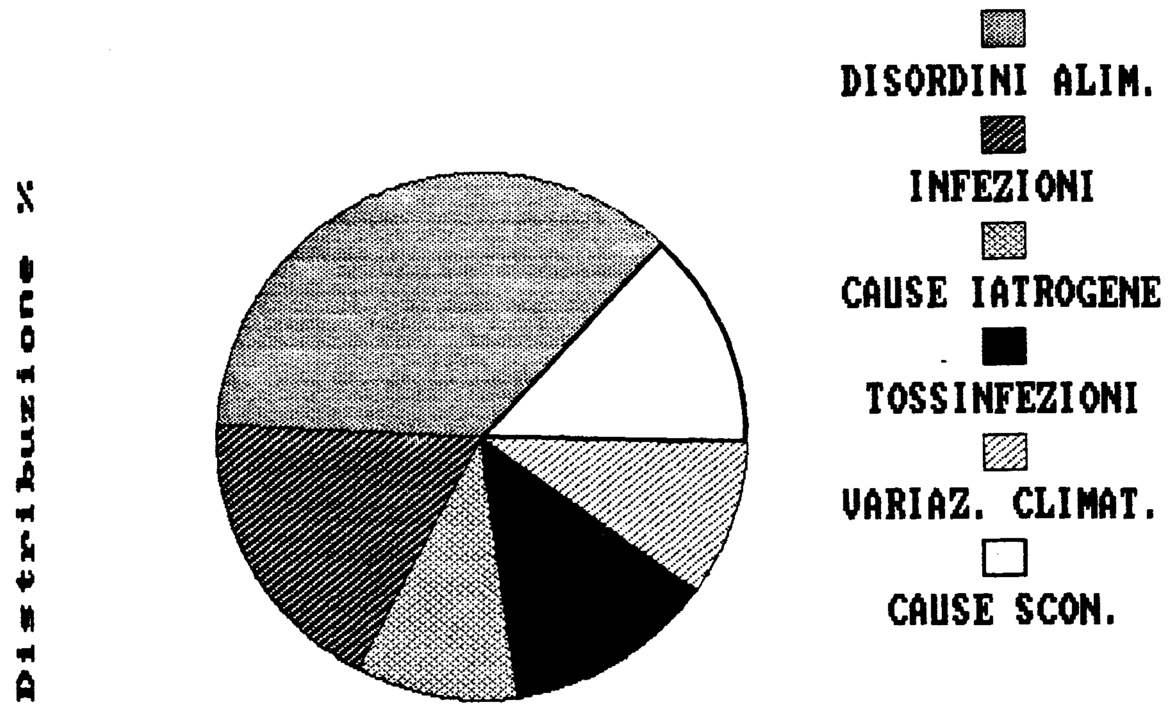

FIGURA 27 - Studio APERTO di tipo TERAPEUTICO
Frequenza basale dell'alvo (no. scariche/die)

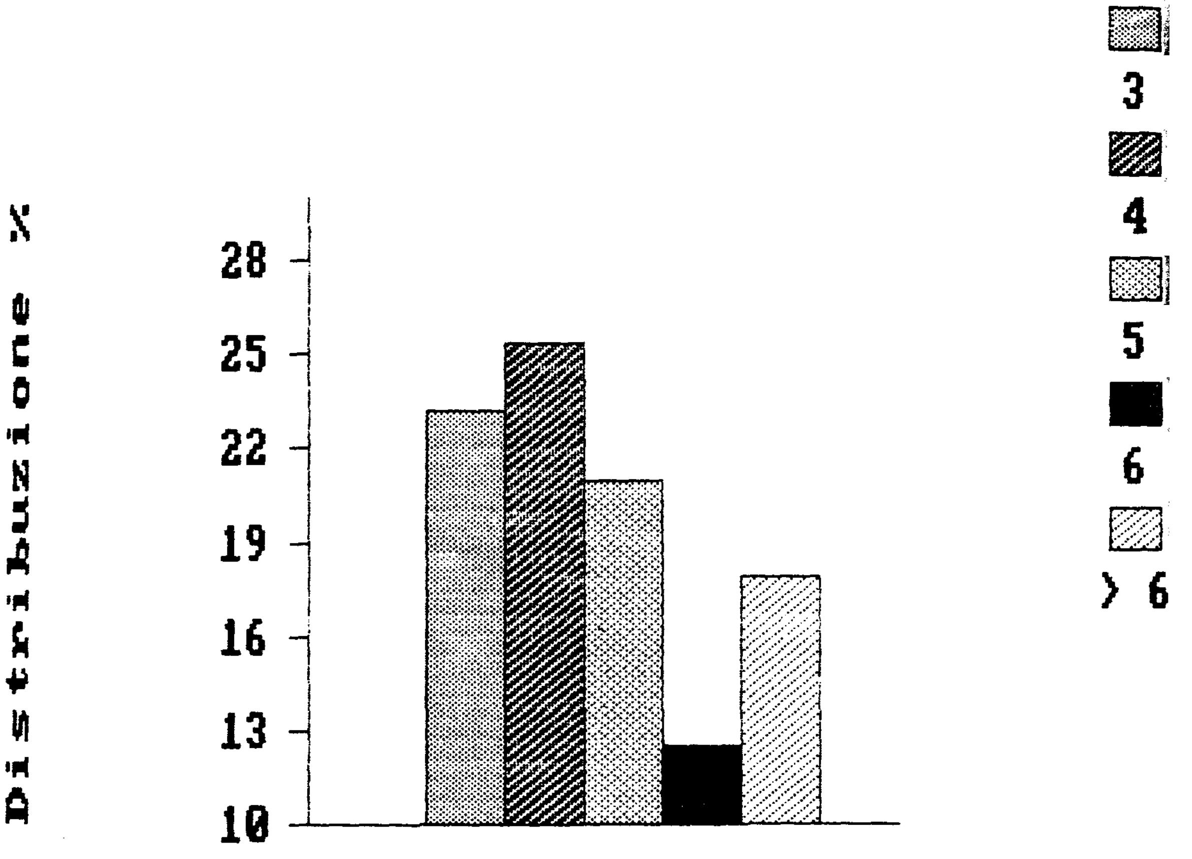

FIGURA 28 - Studio APERTO di tipo TERAPEUTICO
Sintomatologia basale

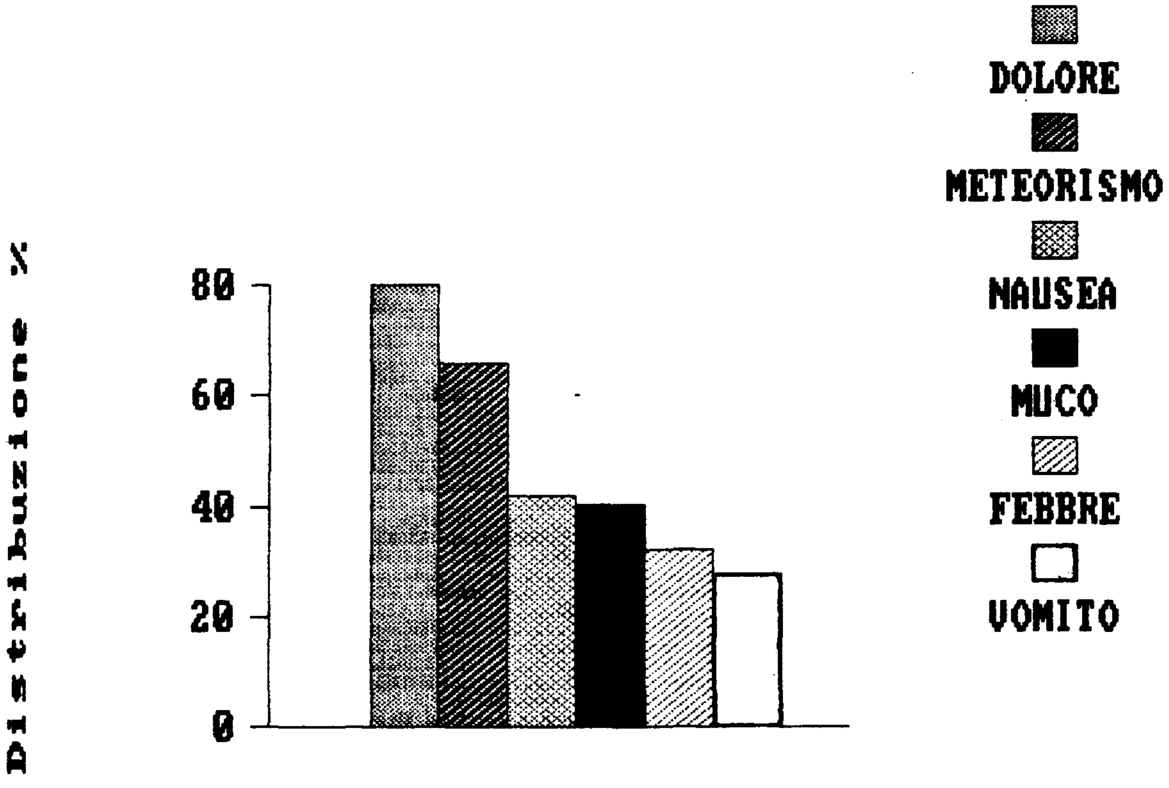

FIGURA 29 - Studio APERTO di tipo TERAPEUTICO
Curve di persistenza dei parametri alterati (per confronto vengono riportate anche le curve riguardanti lo studio CONTROLLATO DOPPIO-CIECO)

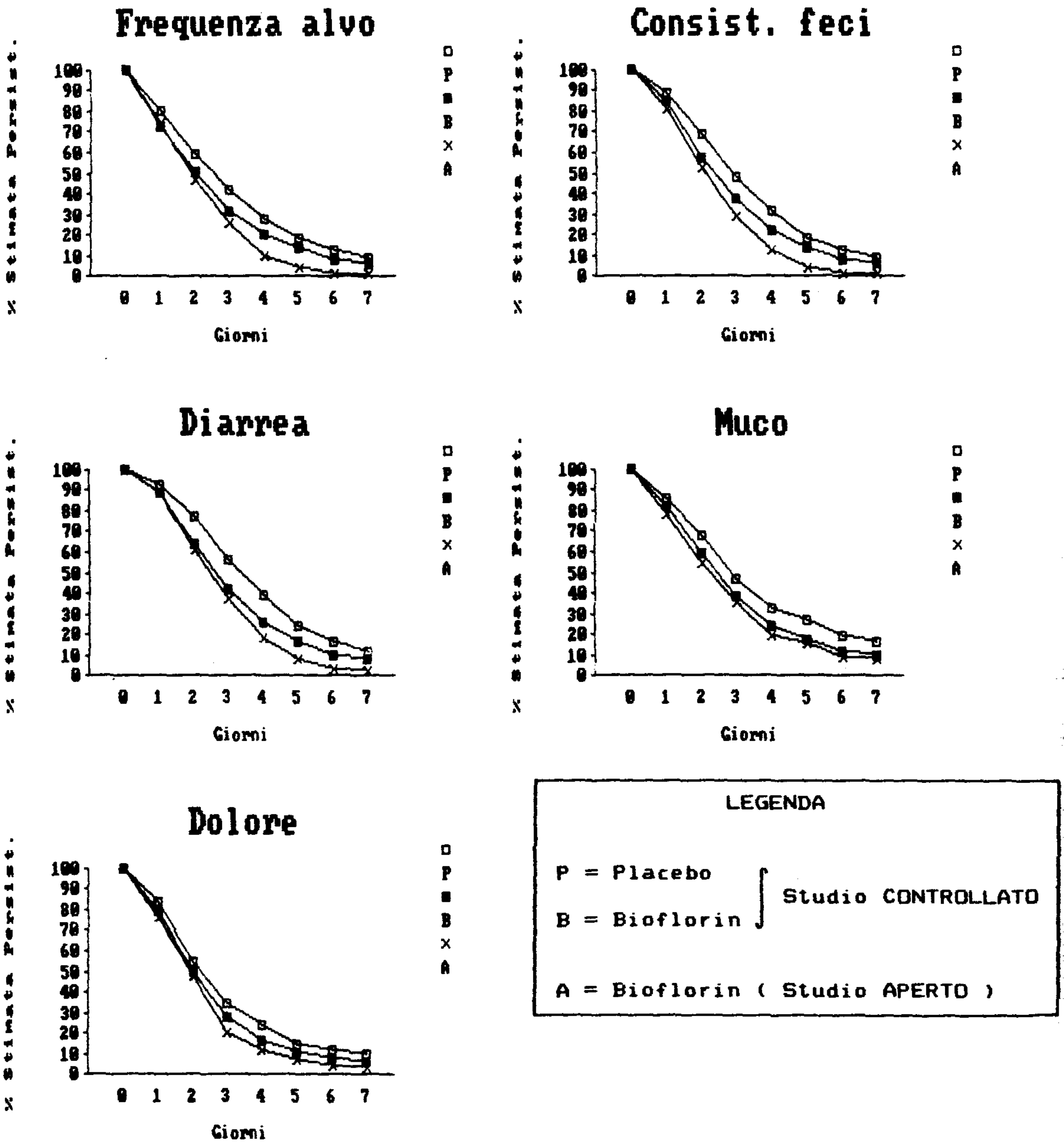

BIBLIOGRAFIA

1) Gotz V.P., Rand K.H.
Medical management of antimicrobial-associated diarrhea and colitis
Pharmacotherapy 2, 100-109, 1982

2) Editorial
Antibiotic-associated colitis - the continuing saga
Br.Med.J. 282, 1913-1914, 1981

3) Abrams G.D., Bishop J.E.
Effect of the normal microbial flora on the resistance of the small intestine to infection
J.Bacteriol. 92, 1604-1608, 1966

4) Bohnhoff M., Miller C.P.
Enhanced susceptibility to Salmonella infection in streptomicin-treated mice
J.Infect.Dis 111, 117-127, 1962

5) George W.L., Rolfe R.D., Finegold S.M.
Treatment and prevention of antimicrobial agent-induced colitis and diarrhea
Gastroenterology 79, 366-372, 1980

6) Candy D.C.A.
Diarrhoea dehydratation and drugs
Br.Med.J. 289, 1245-1246, 1984

7) Camarri E., Belvisi A., Guidoni G., Marini G., Frigerio G.
A double-blind comparison of two different treatments for acute enteritis in adults
Chemotherapy 27, 466-470, 1981

8) Bruno F., Frigerio G.
Eine neuartige Möglichkeit zur Behandlung der Enteritis-Kontrollierte Doppel- blindversuche mit dem Stamm SF 68
Schweiz.Rundschau Med.(PRAXIS) 70, 1717-20, 1981

9) Alvisi V., Tralli M., Loponte A., Pamani F., Massari M.
Studio in doppio cieco sul trattamento con SF 68 o con antibiotici nelle enteriti acute dell'adulto
Clin.Ter. 101, 581-586, 1982

10) Bruno F., Nastasi A., Bruno M.
Studio controllato doppio-cieco sull'effetto dell'enterococco lattoproduttore ceppo SF 68 su manifestazioni associate a forme enterocolitiche varie e a salmonellosi
Clin.Ter. 105, 203-207, 1983

11) Bellomo G., Mangiagli A., Nicastro L., Frigerio G.
A controlled double-blind study of SF 68 strain as a new biological preparation for the treatment of diarrhoea in pediatrics
Curr.Ther.Res. 28, 927-936, 1980

12) Bellomo G., Finocchiaro C., Frigerio G., Mangiagli A., Nicastro L.
Studio controllato sull'enterococco LAB ceppo SF 68 nelle enteriti acute del bambino concomitanti ad infezioni delle vie respiratorie
Clin.Pediatrica 64, 219-227, 1982

13) D'Apuzzo V., Salzberg R.
Die Behandlung der akuten Diarrhö in der Pädiatrie mit streptococcus faecium: Resultate einer Doppelblinstudie
Therapeutische Umschau 39, 1033-1035, 1982

14) Bellomo G., Finocchiaro C., Frigerio G.
Une nouvelle approche pour le traitement des entérites en pédiatrie
Méd et Hyg. 37, 3781-3784, 1979

15) Borgia M., Sepe N., Brancato V., Borgia R.
A controlled clinical study on Streptococcus faecium preparation for the prevention of side reactions during long-term antibiotic treatments
Curr.Ther.Res. 31, 265-271, 1982

16) Lewenstein A., Frigerio G., Moroni M.
Biological properties of SF 68, a new approach for the treatment of diarrheal diseases
Curr.Ther.Res. 26, 967-981, 1979

17) Carbone M., Bonina L., Fera M.T.
Microbiological properties of Streptococcus faecium SF 68 strain and its relationships with other microrganisms
Boll.Ist.Sieroter.Milanese 59, 591-598, 1980

18) Bongetta R., Quirino T., Ortisi G., Privitera G., Foschi D., Cavagna G., Moroni M., Rovati V.
The colonization of Streptococcus faecium in human intestinal tract after oral administration
Boll.Ist.Sieroter.Milanese 60, 381-385, 1981

19) Ortisi O., Privitera G., Beniamino V., Moroni M.
Interference between SF 68 and Clostridium difficile possibly due to a bacteriocin
XIII International Congress of Chemotherapy, Vienna, 28th August-2nd September 1983

20) Kramer J., Brandis H.
Purification and characterization of two bacteriocins from Streptococcus faecium
Journal of General Microbiology 88, 93-100, 1975

21) Pugliese A.P., Galli M., Cosseta P., Ferrari A., Tovo P.A.
Streptococcus faecium SF 68 strain as interferon inducer in mice
XIII International Congress of Chemotherapy, Vienna, 28th August-2nd September, 1983

22) Iannello D., Bonina L., Delfino D., Berlinghieri M.C., Gismondo M.R., Mastroeni P.
Effect of oral administration of a variety of bacteria on depressed macrophage functions in tumor-bearing rats
Ann.Immunol.(Inst.Pasteur) 135C, 345-352, 1984

23) Bonina L., Focà A., Merendino R.
Attività in vivo ed in vitro di un estratto di Streptococcus faecium su Herpes simplex virus
Boll.Soc.Ital.Biol.Sper. 56, 2460-2466, 1980

24) Focà A., Bonina L.
Effects of Streptococcus faecium extract on Herpes simplex virus and picornaviridiae
Fems Symposium on microbial envelopes, Helsinki, 26th-30th May 1980

25) Armitage P.
Statistical methods in medical research
Blackwell Scientific Publications - Oxford 1971 (III edition)

THANKS

Special thanks go to Prof. Mauro Moroni, Director of the Institute of Infectious Diseases, University of Milan, for the support granted to the initiative, for his invaluable suggestions and direct participation in the project.
We also thank the external networks of Gipharmex S.p.A. of Milan (*) and of Bioresearch-Line RK of Milan (**) for having created operational contacts with the clinical centers and with all physicians who contributed to the research with their professional experience and enthusiasm; to all those doctors, considered as co-authors of the project, goes finally a special thanks for making the initiative concretely possible.

(*)

AMADEI C.	NOLI (SV)	LUGANI U.	GENOVA
CACEFFO C.	S.PIETRO IN CARIANO (VR)	RAMACCIOTTI F.	LIDO DI CAMAIORE (LU)
CALI' M.	MESSINA	RONCI B.	BOLOGNA
DE LUCIA M.	NAPOLI	SAIONI M.	ROMA
DE TOMASSI R.	FIRENZE	SASSI S.	SERIATE (BG)
FOGLIATO A.	MONCALIERI (TO)	TASSINARI C.	BUONACOMPRA (FE)
LUCCHIN R.	MOGLIANO VENETO (TV)	ZANOTTI G.	MILANO

(**)

ADDARI S.	PIRRI (CA)	LATELLA S.	MESSINA
AINIO A.	CAPUA (CE)	LAZZARO B.	ACI S.ANTONIO (CT)
ALTOMARE A.	BARI	LOMBARDO A.	BOLOGNA
AMAGLIANI F.	FALCONARA MARITTIMA (AN)	LUCCHESE R.	ALVITO (FR)
ASTARITA G.	NAPOLI	MACCHIONE G.	CAMPORA S.GIOVANNI (CS)
ATZORI C.	CAGLIARI	MAGNACCA F.	CAMPOBASSO
BALSAMO R.	S.MAURO TORINESE (TO)	MAGNO A.	NAPOLI
BANCHERO G.	GENOVA-QUARTO	MANCUSO F.	MESSINA
BARBIERI P.	LA SPEZIA	MANGELI S.	NOVOLI (LE)
BARRESI A.	CASTELVETRANO (TP)	MARIANI A.	ROMA
BELLANTE M.	CATANIA	MAVELLI G.M.	MARGHERITA DI SAVOIA (FG)
BELTRACCHINI G.	BERGAMO	MELE M.	LAIVES (BZ)
BIANCHI A.	COMO	MELLONE A.	NAPOLI
BONILAURI E.	MILANO	MONACHESI M.	ROMA
CAPOTORTO D.	BARI	MONTANARO C.M.	TORINO
CAPRIO A.	VENTICANO (AV)	MORRONI L.	MENTANA (RM)
CAPPUCCIO G.	SIRACUSA	MUTO P.	PERUGIA
CARBOGNO F.	MILANO	PALA L.	ALGHERO (SS)
CATALANI E.	FABRIANO (AN)	PICCHIAMI G.	TERNI
CAVANNA L.	GENOVA	PINI L.	BOLOGNA
CERQUAGLIA G.	SAPRI (SA)	PROPERZI P.	FERMO (AP)
CHIAVACCINI L.	CHIAVARI (GE)	PUCCI F.	TARANTO
CINTI R.	TORINO	RICOVERI G.	GHEZZANO (PI)
DAL LAGO A.	SCHIO (VI)	ROMBOLA' D.	SOVERATO (CZ)
DALLA ZORZA G.	PREGANZIOL (TV)	RONCHIN S.	MARTELLAGO (VE)
DE DEO L.	SULMONA (AQ)	ROSCIO G.	MONTANO LUCINO (CO)
DI TRAPANI E.	PALERMO	ROSSELLO S.	TAGGIA (IM)
DONZELLI F.	MILANO	ROSSI A.	VERONA
FERROLI E.	CATANIA	ROSSINI F.	BRESCIA
FIORITO L.	PIACENZA	RUTTER W.	UDINE
GAIONE G.	STAZZANO (AL)	SCARAMUZZA G.	ROMA
GARBELLINI L.	FERRARA	SEGHEZZI G.	VERDELLINO (BG)
GHIRALDELLI L.	MONTEBELLUNA (TV)	SICULIANA A.	PALERMO
GIAMBRONE L.	S.CATALDO (CL)	SOLLIMA G.	CATANIA
GIANSANTI A.	ROMA	SOSTER G.	TRIESTE
GIANSANTI S.	ROMA	SURACI G.	REGGIO CALABRIA
GIARDINI G.B.	TRAPPETO (CT)	TABACCO C.	MONFALCONE (GO)
GON P.	PADOVA	TABELLINI A.	RIMINI (FO)
GORLANI W.	BRESCIA	TRENTANI R.	PAVIA
GOVI A.	REGGIO EMILIA	VERONESI A.C.	BOLOGNA
GRAZIOLI G.	MILANO	VERPELLI A.	MONZA (MI)
GRAZIOSO S.	NAPOLI	VITIELLO C.	NAPOLI
JACOVAZZI G.	BARI	ZURLO A.	FAENZA (RA)

APPENDIX

A similar research comprising a controlled study of preventive type as well as a controlled study of therapeutic type, based on the same protocols used for the Italian research, is currently underway in Switzerland.

The following physicians have so far contributed to the study: M. Karly, Médecine générale FMH, Orbe (20 patients); I. Fumagalli, FMH Internal Medic. and Gastroenterology, Locarno (20 patients); P. Hüsler, A. Schenker and V. D'Apuzzo, Pediatrics, Hospital of the Blessed Virgin, Mendrisio (17 patients); R. Lodi, Geneva (16 patients).

The evaluation of the results, although limited by the small size of the sample population studied so far, is a further confirmation of the positive results observed in the multicentric Italian research in terms of both preventive and therapeutic efficacy of the preparation.

The results achieved are shown below in detail:

Studio controllato di tipo preventivo - Casi di comparsa della diarrea durante trattamento antibiotico nei due gruppi Placebo e Bioflorin:

	Assente	Lieve	Moderata	Severa	Totale
**PLACEBO**	11	4	1	1	6 su 17
**BIOFLORIN**	16	2	-	-	2 su 18

Studio controllato di tipo terapeutico - Valutazione mediante life-table della persistenza della diarrea durante trattamento rispettivamente con Placebo e con Bioflorin

Interv. di tempo (giorni)	**PLACEBO** - Numero di casi: inizio periodo	guariti	persi di vista	% stim. di pers. (inizio periodo)	**BIOFLORIN**-Numero di casi: inizio periodo	guariti	persi di vista	% stim. di pers. (inizio periodo)
0 - 1	18	1	-	100.0	18	2	-	100.0
1 - 2	17	2	-	94.4	16	2	-	88.9
2 - 3	15	2	-	83.3	14	3	-	77.8
3 - 4	13	3	1(*)	72.2	11	3	-	61.1
4 - 5	9	2	-	54.9	8	2	-	44.4
5 - 6	7	2	-	42.7	6	4	-	33.3
6 - 7	5	2	1	30.5	2	1	-	11.1
> 7	2	-	2	16.9	1	-	1(**)	5.6

ANALISI STATISTICA (WILCOXON TEST) W= -58; Z= -0.943

(*) Campylobacter jejuni
(**) Salmonella typhimurium
