# Supplementary figures and images for: Randomized, Placebo-Controlled, Double-Blind and Open-Label Studies in the Treatment and Prevention of Acute Diarrhea With Enterococcus faecium SF68
Source: Front Med (Lausanne). 2020 Jun 19;7:276. doi: 10.3389/fmed.2020.00276 (PMC7326027; doi:10.3389/fmed.2020.00276)

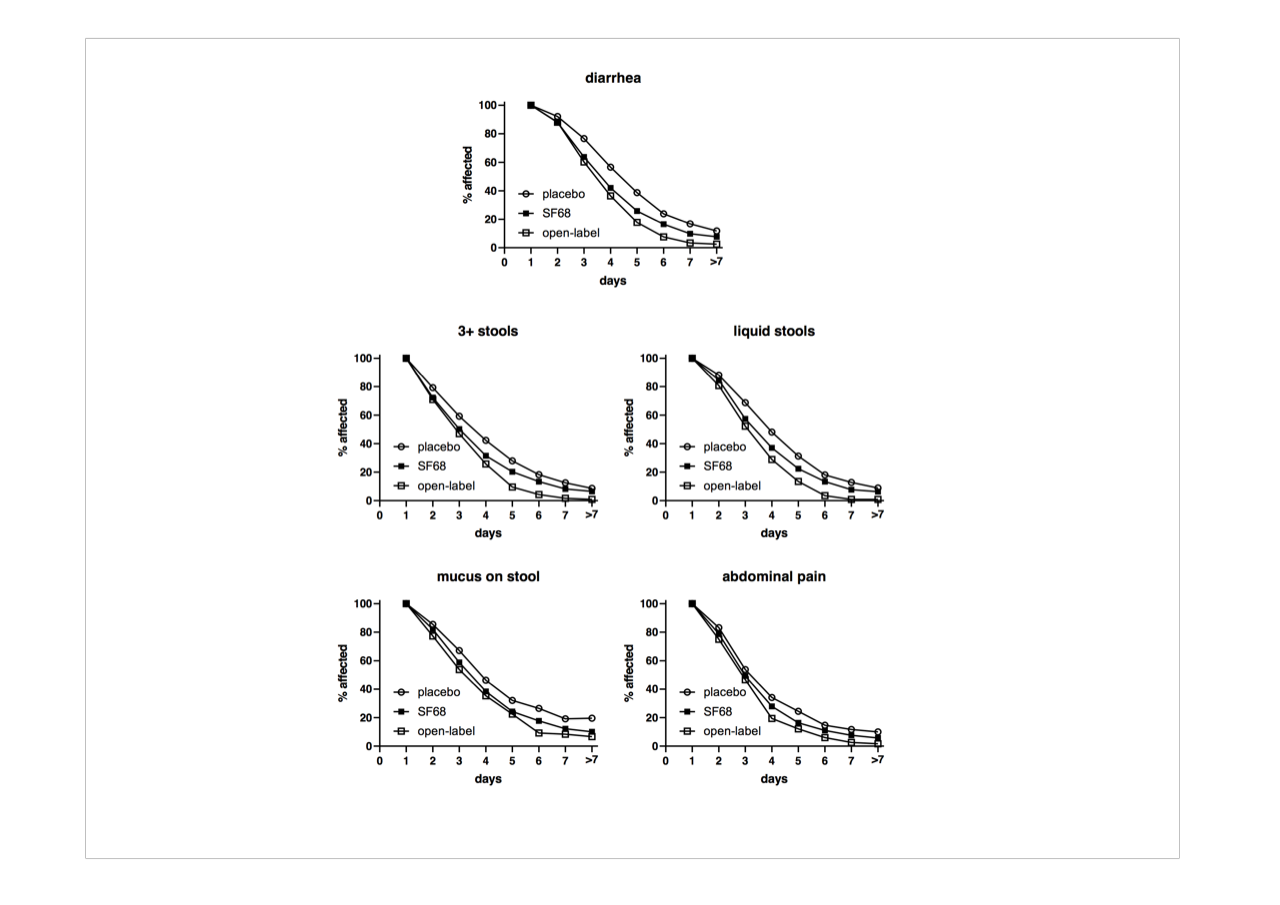

Supplement: Supplementary Figure 2 — Time to resolution of primary and secondary symptoms in the open-label treatment studies in comparison to the RCT. [file Image_1.TIFF]
